# Causality between immune cells and HER2-breast cancer: A 2‑sample Mendelian randomization study

**DOI:** 10.1097/MD.0000000000047054

**Published:** 2026-01-16

**Authors:** Mengdi Zhang, Dongqing Pu, Guangxi Shi, Jingwei Li

**Affiliations:** a Shandong University of Traditional Chinese Medicine, Jinan City, Shandong Province, China; b Department of Thyroid and Breast Diagnosis and Treatment Center, Affiliated Hospital of Shandong University of Traditional Chinese Medicine, Jinan City, Shandong Province, China.

**Keywords:** genetic association, HER2-breast cancer, immunophenotypes, Mendelian randomization, 2-sample

## Abstract

This study represents the first investigation employing 2-sample Mendelian randomization (MR), multi-marker analysis of genomic annotation (MAGMA), Metascape, and the Kaplan–Meier (K–M) plotter database to elucidate the causal relationship between immune cells (ICs) and human epidermal growth factor receptor 2 negative breast cancer (HER2-BC). The findings provide genetic evidence supporting the association between ICs and HER2-BC risk. The 2-sample data for the Mendelian randomization study were sourced from public databases. In this study, ICs were selected as the exposure factor, and HER2-BC was taken as the outcome factor. MR analysis was conducted on the causal relationship between ICs and HER2-BC by using various regression models. Gene-based analysis was carried out through MAGMA, and the gene functions and pathway enrichments of the genes identified through Metascape analysis were explored. Finally, based on the K–M plotter database, the survival status of some ICs was analyzed. Among the 731 ICs, a total of 33 ICs were found to have a protective effect on HER2-BC, while 17 ICs had an adverse effect. After false discovery rate-bonferroni (*P*_FDR_ < .05) correction, we detected 2 risk immunophenotypes of HER2-BC: human leukocyte antigen (HLA) DR on plasmacytoid DC, activated and secreting Treg %CD4+. A total of 38 genes were identified by MAGMA analysis. Metascape analysis revealed that the identified pleiotropic genes participated in negative regulation of cell migration, VEGFA-VEGFR2 signaling pathways. The survival analysis based on K–M plotter found that when CD4, HLA-DRB1, HLA-DRA, and ESR1 are highly expressed, the upper quartile survival rate of OS, RFS, and distant metastasis free survival is longer. This study showed that the immune response affects the progress of HER2-BC in a complex mode. These findings greatly improve our understanding of the interaction between immune response and HER2-BC risk, and also help to design therapeutic strategies for HER2-BC from the perspective of immunology.

## 1. Introduction

Human epidermal growth factor receptor 2 negative breast cancer (HER2-BC) is ineffective in anti-endocrine therapy and targeted therapy with HER2 gene positive because of the lack of corresponding receptor expression, with high invasiveness, high recurrence risk, poor prognosis and high metastasis rate,^[1]^ and among HER2 negative breast cancers, triple negative breast cancer (TNBC) is the most pessimistic one. TNBC is an invasive and immunogenic subtype of breast cancer, with negative expression of HER2, ER, and PR, accounting for 15% to 20% of all primary breast cancers. Compared with other BC subtypes, TNBC often appears in young women, and the mortality rate (40%) is higher in the first 5 years after diagnosis.^[2]^ Due to the lack of corresponding receptor expression, it seriously endangers the survival, quality of life and mental health of patients, especially female patients. The number of tumor-infiltrating lymphocytes, the expression level of programmed cell death-ligand 1 and tumor mutation burden and other indicators are higher than other subtypes of breast cancer, suggesting that TNBC has strong tumor microenvironment (TME) immune activity, which suggests that immunotherapy may be an effective treatment scheme.^[3]^

Nowadays, genome research is of great significance. The epigenome interacts with environmental factors such as nutrition and pathogens through mechanisms including DNA methylation and histone modifications, collectively regulating gene expression profiles and phenotypes.^[4]^ Substantial evidence indicates that epigenetic variations significantly impact health and productivity.^[5]^ Eukaryotic gene expression is spatiotemporally regulated in a multidimensional manner, exhibiting tissue specificity and developmental stage dependency, while gene product levels can further modulate expression through feedback mechanisms.^[6]^ Therefore, investigating trait-associated genes, proteins, and their activities at the cellular or chromosomal level constitutes a fundamental task in untangling the mechanisms of life.

The development of genome wide association study (GWAS) and Mendelian randomization (MR) methods makes it possible to evaluate the causality between immune cells (ICs) and HER2-BC. MR is a more reliable causal reasoning method. Using single nucleotide polymorphisms (SNPs) as a genetic tool to estimate the impact of related exposure on outcomes^[7]^ can overcome the limitations of observational studies. MR can be regarded as a natural randomized clinical trial. Compared with randomized clinical trial design, the biggest advantage of MR is that SNPs as a tool of risk factors are randomly assigned, which avoids the influence of potential confounding factors or reverse causality.^[8]^ In recent years, MR has been widely used as an alternative method to evaluate the causality between exposure and outcome.^[9]^ Many studies have proved the effectiveness of MR research in exploring the causality of autoimmune diseases.^[10,11]^ This study is the first to employ 2-sample MR, multi-marker analysis of genomic annotation (MAGMA), and Kaplan–Meier (K–M) survival analysis to investigate the causal relationship between ICs and HER2-BC. By identifying risk-associated immune phenotypes and their corresponding genes and signaling pathways from a genetic perspective, this research will provide genetic evidence for the development of immunology-based therapeutic strategies for HER2-BC.

## 2. Materials and methods

### 2.1. Study design

Based on the summary data set of large-scale GWAS data, this study used 2-sample MR method to evaluate the bidirectional causal relationship between 731 ICs in 7 immune panels and the risk of HER2-BC. All data come from published data, so there is no ethical issue involved. MR uses genetic variation to represent risk factors. According to STROBEMR statement, in order to ensure the validity of the results, effective instrumental variables in causal inference must meet 3 key assumptions: genetic variation is directly related to exposure; genetic variation has nothing to do with possible confounding factors between exposure and results; and genetic variation will not affect the results through ways other than exposure.

### 2.2. Data sources

In this study, ICs was selected as the exposure factor. SNPs data of ICs comes from IEU database (https://gwas.mrcieu.ac.uk/), and the summary statistics of GWAS of each IC can be publicly obtained from GWAS directory (accession numbers from GCST90001391 to GCST90002121).^[4]^ There were 731 ICs, including absolute cell count (n = 118), median fluorescence intensity reflecting surface antigen level (n = 389), morphological parameters (n = 32) and relative cell (RC) count (n = 192). Specifically, median fluorescence intensity, absolute cell, and RC features include B cells, cDCs, maturation stages of T cell, monocytes, myeloid cell, TBNK (T cells, B cells, and natural killer cells) and Treg panels, and morphological parameter features include CDC and TBNK panels. Four Illumina arrays (OmniExpress, ImmunoChip, Cardio-MetaboChip, and ExomeChip) were used to genotype the samples, and the whole genome was estimated based on the reference group of 3514 individuals from Sardinia sequence. Finally, about 22 million high-quality markers were reserved for association analysis, which was carried out after adjusting gender, age and age^2^ as covariates.^[12]^ HER2-BC is an outcome variable, and its SNPs data comes from IEU database (https://gwas.mrcieu.ac.uk/), including 1,23,579 samples (N_case_ = 7355, N_control_ = 1,16,224) and 1,63,79,784 SNPs. The research population includes women and men, and their genetic backgrounds are all of European descent, so as to avoid the bias caused by race-related confounding factors.

### 2.3. Instrumental variable (IV)

In order to find SNPs related to exposure factors and ensure the authenticity and accuracy of the causal connection conclusion between ICs and HER2-BC risks, the following steps are used to select the best SNPs.

One of the principles of MR is that there is no linkage disequilibrium among the selected SNPs. Firstly, by screening the GWAS data, the relevant SNPs met the requirement of *P* < 5 × 10^−8^. Then, independent and significant SNPs were extracted for each immune trait by using the clustering program in PLINK software, and the significance level was set to *P* < 1 × 10^5^.^[13,14]^ Within a distance of 500 kb, the threshold of linkage disequilibrium (linkage disequilibrium, LD) *r*^2^ is set to <0.1, where LD *r*^2^ is calculated based on the 1000 genome project reference group.^[15]^ For HER2-BC, the significance level was adjusted to 5 × 10^−8^ in this study. SNPs existed; the forward strand alleles were inferred using allele frequency information.

### 2.4. MR analysis

After coordinating the effect alleles of GWAS between ICs and HER2-BC, various methods, such as inverse variance weighted (IVW)^[16]^, MR-Egger, within-method of experimental design (WME),^[17]^ simple mode,^[18]^ weighted mode, MR-PRESSO method and so on, were used to detect the causality between ICs and HER2-BC. IVW uses meta-analysis method to combine the Wald estimation of each SNP to obtain the overall estimation of IC’s impact on HER2-BC. Studies show that IVW analysis is the most accurate method in MR when both pleiotropic and heterogeneous analysis are negative.^[19]^ MR-Egger is based on the instrument strength assumption which has nothing to do with the direct effect, which makes it possible to use the intercept term to evaluate the existence of pleiotropy. If the intercept term is equal to 0, it indicates that there is no horizontal pleiotropy, and the result of MR-Egger regression is consistent with IVW.^[20]^ WME assumes that half of the instrumental variables are valid, and analyzes the causal relationship between exposure and outcome. When up to 50% of tool variables are invalid, WME can correctly estimate causality. If the instrument strength assumption hypothesis is violated, compared with MR-Egger, the weighted mode has higher ability to detect causal effects, less bias and lower type I error rate.^[18]^ Where the effect allele frequency is the effective allele frequency of each SNP, and β is the effective value of the allele.^[21]^

*F*-statistic is used to evaluate the statistical strength of the correlation between SNPs and exposure, so as to evaluate IVs strength and avoid weak tool bias. *F* < 10, it is considered that there is a weak correlation between SNPs and exposure, so it is excluded from the analysis to avoid weak tool bias. *F*-statistic using the formula *F = *(β/se)^2^.^[22]^ If the corresponding *F*-statistic is >10, it is considered that there is no significant weak tool bias.^[23]^ Cochran *Q* statistics and corresponding *P*-values are used to test the heterogeneity between selected SNPs.^[16]^ In order to identify the potential heterogeneous SNP, the “leave-one-out” sensitivity-analysis was carried out by omitting each tool SNP in turn. In this study, MR-PRESSO is used to exclude horizontal variability outliers that may have a significant impact on the results.^[24]^ In order to evaluate the causality between HER2-BC and ICs, the ICs found to be causal with HER2-BC in forward MR analysis was analyzed by reverse MR. The methods and settings adopted are consistent with the forward MR.

The *q*-value program is used to correct the false discovery rate (FDR), and the FDR is *q* < 0.1.^[25]^ When *P* < .05 but *q* ≥ 0.1, ICs and HER2-BC are considered to have suggestive correlation.

PhenoScanner (https://maayanlab.cloud/datasets2tools/landing/tool/PhenoScanner) was used to search all qualified SNPs, and the heterogeneity test eliminated the significantly heterogeneous SNPs. Finally, the effective SNPs significantly related to HER2-BC were obtained as instrumental variable.

All the analyses were carried out with R (version 4.3.1; Lucent), and MR analysis was carried out with “Two Sample MR (version 0.5.6),” “Mendelian Randomization (version 0.4.3; England)”^[26]^ and “MR-PRESSO (version 1.0)”.

### 2.5. MAGMA analysis

In order to further explore the possible mechanism of HER2- breast cancer and ICs, whether the identified ICs has the characteristics of pleiotropic gene. Gene-based analysis is carried out by MAGMA (v.1.10), which can quickly aggregate a group of SNPs level associations into a single gene level association signal based on GWAS summary statistics.^[27]^ Based on pleiotropic score, pleiotropic genes related to ICs and HER2-BC risk were determined. Finally, further gene and pathway enrichment analysis was carried out based on Metascape.^[28]^

### 2.6. Survival analysis based on Kaplan–Meier plotter

K–M plotter evaluated the survival rates of different genes in 21 cancer types, including breast cancer (N = 6234), ovarian cancer (N = 2190) and lung cancer (N = 3452) et.al.^[29]^ K–M plotter was used to evaluate the survival analysis and prognostic value of ICs for HER2-BC, including recurrence free survival, distant metastasis free survival and overall survival (OS). Hazard ratios with 95% confidence intervals (CI) and log rank *P*-value were determined.

## 3. Results

### 3.1. MR analysis

**Figure 1.**
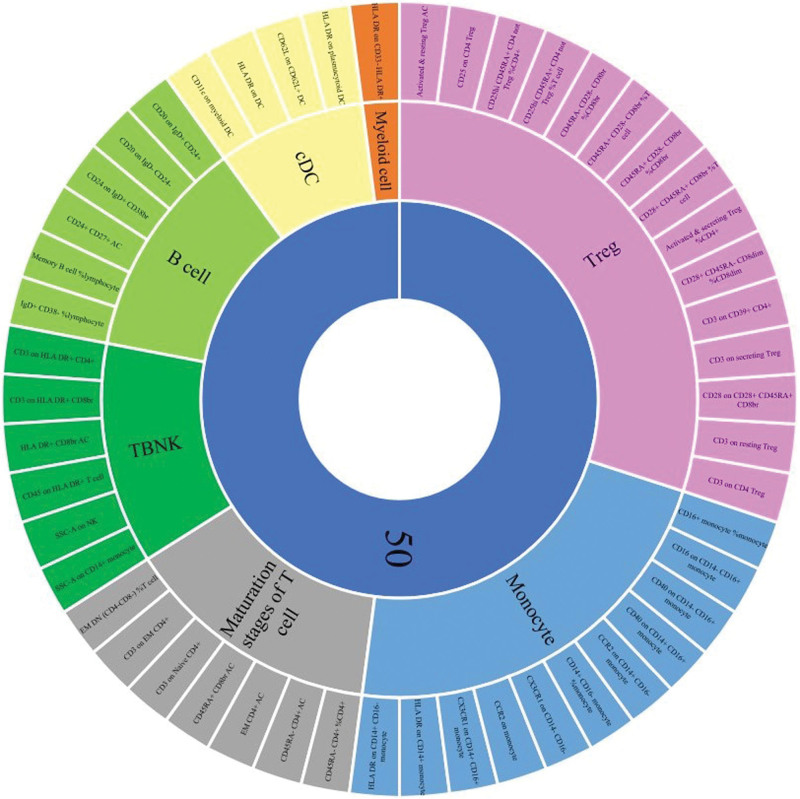
Distribution of significant immune cells at nominal significant level in different trait types and different panels (causal effects of immune cells on HER-BC risk).

At the nominal significance level, we detected the causality of 50 ICs in HER2-BC (Table S1, Supplemental Digital Content, https://links.lww.com/MD/R109). The increase of 17 ICs and the decrease of 33 ICs can induce the risk of HER2-BC. These 50 ICs are distributed in B cells (6 cells), cDC (4 cells) and T cells at maturity (7 cells), monocytes (11 cells), bone marrow cells (1 cell), TBNK (6 cells) and Treg (15 cells; Fig. 1).

**Figure 2.**
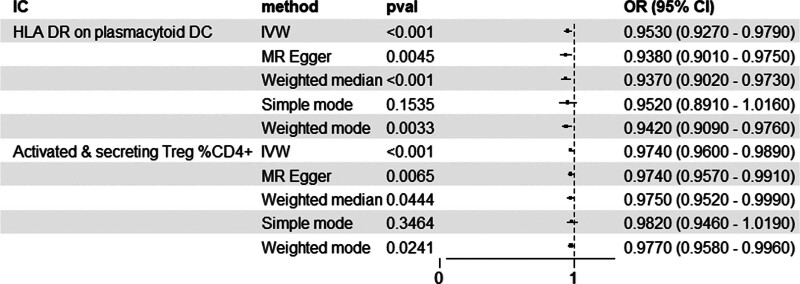
Causal associations between 2 identified immune phenotypes and HER2-BC risk by using different methods. CI = confidence intervals, IC = immune cell, IVW = inverse variance weighted, MR = Mendelian randomization.

**Figure 3.**
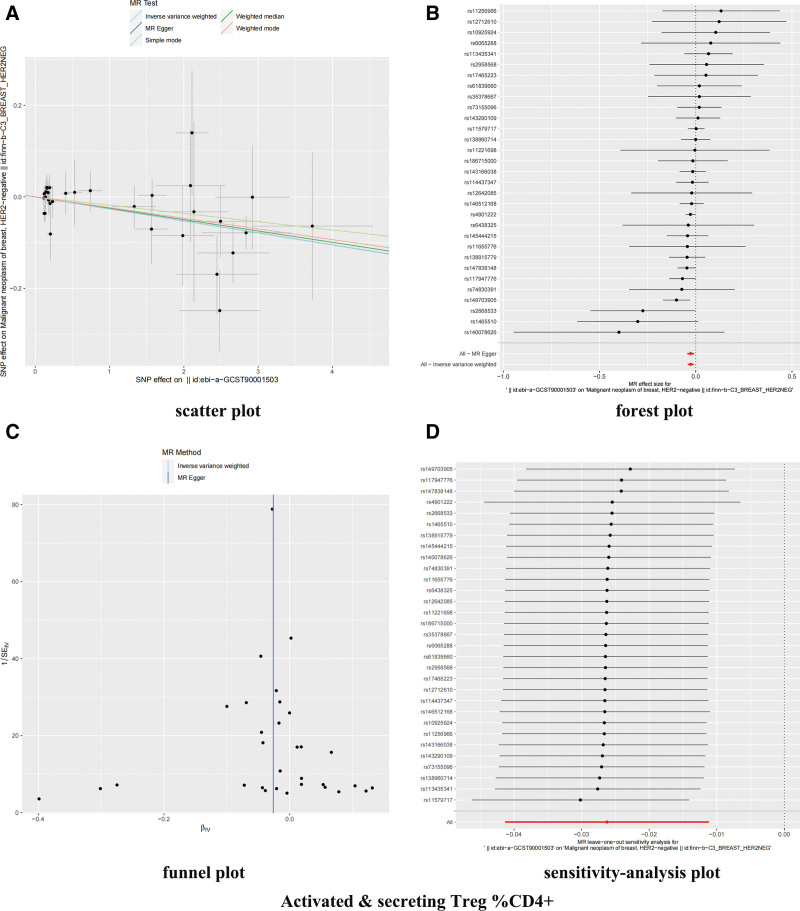
Forest, scatter, funnel, and sensitivity-analysis plot of activated and secreting Treg %CD4+. (A) Scatter plot to visualize causal effect of ICs on HER2-BC risk. The slope of the straight line indicates the magnitude of the causal association. (B) Forest plots showing β (±standard error) and P-values of the single-SNP 2-sample MR analysis between ICs and HER2-BC. (C) Funnel plots to visualize overall heterogeneity of MR estimates for the effect of ICs on HER2-BC. (D) Leave-one-out plot to visualize causal effect of ICs on HER2-BC risk. IC = immune cell, MR = Mendelian randomization, SNP = single nucleotide polymorphism.

**Figure 4.**
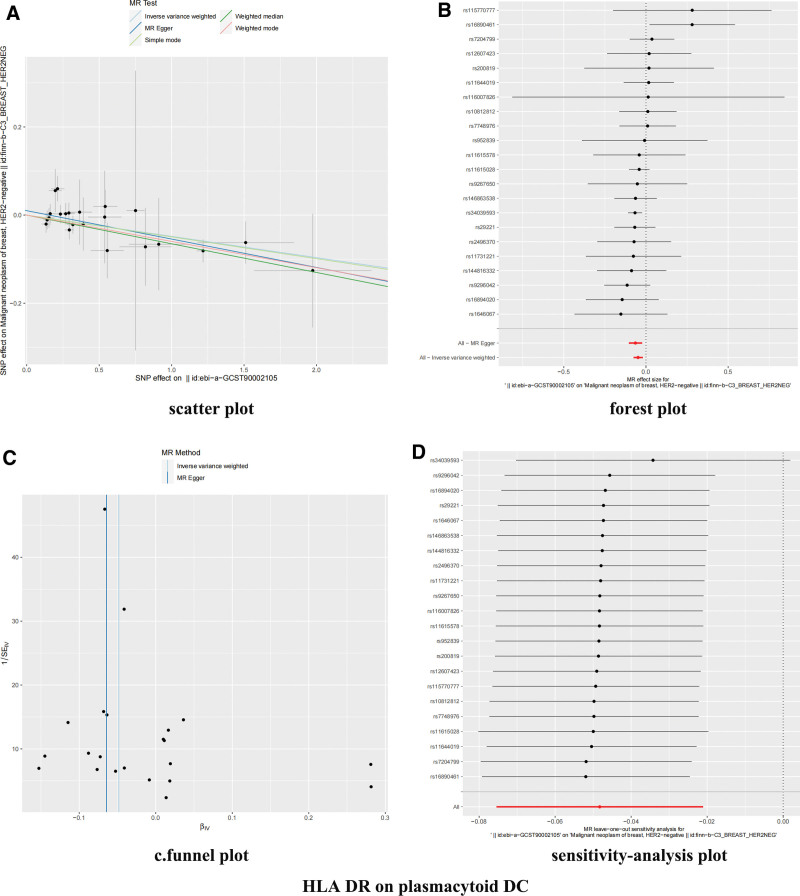
Forest, scatter, funnel, and sensitivity-analysis plot of HLA DR on plasmacytoid DC. (A) Scatter plot to visualize causal effect of ICs on HER2-BC risk. The slope of the straight line indicates the magnitude of the causal association. (B) Forest plots showing β (±standard error) and *P*-values of the single-SNP 2-sample MR analysis between ICs and HER2-BC. (C) Funnel plots to visualize overall heterogeneity of MR estimates for the effect of ICs on HER2-BC. (D) Leave-one-out plot to visualize causal effect of ICs on HER2-BC risk. HLA = human leukocyte antigen, IC = immune cell, MR = Mendelian randomization, SNP = single nucleotide polymorphism.

After correction by FDR (bonferroni) method, it was observed that the 2 immunophenotypes were still significant (Fig. 2 and Table S2, Supplemental Digital Content, https://links.lww.com/MD/R109). Forest plots showing β (±standard error) and *P*-values of the single-SNP of 2-sample MR analysis between HER2-BC and ICs (Figs. 3B and 4B). In the cDC group, the odds ratio (OR) of HLA DR on plasmacytoid DC was estimated to be 0.9530 (95% CI: 0.9270–0.9790, *P* = .000488, *P*_FDR_ = .024), and similar results were observed by other methods: MR-Egger (*P* = .00444). The OR of weighted median (*P* = .000683), weighted mode (*P* = .003304), MR-PRESSO (*P* = .000466) and activated and secreting Treg %CD4+ is estimated to be 0.9740 (95 CI: 0.9600–0.9890). *P* = .000635, *P*_FDR_ = .031), and consistent results were also observed by using the other 4 methods: MR-Egger (*P* = .006513), weighted median (*P* = .044445), weighted mode (*P* = .02409), and MR-PRESSO (*P* = .000590).

Cochran *Q* test results showed that these IVs have no significant heterogeneity (Table S3, Supplemental Digital Content, https://links.lww.com/MD/R109). In addition, both the intercept of MR-Egger (Table S4, Supplemental Digital Content, https://links.lww.com/MD/R109) and the global test of MR-PRESSO (Table S5, Supplemental Digital Content, https://links.lww.com/MD/R109) excluded the possibility of horizontal pleiotropy. The scatter plots (Figs. 3A and 4A) and funnel plots (Figs. 3C and 4C) also proved the stability of the results. The detailed information of sensitivity-analysis proved the robustness of the observed causality (Figs. 3D and 4D).

### 3.2. Exploration of the causal effect of HER2-BC onset on immunophenotypes

In order to determine how the development of HER2-BC affects the immune mechanism of the body, we conducted a reverse MR analysis to explore the causal influence of HER2-BC on ICs. After adjusting for multiple tests, we found no causality reaching the nominal significant level of 0.05. The results showed that there is no significant causality between HER2-BC and ICs.

### 3.3. Gene analysis based on MAGMA and Metascape

**Figure 5.**
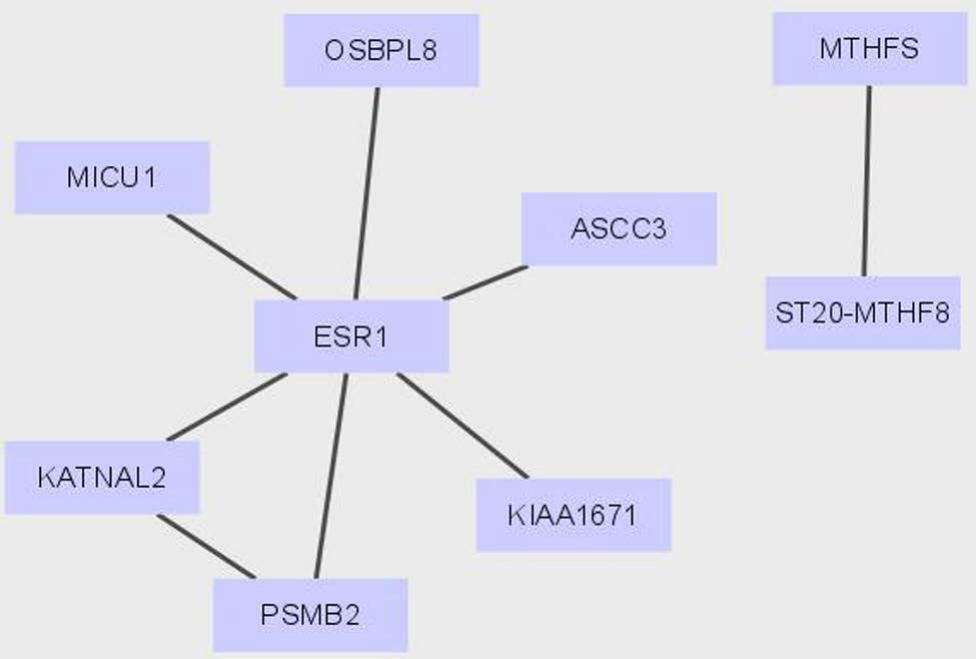
Protein–protein interaction enrichment analysis. The resultant network contains the subset of proteins that form physical interactions with at least one other member in the list.

**Figure 6.**
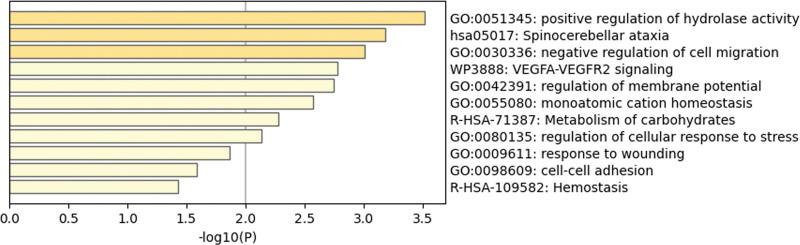
Bar graph of enriched terms across input gene lists, colored by *P*-values.

**Figure 7.**
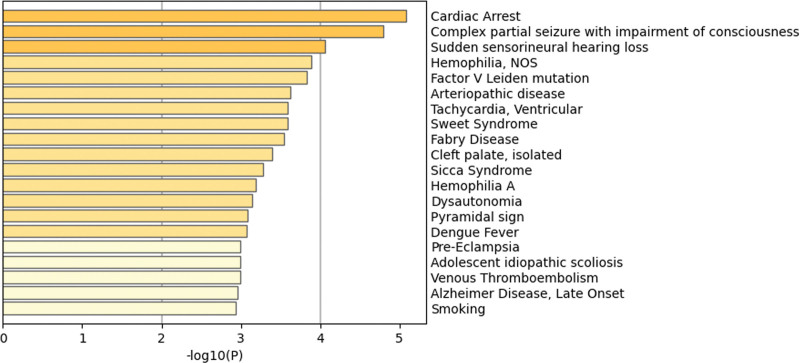
Summary of enrichment analysis in DisGeNET.

According to the results of GWAS analysis, there were 1518 SNPs and the *P*-value of HER2-BC for gene pleiotropic analysis (Table S6, Supplemental Digital Content, https://links.lww.com/MD/R109). Finally, according to the results of MAGMA, a total of 38 genes were used for further pathway enrichment analysis (Table S7, Supplemental Digital Content, https://links.lww.com/MD/R109). Protein–protein interaction network analysis (PPI) and Kyoto Encyclopedia of Genes and Genomes showed that there was interaction, and ESR1 was the key target (Fig. 5). Finally, the negative regulation of cell migration, VEGFA-VEGFR2 signaling and other ways were determined (Fig. 6). In addition, through the information analysis of DisGeNET gene set, it was found that these genes were also enriched and acted on a large number of diseases (Fig. 7).

### 3.4. Survival analysis based on Kaplan–Meier plotter

**Figure 8.**
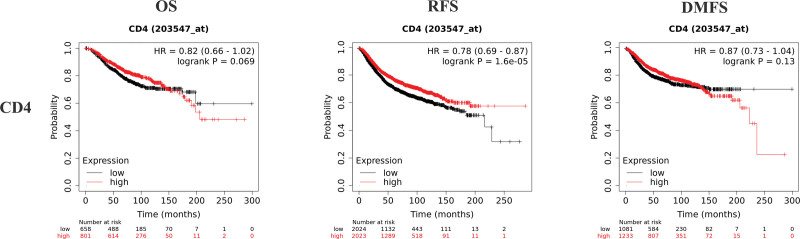
Survival curves of OS, RFS and DMFS of CD4 based on K–M plotter. DMFS = distant metastasis free survival, K–M = Kaplan–Meier, OS = overall survival, RFS = recurrence free survival.

**Figure 9.**
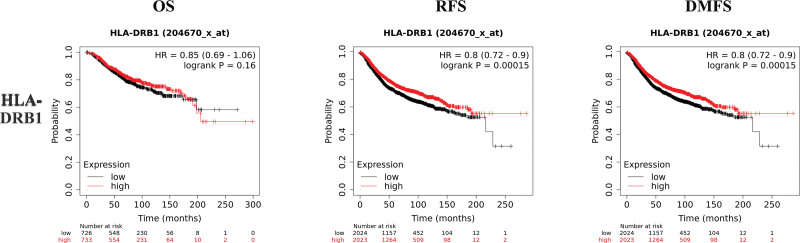
Survival curves of OS, RFS and DMFS of HLA-DRB1 based on K–M plotter. DMFS = distant metastasis free survival, HLA = human leukocyte antigen, K–M = Kaplan–Meier, OS = overall survival, RFS = recurrence free survival.

**Figure 10.**
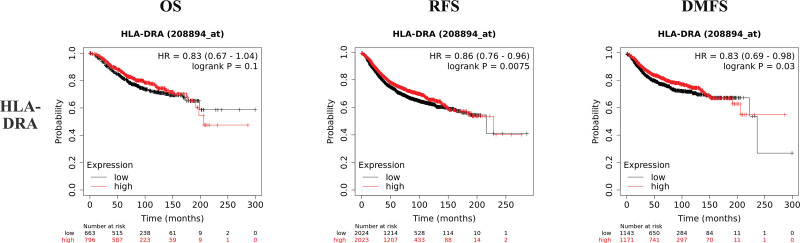
Survival curves of OS, RFS and DMFS of HLA-DRA based on K–M plotter. DMFS = distant metastasis free survival, HLA = human leukocyte antigen, K–M = Kaplan–Meier, OS = overall survival, RFS = recurrence free survival.

**Figure 11.**
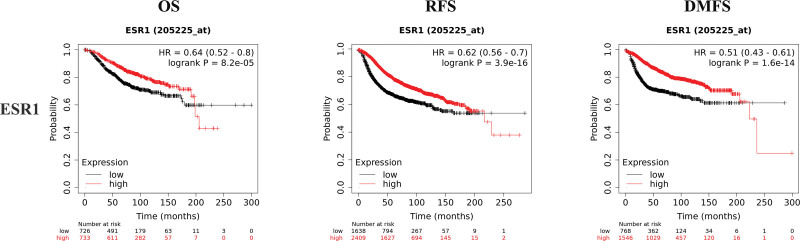
Survival curves of OS, RFS and DMFS of ESR1 based on K–M plotter. DMFS = distant metastasis free survival, K–M = Kaplan–Meier, OS = overall survival, RFS = recurrence free survival.

K–M plotter is an index to evaluate the prognostic value of CD4, HLA-DRB1, HLA-DRA, and ESR1 expression based on Affymetrix microarray. The mRNA levels of CD4, HLA-DRB1, HLA-DRA, and ESR1 were positively correlated with OS, recurrence free survival and distant metastasis free survival in HER2-BC patients (Figs. 8–11 and Table 1). The data showed that CD4, HLA-DRB1, HLA-DRA, and ESR1 are potential biomarkers for predicting the prognosis of HER2-BC.

**Table 1:** The upper quartile survival of CD4, HLA-DRB1, HLA-DRA, ESR1, and Foxp3 (months).

		OS	RFS	DMFS
CD4	H	123.6	68.4	111
	L	86	45.37	78.32
HLA-DRB1	H	143	66	111
	L	95.64	45.9	80.68
HLA-DRA	H	121.2	118.62	116
	L	90	72.18	78.02
ESR1	H	143	76	137
	L	77.4	32.03	34.8

DMFS = distant metastasis free survival, H = high expression, HLA = human leukocyte antigen, L = low expression, OS = overall survival, RFS = recurrence free survival.

## 4. Discussion

According to the latest global cancer data in 2023,^[30]^ breast cancer accounts for about 31% of female malignant tumors, accounting for 15% of female malignant tumor mortality, and the incidence rate is increasing at an annual rate of about 0.5%. HER2-BC has limited treatment options because of the lack of corresponding receptor expression.^[31]^ High recurrence risk, poor prognosis and high metastasis rate pose challenges to the physical and mental health, economic burden, OS and quality of life of BC patients.^[32]^

This study systematically revealed the mechanism of ICs in the occurrence and development of HER2-BC from the perspective of genetics by using the ICs summary statistics and HER2-BC data of GWAS meta-analysis. This study provided suggestive evidence that ICs can affect HER2-BC risk through comprehensive heredity based on large-scale GWAS summary data. Using SNPs as a tool variable, 2-sample MR method proved that 2 kinds of ICs (HLA DR on plasmacytoid DC, activated and secreting Treg %CD4+) were related to the risk of HER2-BC. Metascape results showed that ESR1 was the key target that acted on HER2-BC through immunophenotype. The survival analysis results of K–M plotter were also consistent with those of MR analysis, that is, CD4, HLA-DRB1, HLA-DRA, and ESR1 were favorable factors for HER2-BC, and their expressions were positively correlated with the prognosis.

The occurrence of malignant tumor is closely related to immune imbalance. Disordered immune system and low immune function can cause tumor cells to escape immune surveillance and IC killing, and cause malignant proliferation, invasion or metastasis of tumor cells.^[33]^ As a foreign body, tumor can be recognized, rejected, and cleared by the body’s immune system. If the immune system is suppressed, tumor cells can escape immune surveillance and escape, thus promoting malignant proliferation and invasion of tumor.^[34]^ In 2002, oncologist Schreiber put forward the theory of tumor immunoediting.^[35]^ Cancer cells can reshape the biological characteristics of ICs, escape immune surveillance, and make tumor cells escape and metastasize. The complexity of IC composition in breast cancer reflects the “crosstalk” between innate immune response components, because it regulates the polarity of TME and adaptive immune response in tumor.^[36]^

ICs in normal breast tissue are mainly located in epithelial components of ductal lobules of breast. There are IC subtypes representing innate immunity (NK, CD68+ and CD11c+ cells) and adaptive immunity (CD4+, CD8+ and CD20+).^[37]^ These ICs together provide important innate and adaptive immunity for the epithelial layer to resist exogenous and endogenous substances and eliminate transformed cells. ICs in normal breast tissue may play an important role in immune monitoring, and the infiltration of ICs increases in breast cancer, which has important prognostic and therapeutic effects.^[38]^ CD4+ exists in breast proliferative diseases and increases in parenchyma and matrix with the progress of breast cancer.^[39]^ The progression to breast cancer is characterized by increased infiltration of ICs in tumor parenchyma and matrix, including CD4+ and CD8+ granzyme B+ cytotoxic T cells, B cells, macrophages and dendritic cells.^[38]^ The detailed analysis of TILs has proved the prognostic characteristics of ICs.^[40]^ Therefore, understanding the IC population in normal breast tissue may be of great significance for breast cancer prevention, improving risk assessment methods and regulating breast cancer occurrence.

The existence of CD4+ regulatory T cells is related to the good or bad prognosis of HER2-BC.^[41]^ In a study of mastectomy specimens by immunohistochemistry and flow cytometry, Ruffell et al found that compared with normal breast tissues, tumors in tumors turned into Th2-type reactions of breast cancer, which was characterized by the increased presence of CD4+ T cells and B cells.^[42]^ Boieri et al proved that CD4 T cells can play an anti-proliferation role by blocking the progress of cancer cell cycle at G1/S, and can also directly prevent the development of breast cancer by forcing cancer cells to differentiate at the terminal stage.^[43]^ In addition, CD4 T cells alone inhibited the growth of HER2-BC in vivo when T cell subsets were adoptive transferred to humanized breast cancer mouse model. RNA microarray analysis showed that CD4 T cells can significantly reduce the progress and proliferation of tumor cell cycle.^[44]^

With the deepening of tumor immunology research, it is found that the imbalance of CD4+ T cell subsets is closely related to the occurrence of tumors.^[45]^ Tregs is a group of CD4+ T cell subsets that control autoimmune reaction, and inhibits abnormal autoimmune reaction by secreting IL-10 and TGF-β, so as to maintain immune homeostasis.^[46]^ Among them, effector regulatory T cells have immunosuppressive function.^[47]^ In tumor immunity, Treg cells participate in the occurrence and development of tumors by inducing immune tolerance and inhibiting antitumor immune response.^[48]^ Tregs can promote the immune escape of tumor cells in many ways, such as secreting immunosuppressive cytokines to exert immunosuppressive effect.^[49]^ The research of Li et al^[50]^ also showed that Tregs was significantly correlated with the clinical T stage of BC, suggesting that Tregs may be involved in the immunosuppression process of BC tumor. In all kinds of malignant tumors, high level of Tregs is related to the lower survival rate of patients. Inhibition of Tregs function and increase of antitumor immune response may become the main direction of tumor immunotherapy.^[51]^

The epidemiological characteristics of BC have obvious regional differences, racial and ethnic differences and family history, which are similar to the genetic characteristics of human leukocyte antigen (HLA).^[52]^ HLA allele polymorphism is an important genetic factor that determines the ability of immune response, and different HLA allele polymorphisms will affect the degree of individual immune response.^[53]^ HLA system is the major histocompatibility complex (MHC) and the decisive factor of immune response.^[54]^ HLA is the most complex and polymorphic gene in human beings, which is related to the occurrence of various tumors and has always been the focus of tumor immunity research.^[55]^

HLA-DR is a class II MHC molecule, which is mainly expressed in antigen presenting cells such as macrophages and dendritic cells.^[56]^ HLA-DR presents exogenous antigens to CD4 Tregs, helper T cells and suppressor T cells, and participates in the immune reaction process.^[57]^ CD4+ and CD8+ T cells activated by HLA new antigen complex will secrete various pro-inflammatory cytokines, promote the maturation of other lymphocytes and block the development of Tregs and tumor-associated macrophages to further activate antitumor immunity.^[58]^ Recent studies show that HLA-II gene may also be expressed in BC cells, and its expression level is similar to that of ICs. The tumor expressing HLA-II grows slower than the control group, and more functional CD4+ and CD8+ T cells are recruited.^[59]^ The level of HLA-DR in cytotoxic T lymphocytes is an independent and robust predictor of the response of BC patients to neoadjuvant chemotherapy.^[60]^ HLA-DR+ cytotoxic T lymphocytes mainly exist in TME, and produce high-level cytotoxic related molecules, which are negatively correlated with the immunosuppressive characteristics of tumor environment and are systematically reflected.^[61]^

HLA-DR gene expresses 2 HLA protein subunits (HLA-DRA and HLA-DRB) with molecular weights of 36 and 27 kD, corresponding to α chain and β chain, belonging to MHC-II molecules.^[62]^ HLA-DRB1 gene has the most abundant polymorphism in HLA-II gene, which is the decisive factor of the polymorphism of immune antigen. It mainly participates in the immune process of tumor body and affects the occurrence and development of various tumor diseases. As an immune effector molecule, HLA-DRB1 plays an important role in the occurrence and development of breast cancer. By studying the association between HLA-DRB1 and BC, it is helpful for early diagnosis, early treatment and prognosis of BC.^[56]^ The analysis of 1182 immune-related genes showed that HLA-DRA was inversely related to the recurrence of ER-BC, and the expression characteristics of HLA-DRA had a higher prognosis for ER-BC and TNBC.^[63]^

Estrogen plays a vital role in the occurrence of BC.^[64]^ ER is a ligand-dependent transcription factor. When estradiol binds to its ligand-binding region, it can lead to conformational changes of ER, thus recruiting co-regulatory proteins to regulate gene transcription and promote the growth, proliferation, and survival of tumor cells.^[65]^ ER has 2 subtypes, ERα and ERβ, which are encoded by different genes and regulate different physiological functions. Estrogen receptor α (ESR1) is a gene encoding ERα protein, which is a key driver of the development of ER+ breast cancer.^[66]^ However, ERβ has broad-spectrum tumor inhibitory activity, which regulates the opposite effect of ERα in breast cancer cell lines.^[64]^ ERα, encoded by ESR1 gene, belongs to the nuclear receptor superfamily, and can directly bind DNA as a transcription factor.^[67]^ ERα promotes the occurrence and development of BC mainly through the binding of excitatory ligand (estrogen) in vivo and the activation and regulation of gene transcription mediated by cell signaling pathway.^[68]^

Studies have shown that ESR1 mutations are rare in the initial stage of cancer (<1%), and most of them occur in the metastatic stage.^[69]^ Clinical studies have shown that ESR1 mutation can preexist in the primary tumor and be enriched in the process of metastasis. In addition, the expression of ESR1 mutation is beneficial to the unique transcription profile of tumor progression, indicating that the selected ESR1 mutation may affect metastasis.^[70]^ Transcriptional analysis from this study^[70]^ and other study^[71]^ shows that ESR1 mutation promotes the up-regulation of Hallmark cancer pathway, including estrogen response, p53 pathway and MTORC1 signal transduction, which indicates the role of mutant ER in promoting ET drug resistance and metastatic phenotype. Recently, Xu et al^[72]^ found that ERα can regulate the mRNA selective shearing and translation of key factors in cell pressure response pathway, up-regulate the expression of stress protein, and promote the survival of breast cancer cells in TME stress. In 1997, Zhang et al^[73]^ discovered the mutation of the LBD domain of ESR1 gene for the first time in metastatic breast cancer samples, and proved that this mutant has continuous transcription activity independent of estrogen. Later, it was found that there was ESR1 gene mutation in about 36% of metastatic ER+ BC,^[74]^ and most mutations were detected in patients treated with AIs.^[75]^ The emergence of ERα mutant will reduce the sensitivity of patients to endocrine therapy, which is mainly related to the ligand-independent activation, and at the same time, the affinity for drugs will decrease to varying degrees. Most mutations of ESR1 gene appear in metastatic tumors treated by AIs, suggesting that mutations are related to the generation of cell metastasis phenotype.^[76]^

Our research has some limitations. First of all, ICs data and HER2-BC data of GWAS summary data set come from different studies, and there are some differences in sample size, quality control methods and race. Therefore, we emphasize the necessity of carefully explaining the results of this study. Secondly, this study is based on the aggregate level data set, not including the individual level data, so this study cannot further study some characteristics (such as gender, age, etc). Thirdly, despite the FDR multiple correction, the loose threshold selected by SNPs may lead to a certain degree of false positive.

In the whole carcinomatous conversion process, ICs is gradually recognized in breast tissue, starting from the normal breast tissue of women at normal risk of BC, and continuing to the primary tumor and metastasis of BC. ICs in normal breast tissue may play an important role in immune monitoring. These findings allow immunology and immunotherapy to be the fourth main way to treat cancer patients, alongside surgery, chemotherapy, and radiotherapy. This will greatly improve the possibility of preventing and treating BC. In a word, the results of this study prove the potential causal role of ICs in HER2-BC. It provides an important auxiliary function for clinical judgment of disease prognosis and treatment, and also provides a direction for the development of new drugs. However, the pathogenesis of HER2-BC is complex, and the clinical heterogeneity of different types of ICs participating in HER2-BC is obvious, so single treatment often cannot achieve good results. Therefore, the interaction between innate ICs and the interaction between innate ICs and adaptive ICs in HER2-BC patients’ needs further study. In a word, we believe that our research results can provide new insights for the immunology of HER2-BC, and more and more rigorous experimental studies should be carried out in the next step to further explore the potential mechanism between ICs and the risk of HER2-BC.

## 5. Conclusion

This study, for the first time, employs advanced analytical methods, including MR, to overcome the limitations of traditional observational studies and systematically investigate the causal relationship between ICs and HER2-BC at the genetic level. Key IC subsets, risk phenotypes, genes, and signaling pathways were identified. By characterizing protective and risk-associated IC phenotypes, potential immunotherapeutic targets for HER2-BC were revealed. Furthermore, survival analysis was conducted to evaluate their prognostic value, thereby optimizing the patient outcome assessment system.

This research not only deepens the understanding of the interaction mechanisms between immune responses and HER2-BC but also provides critical genetic evidence for immunotherapeutic target selection, facilitating the development of novel treatment strategies. In the future, based on these findings, further exploration may focus on modulating key ICs and signaling pathways to achieve precision immunotherapy for HER2-BC, ultimately improving patient prognosis, reducing mortality rates, and offering new hope for overcoming this refractory malignancy.

## Acknowledgments

We thank all the consortiums for making the summary association statistics data publicly available, and we are grateful to the participants and many researchers involved in proteome GWAS studies.

## Author contributions

**Conceptualization:** Mengdi Zhang, Dongqing Pu, Jingwei Li.

**Data curation:** Dongqing Pu, Guangxi Shi.

**Formal analysis:** Mengdi Zhang.

**Funding acquisition:** Jingwei Li.

**Visualization:** Mengdi Zhang, Dongqing Pu.

**Writing – original draft:** Mengdi Zhang.

**Writing – review & editing:** Guangxi Shi, Jingwei Li.

## Supplementary Material


