# Supplementary material for: Causality between immune cells and HER2-breast cancer: A 2‑sample Mendelian randomization study
Source: Medicine (Baltimore). 2026 Jan 16;105(3):e47054. doi: 10.1097/MD.0000000000047054 (PMC12826194; doi:10.1097/MD.0000000000047054)
Supplement: Supplementary file 1 [file medi-105-e47054-s001.docx]

**Table S1 Instrumental variables used in MR analysis of the association between Immune Cells and Her2- Breast Cancer.**

| Immune Cells  (exposure) | SNP | effect_  allele | other_  allele | Exposure | | | Outcome | | |
| --- | --- | --- | --- | --- | --- | --- | --- | --- | --- |
|  |  |  |  | beta | se | pval | beta | se | pval |
| CD24+ CD27+ AC | rs10759006 | C | T | 0.1133 | 0.02556 | 9.51E-06 | -0.005 | 0.0192 | 0.7949 |
|  | rs10762541 | T | C | 0.1488 | 0.02978 | 6.09E-07 | -0.0259 | 0.0196 | 0.1863 |
|  | rs10942726 | G | A | 0.1188 | 0.02672 | 8.93E-06 | 0.0063 | 0.0196 | 0.749501 |
|  | rs11649808 | A | G | 0.1546 | 0.03472 | 8.75E-06 | 0.0032 | 0.0192 | 0.8692 |
|  | rs11653761 | T | G | -0.118 | 0.0247 | 1.84E-06 | 0.0011 | 0.0186 | 0.9513 |
|  | rs118092259 | G | T | -0.2901 | 0.06345 | 4.98E-06 | 0.2245 | 0.1057 | 0.03371 |
|  | rs12874404 | G | A | 0.1691 | 0.02653 | 2.04E-10 | 0.0096 | 0.0504 | 0.8484 |
|  | rs138329462 | A | G | -0.4374 | 0.09365 | 3.11E-06 | 0.095 | 0.0721 | 0.1879 |
|  | rs144295598 | A | G | 0.5664 | 0.1203 | 2.60E-06 | 0.1567 | 0.1026 | 0.1266 |
|  | rs144625305 | T | C | 0.6162 | 0.1357 | 5.75E-06 | -0.0171 | 0.0809 | 0.8324 |
|  | rs149085540 | T | C | -0.4318 | 0.09574 | 6.67E-06 | 0.1344 | 0.1941 | 0.4889 |
|  | rs16867231 | A | G | 0.5129 | 0.1154 | 8.98E-06 | -0.0178 | 0.0577 | 0.7571 |
|  | rs17708396 | A | C | 2.539 | 0.5201 | 1.09E-06 | -0.0748 | 0.0382 | 0.05024 |
|  | rs184496770 | T | C | 1.162 | 0.2608 | 8.56E-06 | -0.2918 | 0.2536 | 0.2499 |
|  | rs35057381 | C | T | 0.1344 | 0.02969 | 6.18E-06 | 0.0175 | 0.0194 | 0.3683 |
|  | rs4076316 | A | T | -0.1404 | 0.02729 | 2.84E-07 | -0.001 | 0.0188 | 0.9568 |
|  | rs56033406 | T | C | 0.312 | 0.06692 | 3.23E-06 | 0.055 | 0.0561 | 0.3271 |
|  | rs7254039 | A | G | -0.2606 | 0.0585 | 8.64E-06 | 0.0032 | 0.0396 | 0.9363 |
|  | rs728776 | G | A | -0.1712 | 0.03808 | 7.09E-06 | 0.0209 | 0.0334 | 0.5309 |
|  | rs73192651 | T | C | 0.4411 | 0.08893 | 7.38E-07 | -0.1404 | 0.0728 | 0.05383 |
|  | rs75766272 | T | C | -0.4055 | 0.08609 | 2.57E-06 | 0.0135 | 0.0354 | 0.702999 |
|  | rs7640784 | A | C | -0.1111 | 0.02476 | 7.48E-06 | -0.0233 | 0.02 | 0.243 |
|  | rs76552872 | T | G | 0.283 | 0.06219 | 5.54E-06 | -0.1242 | 0.1206 | 0.303 |
|  | rs77762131 | C | G | 0.2815 | 0.05774 | 1.13E-06 | 0.1821 | 0.1057 | 0.084959 |
|  | rs79207264 | A | G | 0.1752 | 0.0393 | 8.47E-06 | -0.1114 | 0.044 | 0.01136 |
| IgD+ CD38- %lymphocyte | rs10444793 | A | G | -0.4323 | 2.89E-06 | 0.09225 | 0.1735 | 0.0714 | 0.01517 |
|  | rs112782756 | G | A | 3.09 | 6.25E-06 | 0.683 | 0.191 | 0.0799 | 0.01686 |
|  | rs12146653 | C | T | 0.1892 | 4.66E-06 | 0.04126 | -0.0375 | 0.0327 | 0.2517 |
|  | rs12197754 | C | T | -0.1427 | 4.75E-06 | 0.03115 | -0.0391 | 0.0199 | 0.04966 |
|  | rs12763172 | C | T | 0.1272 | 5.05E-06 | 0.02784 | 0.0061 | 0.0183 | 0.740901 |
|  | rs12829274 | G | A | -0.2515 | 1.34E-06 | 0.05194 | -0.028 | 0.0357 | 0.4325 |
|  | rs12874404 | G | A | 0.1714 | 1.39E-10 | 0.02663 | 0.0096 | 0.0504 | 0.8484 |
|  | rs144625305 | T | C | 0.6141 | 6.90E-06 | 0.1364 | -0.0171 | 0.0809 | 0.8324 |
|  | rs149701635 | G | C | -0.3548 | 5.08E-06 | 0.07767 | 0.1891 | 0.1068 | 0.076671 |
|  | rs149731447 | T | C | -0.9621 | 8.36E-06 | 0.2156 | -0.2838 | 0.1145 | 0.01315 |
|  | rs2003291 | T | C | -0.1589 | 8.77E-06 | 0.0357 | 0.0063 | 0.0224 | 0.779701 |
|  | rs28594458 | G | C | -0.1135 | 2.77E-06 | 0.02418 | -0.0028 | 0.0186 | 0.8821 |
|  | rs35088452 | A | C | -0.1352 | 2.16E-06 | 0.02849 | -0.0068 | 0.0195 | 0.7279 |
|  | rs62235484 | A | G | 0.4662 | 7.14E-07 | 0.09386 | 0.1209 | 0.0392 | 0.002041 |
|  | rs6895553 | C | T | -0.199 | 3.30E-06 | 0.04272 | -0.0386 | 0.0348 | 0.2679 |
|  | rs72706301 | A | G | 0.448 | 1.98E-06 | 0.09406 | -0.108 | 0.0727 | 0.1375 |
|  | rs77055472 | A | T | -0.2682 | 3.36E-06 | 0.05762 | -0.0394 | 0.0648 | 0.5431 |
|  | rs77762131 | C | G | 0.3532 | 1.25E-09 | 0.058 | 0.1821 | 0.1057 | 0.084959 |
|  | rs78007261 | T | C | -0.3003 | 4.81E-06 | 0.06556 | 0.1555 | 0.121 | 0.1987 |
|  | rs78009119 | G | T | -0.5085 | 2.50E-06 | 0.1078 | 0.0399 | 0.1809 | 0.8255 |
| Memory B cell %lymphocyte | rs10791220 | G | A | -0.1793 | 3656 | 0.03894 | 0.024 | 0.0224 | 0.2823 |
|  | rs11225155 | A | G | 0.1112 | 3656 | 0.0245 | -0.0171 | 0.0192 | 0.372 |
|  | rs11653761 | T | G | -0.1359 | 3656 | 0.02451 | 0.0011 | 0.0186 | 0.9513 |
|  | rs12636848 | G | T | 0.1242 | 3656 | 0.02655 | -0.0059 | 0.0184 | 0.7467 |
|  | rs12763172 | C | T | 0.132 | 3656 | 0.02749 | 0.0061 | 0.0183 | 0.740901 |
|  | rs12874404 | G | A | 0.2221 | 3656 | 0.0262 | 0.0096 | 0.0504 | 0.8484 |
|  | rs143003356 | A | G | -0.5299 | 3656 | 0.1157 | -0.0242 | 0.064 | 0.7053 |
|  | rs144625305 | T | C | 0.6141 | 3656 | 0.1347 | -0.0171 | 0.0809 | 0.8324 |
|  | rs1770731 | T | A | -0.1473 | 3656 | 0.0326 | 0.0367 | 0.03 | 0.2202 |
|  | rs17708396 | A | C | 2.518 | 3656 | 0.5212 | -0.0748 | 0.0382 | 0.05024 |
|  | rs34173062 | A | G | 0.1453 | 3656 | 0.02991 | -0.0085 | 0.0415 | 0.8373 |
|  | rs3810559 | G | T | -0.1132 | 3656 | 0.02457 | -0.0155 | 0.0188 | 0.4103 |
|  | rs410173 | G | A | -0.144 | 3656 | 0.03208 | -0.033 | 0.0207 | 0.1112 |
|  | rs4640493 | T | A | 0.1493 | 3656 | 0.03234 | 0.0088 | 0.0262 | 0.7359 |
|  | rs551529415 | T | C | -0.5751 | 3656 | 0.124 | 0.3107 | 0.4992 | 0.5336 |
|  | rs6558507 | T | C | -0.2635 | 3656 | 0.05776 | 0.0521 | 0.1114 | 0.6401 |
|  | rs6895553 | C | T | -0.1889 | 3656 | 0.04221 | -0.0386 | 0.0348 | 0.2679 |
|  | rs7254039 | A | G | -0.2668 | 3656 | 0.05819 | 0.0032 | 0.0396 | 0.9363 |
|  | rs73192651 | T | C | 0.442 | 3656 | 0.08807 | -0.1404 | 0.0728 | 0.05383 |
|  | rs74839056 | T | C | 1.357 | 3656 | 0.3052 | -0.0825 | 0.064 | 0.1978 |
|  | rs75414548 | A | G | 0.2864 | 3656 | 0.06412 | 0.0277 | 0.0386 | 0.4739 |
|  | rs75766272 | T | C | -0.3971 | 3656 | 0.08543 | 0.0135 | 0.0354 | 0.702999 |
|  | rs77762131 | C | G | 0.3049 | 3656 | 0.05737 | 0.1821 | 0.1057 | 0.084959 |
|  | rs878471 | A | G | -0.1135 | 3656 | 0.02517 | 0.0258 | 0.0188 | 0.1717 |
|  | rs9514859 | C | G | 0.112 | 3656 | 0.02469 | -0.0106 | 0.0196 | 0.5877 |
|  | rs9614226 | G | T | -0.3841 | 3656 | 0.08631 | 0.0362 | 0.0619 | 0.5586 |
|  | rs974368 | A | G | 0.1136 | 3656 | 0.02464 | 0.0105 | 0.0186 | 0.574101 |
| Activated & resting Treg AC | rs11850806 | G | T | -0.6053 | 3.41E-06 | 0.1301 | 0.0134 | 0.0554 | 0.8082 |
|  | rs12049737 | A | G | 0.2633 | 1.55E-06 | 0.05471 | 0.0387 | 0.0329 | 0.2395 |
|  | rs144404462 | A | G | 0.4385 | 3.01E-07 | 0.08542 | -0.0217 | 0.0899 | 0.8097 |
|  | rs146746694 | A | G | 0.5612 | 3.65E-06 | 0.121 | -0.0158 | 0.1066 | 0.8823 |
|  | rs147199078 | C | T | 0.354 | 9.09E-07 | 0.07196 | -0.0157 | 0.1572 | 0.9204 |
|  | rs1819445 | A | G | -0.1624 | 4.32E-06 | 0.03527 | -0.0606 | 0.0266 | 0.02276 |
|  | rs182630194 | A | G | 1.629 | 1.82E-06 | 0.3406 | 0.0292 | 0.1854 | 0.8747 |
|  | rs2108908 | C | A | 0.2204 | 4.85E-07 | 0.04371 | 0.0045 | 0.0209 | 0.8293 |
|  | rs2524084 | G | A | -0.1389 | 8.45E-06 | 0.03113 | -0.0425 | 0.0198 | 0.03174 |
|  | rs408686 | A | C | -0.164 | 4.71E-07 | 0.03249 | 0.0404 | 0.0556 | 0.468 |
|  | rs55732147 | A | G | 0.1242 | 7.98E-06 | 0.02777 | -0.0195 | 0.0278 | 0.4832 |
|  | rs6074425 | A | G | 0.1319 | 3.58E-06 | 0.02841 | -3.00E-04 | 0.0298 | 0.9906 |
|  | rs66762238 | C | T | -0.1109 | 5.73E-06 | 0.0244 | -0.0166 | 0.0183 | 0.364 |
|  | rs6751481 | C | T | 0.1507 | 1.68E-09 | 0.02494 | 0.0394 | 0.0182 | 0.03046 |
|  | rs78409263 | C | G | -0.2043 | 8.71E-08 | 0.03808 | -0.0028 | 0.0257 | 0.9146 |
|  | rs79684619 | G | A | 0.2954 | 6.33E-07 | 0.0592 | 0.0076 | 0.0494 | 0.8777 |
|  | rs8064831 | T | C | -0.1151 | 5.03E-06 | 0.02518 | 0.0105 | 0.0227 | 0.6427 |
|  | rs9272226 | T | C | 0.1634 | 3.67E-07 | 0.03206 | 0.0478 | 0.0195 | 0.01436 |
|  | rs9709738 | C | G | -0.1302 | 2.54E-06 | 0.02762 | 0.0234 | 0.0201 | 0.245 |
| Activated & secreting Treg %CD4+ | rs10925924 | G | T | -0.1944 | 0.03852 | 4.77E-07 | -0.0202 | 0.0279 | 0.4684 |
|  | rs11221698 | T | C | -0.1382 | 0.0306 | 6.53E-06 | 6.00E-04 | 0.0273 | 0.9826 |
|  | rs11256966 | G | C | -0.1567 | 0.02626 | 2.64E-09 | -0.0206 | 0.0244 | 0.3989 |
|  | rs113435341 | G | A | -2.11 | 0.2195 | 1.31E-21 | -0.1399 | 0.1346 | 0.2985 |
|  | rs114437347 | C | G | -3.73 | 0.8097 | 4.23E-06 | 0.0638 | 0.1603 | 0.690799 |
|  | rs11579717 | A | G | -1.573 | 0.1855 | 3.35E-17 | -0.0033 | 0.0347 | 0.9245 |
|  | rs11655776 | G | A | 0.2327 | 0.05214 | 8.35E-06 | -0.0101 | 0.0359 | 0.777301 |
|  | rs117947776 | C | G | -2.446 | 0.5459 | 7.65E-06 | 0.169 | 0.0856 | 0.04842 |
|  | rs12642085 | T | C | -0.1149 | 0.02598 | 9.94E-06 | 0.0024 | 0.0184 | 0.8952 |
|  | rs12712610 | G | A | -0.1572 | 0.02752 | 1.21E-08 | -0.0191 | 0.028 | 0.4955 |
|  | rs138915779 | A | C | -1.565 | 0.2272 | 6.75E-12 | 0.0703 | 0.075 | 0.3486 |
|  | rs138960714 | C | A | -2.923 | 0.4989 | 5.13E-09 | 7.00E-04 | 0.1128 | 0.9949 |
|  | rs140078626 | G | C | 0.2033 | 0.04548 | 8.13E-06 | -0.0811 | 0.0568 | 0.1538 |
|  | rs143166038 | A | G | 1.332 | 0.2918 | 5.15E-06 | -0.021 | 0.0463 | 0.6502 |
|  | rs143290109 | T | C | -2.089 | 0.4694 | 8.89E-06 | -0.0246 | 0.1227 | 0.8414 |
|  | rs145444215 | A | G | -1.986 | 0.4154 | 1.83E-06 | 0.0846 | 0.1094 | 0.439 |
|  | rs146512168 | C | T | -2.494 | 0.5458 | 5.09E-06 | 0.0533 | 0.0787 | 0.4987 |
|  | rs1465510 | A | G | -0.1198 | 0.0267 | 7.43E-06 | 0.0361 | 0.0192 | 0.060959 |
|  | rs147838148 | G | T | -2.662 | 0.4776 | 2.68E-08 | 0.1223 | 0.0655 | 0.06198 |
|  | rs149703905 | G | A | -2.484 | 0.5461 | 5.59E-06 | 0.2483 | 0.09 | 0.005776 |
|  | rs17465223 | A | G | -0.1728 | 0.03682 | 2.81E-06 | -0.0092 | 0.0237 | 0.696599 |
|  | rs186715000 | G | A | -2.136 | 0.466 | 4.75E-06 | 0.0325 | 0.1973 | 0.869 |
|  | rs2668533 | T | C | -0.131 | 0.02803 | 3.09E-06 | 0.036 | 0.0182 | 0.04836 |
|  | rs2958568 | C | A | 0.1207 | 0.02532 | 1.94E-06 | 0.0068 | 0.0183 | 0.709999 |
|  | rs35378667 | C | T | -0.5302 | 0.1029 | 2.72E-07 | -0.0099 | 0.0722 | 0.8906 |
|  | rs4685759 | G | C | -0.11 | 0.02458 | 7.85E-06 | -0.0466 | 0.0182 | 0.0106 |
|  | rs4901222 | T | C | -2.838 | 0.5947 | 1.89E-06 | 0.0784 | 0.036 | 0.02966 |
|  | rs6065288 | T | C | 0.1447 | 0.03148 | 4.43E-06 | 0.0113 | 0.0267 | 0.671399 |
|  | rs61839660 | T | C | 0.4097 | 0.05516 | 1.38E-13 | 0.0077 | 0.046 | 0.8676 |
|  | rs6438325 | C | A | -0.1805 | 0.03716 | 1.23E-06 | 0.007 | 0.0315 | 0.8232 |
|  | rs73155096 | G | C | -0.7413 | 0.1601 | 3.77E-06 | -0.0136 | 0.0434 | 0.753 |
|  | rs74830391 | T | C | 0.1992 | 0.03616 | 3.87E-08 | -0.0144 | 0.0279 | 0.6059 |
| CD25hi CD45RA+ CD4 not Treg %CD4+ | rs10905719 | A | G | 0.1841 | 7.06E-09 | 0.03172 | 0.0027 | 0.019 | 0.8874 |
|  | rs11124653 | A | C | -0.2138 | 5.23E-16 | 0.02624 | -0.0134 | 0.0192 | 0.486 |
|  | rs11195783 | A | G | -0.1482 | 5.12E-06 | 0.03245 | -0.0221 | 0.0332 | 0.5058 |
|  | rs114437347 | C | G | 3.89 | 4.33E-06 | 0.8453 | 0.0638 | 0.1603 | 0.690799 |
|  | rs114780506 | T | A | 3.688 | 2.81E-06 | 0.7859 | 0.1667 | 0.1392 | 0.2311 |
|  | rs116517602 | T | C | 1.941 | 4.76E-24 | 0.1905 | 0.1051 | 0.0939 | 0.2631 |
|  | rs116850494 | A | G | 0.4908 | 5.00E-06 | 0.1074 | 0.0453 | 0.0439 | 0.3023 |
|  | rs117626397 | G | A | 3.007 | 3.10E-07 | 0.5864 | 0.1619 | 0.0774 | 0.03643 |
|  | rs1264473 | T | C | 0.1141 | 9.69E-06 | 0.02576 | -8.00E-04 | 0.0184 | 0.9672 |
|  | rs12958644 | C | A | 0.2701 | 4.26E-07 | 0.05331 | 0.066 | 0.039 | 0.09105 |
|  | rs13024840 | A | T | 0.2764 | 4.05E-21 | 0.02911 | -0.013 | 0.0318 | 0.683501 |
|  | rs13260343 | G | A | -0.1147 | 7.00E-06 | 0.0255 | -0.0017 | 0.0183 | 0.9282 |
|  | rs141541045 | T | A | 0.7445 | 2.28E-06 | 0.1572 | -0.0215 | 0.0617 | 0.7274 |
|  | rs1430018 | C | T | -0.2323 | 1.13E-07 | 0.04369 | -0.0037 | 0.0255 | 0.8851 |
|  | rs149429639 | C | T | 1.783 | 1.96E-06 | 0.3741 | 0.0097 | 0.0651 | 0.8821 |
|  | rs149541387 | A | T | 0.4045 | 4.52E-06 | 0.08806 | -0.1917 | 0.1546 | 0.215 |
|  | rs150635171 | T | C | 0.4488 | 7.43E-06 | 0.1 | -0.0099 | 0.0464 | 0.8315 |
|  | rs1645894 | C | G | -0.1536 | 7.77E-06 | 0.03429 | 0.0274 | 0.0244 | 0.2613 |
|  | rs17022951 | A | G | 0.342 | 2.35E-06 | 0.07231 | -0.0955 | 0.0735 | 0.1935 |
|  | rs180839256 | C | T | 0.7622 | 1.38E-07 | 0.1444 | -0.0224 | 0.1031 | 0.8283 |
|  | rs182812626 | G | C | 1.067 | 2.33E-07 | 0.206 | 0.0329 | 0.0853 | 0.6997 |
|  | rs188418491 | G | A | 2.218 | 4.88E-06 | 0.4847 | 0.0043 | 0.053 | 0.936 |
|  | rs274814 | C | T | 0.1936 | 8.76E-06 | 0.04348 | 0.0499 | 0.0207 | 0.01605 |
|  | rs28446907 | G | A | -0.7343 | 5.26E-06 | 0.161 | 0.0122 | 0.0711 | 0.8634 |
|  | rs56000618 | T | G | 0.1237 | 7.92E-06 | 0.02764 | 0.0262 | 0.0241 | 0.2769 |
|  | rs61836370 | G | A | 0.6318 | 4.42E-09 | 0.1074 | 0.0021 | 0.0299 | 0.9443 |
|  | rs73329062 | A | G | -0.6809 | 6.70E-06 | 0.151 | -0.0914 | 0.0986 | 0.3539 |
|  | rs73579441 | C | T | 0.225 | 8.33E-06 | 0.05042 | 9.00E-04 | 0.0521 | 0.9864 |
|  | rs738595 | C | T | 0.1331 | 7.11E-06 | 0.02959 | -0.0152 | 0.0233 | 0.5136 |
|  | rs75798204 | T | C | -0.8787 | 9.15E-06 | 0.1978 | -0.069 | 0.0574 | 0.2297 |
|  | rs75958070 | C | G | -0.1771 | 6.58E-06 | 0.03924 | -0.0015 | 0.0258 | 0.9534 |
|  | rs77180728 | C | G | -0.1628 | 8.32E-06 | 0.03648 | -0.0525 | 0.0543 | 0.3338 |
|  | rs79536602 | G | T | 0.4559 | 2.32E-06 | 0.09637 | 0.0304 | 0.0493 | 0.5372 |
| CD25hi CD45RA+ CD4 not Treg %T cell | rs10905719 | A | G | 0.1734 | 4.88E-08 | 0.03172 | 0.0027 | 0.019 | 0.8874 |
|  | rs11124653 | A | C | -0.1952 | 1.36E-13 | 0.02627 | -0.0134 | 0.0192 | 0.486 |
|  | rs112238613 | C | T | 1.473 | 9.58E-06 | 0.3324 | 0.0789 | 0.056 | 0.159 |
|  | rs114437347 | C | G | 3.798 | 7.19E-06 | 0.8449 | 0.0638 | 0.1603 | 0.690799 |
|  | rs114780506 | T | A | 3.735 | 2.05E-06 | 0.7854 | 0.1667 | 0.1392 | 0.2311 |
|  | rs116517602 | T | C | 1.875 | 1.50E-22 | 0.1906 | 0.1051 | 0.0939 | 0.2631 |
|  | rs117520868 | A | C | 0.332 | 4.41E-06 | 0.0722 | -0.0047 | 0.0472 | 0.9208 |
|  | rs117626397 | G | A | 2.958 | 4.72E-07 | 0.5861 | 0.1619 | 0.0774 | 0.03643 |
|  | rs117882514 | C | A | 0.3547 | 6.17E-07 | 0.07102 | 0.0746 | 0.0593 | 0.2086 |
|  | rs1264473 | T | C | 0.1172 | 5.47E-06 | 0.02574 | -8.00E-04 | 0.0184 | 0.9672 |
|  | rs13024840 | A | T | 0.2465 | 4.22E-17 | 0.02917 | -0.013 | 0.0318 | 0.683501 |
|  | rs144105723 | G | A | 0.2382 | 3.74E-06 | 0.05143 | 0.0329 | 0.0834 | 0.693 |
|  | rs144350975 | A | C | 0.4042 | 9.02E-07 | 0.08214 | -0.0612 | 0.0752 | 0.4153 |
|  | rs149429639 | C | T | 1.778 | 2.06E-06 | 0.3739 | 0.0097 | 0.0651 | 0.8821 |
|  | rs17607399 | T | C | 0.1985 | 1.70E-06 | 0.0414 | -0.094 | 0.0336 | 0.005132 |
|  | rs180839256 | C | T | 0.7369 | 3.48E-07 | 0.1443 | -0.0224 | 0.1031 | 0.8283 |
|  | rs188418491 | G | A | 2.205 | 5.53E-06 | 0.4844 | 0.0043 | 0.053 | 0.936 |
|  | rs28446907 | G | A | -0.7381 | 4.64E-06 | 0.1609 | 0.0122 | 0.0711 | 0.8634 |
|  | rs4876568 | T | G | -0.4787 | 2.96E-06 | 0.1023 | -0.0293 | 0.0405 | 0.4701 |
|  | rs61836370 | G | A | 0.6084 | 1.58E-08 | 0.1074 | 0.0021 | 0.0299 | 0.9443 |
|  | rs67017865 | A | T | 0.1911 | 8.77E-06 | 0.04292 | 0.0282 | 0.0303 | 0.353 |
|  | rs72797312 | T | A | -0.1472 | 8.32E-06 | 0.03298 | -0.0096 | 0.0294 | 0.7428 |
|  | rs75798204 | T | C | -0.8751 | 9.83E-06 | 0.1977 | -0.069 | 0.0574 | 0.2297 |
|  | rs75958070 | C | G | -0.1904 | 1.24E-06 | 0.03919 | -0.0015 | 0.0258 | 0.9534 |
|  | rs76432001 | A | C | -0.3904 | 9.80E-06 | 0.08817 | -0.0932 | 0.0896 | 0.2985 |
|  | rs78032501 | C | G | -0.7964 | 7.28E-06 | 0.1773 | 0.1701 | 0.0739 | 0.0213 |
|  | rs79536602 | G | T | 0.4553 | 2.36E-06 | 0.09631 | 0.0304 | 0.0493 | 0.5372 |
| CD45RA- CD4+ AC | rs11062774 | G | A | 0.1756 | 9.68E-07 | 0.03579 | -0.0315 | 0.033 | 0.3403 |
|  | rs117563350 | G | A | -0.3399 | 4.49E-06 | 0.07398 | 0.0127 | 0.1302 | 0.9225 |
|  | rs137903114 | C | T | -0.9595 | 7.47E-06 | 0.2138 | 0.0936 | 0.0821 | 0.2544 |
|  | rs145638437 | A | G | 0.7099 | 7.78E-06 | 0.1585 | -0.0178 | 0.0654 | 0.785199 |
|  | rs148716007 | T | C | 0.2941 | 3.97E-06 | 0.06366 | 0.1322 | 0.0476 | 0.005523 |
|  | rs150956189 | C | T | -2.99 | 6.48E-06 | 0.6619 | -0.0805 | 0.0471 | 0.087171 |
|  | rs41306808 | G | C | 0.2331 | 1.99E-06 | 0.04895 | 0.0262 | 0.0362 | 0.4689 |
|  | rs4357954 | G | C | -0.2354 | 5.98E-07 | 0.04706 | -0.0103 | 0.0342 | 0.763001 |
|  | rs541367304 | A | G | -1.857 | 4.24E-12 | 0.267 | -0.1433 | 0.1366 | 0.2943 |
|  | rs55905714 | C | A | 0.1712 | 4.93E-06 | 0.03741 | 0.0566 | 0.038 | 0.1364 |
|  | rs62447160 | C | T | -0.138 | 3.51E-07 | 0.02703 | 0.0026 | 0.0192 | 0.8916 |
|  | rs653178 | T | C | -0.1619 | 1.18E-08 | 0.02833 | 0.0143 | 0.0184 | 0.4392 |
|  | rs6743943 | C | T | 0.1354 | 3.57E-07 | 0.02655 | 0.0255 | 0.0183 | 0.1637 |
|  | rs7162344 | A | G | -0.1295 | 6.29E-07 | 0.02595 | -0.0243 | 0.02 | 0.2249 |
|  | rs73176863 | T | G | 2.335 | 9.82E-06 | 0.5273 | 0.0756 | 0.0925 | 0.4138 |
|  | rs9961909 | C | T | -0.3058 | 1.74E-06 | 0.06385 | 0.0064 | 0.0226 | 0.7754 |
| CD45RA- CD4+ %CD4+ | rs10013469 | A | G | 0.192 | 0.04023 | 1.89E-06 | 0.0098 | 0.0454 | 0.8299 |
|  | rs10230422 | A | G | 0.1892 | 0.04192 | 6.61E-06 | -0.0249 | 0.0323 | 0.4402 |
|  | rs10432663 | C | T | -0.1402 | 0.03003 | 3.14E-06 | -0.0478 | 0.0224 | 0.03326 |
|  | rs11124653 | A | C | 0.1345 | 0.02443 | 3.92E-08 | -0.0134 | 0.0192 | 0.486 |
|  | rs114825362 | C | G | -0.3913 | 0.08534 | 4.70E-06 | 0.0432 | 0.1157 | 0.7092 |
|  | rs11579717 | A | G | -1.081 | 0.1759 | 8.87E-10 | -0.0033 | 0.0347 | 0.9245 |
|  | rs116794175 | C | T | -0.8093 | 0.1824 | 9.42E-06 | -0.2482 | 0.2497 | 0.3203 |
|  | rs12121018 | A | G | -0.1566 | 0.03132 | 6.00E-07 | 0.0173 | 0.039 | 0.6575 |
|  | rs1250600 | G | T | -0.1145 | 0.02553 | 7.58E-06 | -0.0352 | 0.0196 | 0.07238 |
|  | rs12712610 | G | A | -0.1902 | 0.02599 | 3.13E-13 | -0.0191 | 0.028 | 0.4955 |
|  | rs141350785 | C | G | -0.2543 | 0.0556 | 4.95E-06 | -0.017 | 0.0288 | 0.5535 |
|  | rs146329634 | T | C | 0.3128 | 0.06543 | 1.82E-06 | -0.0289 | 0.0713 | 0.6848 |
|  | rs146620586 | T | G | -2.021 | 0.2655 | 3.46E-14 | -0.058 | 0.1248 | 0.642 |
|  | rs146674398 | T | C | -0.4158 | 0.09372 | 9.44E-06 | -0.0387 | 0.041 | 0.3445 |
|  | rs150607181 | C | G | -0.4267 | 0.09412 | 6.01E-06 | -0.06 | 0.0721 | 0.405 |
|  | rs183293285 | T | C | -0.425 | 0.09182 | 3.81E-06 | -0.0733 | 0.0671 | 0.2741 |
|  | rs1849779 | T | C | -0.1119 | 0.02448 | 5.04E-06 | -0.0147 | 0.0188 | 0.4331 |
|  | rs2252009 | G | T | 0.1373 | 0.03055 | 7.22E-06 | 0.0244 | 0.02 | 0.2231 |
|  | rs62341785 | G | A | 0.1329 | 0.02929 | 5.94E-06 | 0.0012 | 0.0251 | 0.9615 |
|  | rs71569312 | T | C | 0.4277 | 0.09186 | 3.35E-06 | 0.0384 | 0.0603 | 0.5248 |
|  | rs7216330 | T | C | -0.1152 | 0.02407 | 1.77E-06 | 0.005 | 0.0187 | 0.790801 |
|  | rs72613709 | G | T | 0.2888 | 0.06249 | 3.94E-06 | -0.0054 | 0.0317 | 0.8653 |
|  | rs73189225 | A | G | -0.1661 | 0.03716 | 8.12E-06 | 0.0103 | 0.0198 | 0.6052 |
|  | rs76139558 | C | G | 0.6888 | 0.1394 | 8.18E-07 | 0.0307 | 0.172 | 0.8585 |
|  | rs76201922 | G | C | -0.3839 | 0.08517 | 6.77E-06 | -0.016 | 0.047 | 0.734199 |
|  | rs76516339 | T | A | -0.1648 | 0.03213 | 3.05E-07 | -0.0385 | 0.0207 | 0.06315 |
|  | rs7700617 | A | C | -0.1075 | 0.02387 | 6.87E-06 | -0.009 | 0.0188 | 0.631801 |
|  | rs9271766 | G | A | -0.1619 | 0.03361 | 1.51E-06 | -0.0451 | 0.0215 | 0.03611 |
|  | rs931555 | T | C | -0.1246 | 0.02807 | 9.25E-06 | -0.0012 | 0.0187 | 0.9476 |
| EM CD4+ AC | rs112785791 | A | G | -0.3605 | 9.53E-06 | 0.0813 | 0.0453 | 0.0421 | 0.2812 |
|  | rs115531274 | G | A | 0.1635 | 7.27E-06 | 0.03639 | -0.0543 | 0.0333 | 0.1028 |
|  | rs115795061 | A | T | -0.3061 | 8.37E-06 | 0.06859 | 0.0675 | 0.0996 | 0.4983 |
|  | rs11579717 | A | G | -0.8572 | 8.25E-06 | 0.192 | -0.0033 | 0.0347 | 0.9245 |
|  | rs117563350 | G | A | -0.3637 | 6.56E-07 | 0.07298 | 0.0127 | 0.1302 | 0.9225 |
|  | rs12894769 | A | G | -0.3375 | 9.33E-06 | 0.07604 | 0.0464 | 0.0428 | 0.279 |
|  | rs142002778 | C | T | -0.4619 | 5.93E-06 | 0.1018 | -0.0407 | 0.0699 | 0.5604 |
|  | rs143648086 | A | G | -0.3431 | 7.96E-06 | 0.0767 | 0.0174 | 0.1539 | 0.9098 |
|  | rs148709760 | A | G | -0.3035 | 2.36E-06 | 0.0642 | -0.087 | 0.0662 | 0.1887 |
|  | rs150956189 | C | T | -3.037 | 3.45E-06 | 0.6532 | -0.0805 | 0.0471 | 0.087171 |
|  | rs17183358 | A | T | -0.241 | 9.55E-06 | 0.05435 | 0.0052 | 0.0228 | 0.818 |
|  | rs17376950 | C | G | -0.3012 | 1.16E-06 | 0.06185 | -0.0306 | 0.0416 | 0.4615 |
|  | rs541367304 | A | G | -1.792 | 1.25E-11 | 0.2636 | -0.1433 | 0.1366 | 0.2943 |
|  | rs62447160 | C | T | -0.1312 | 9.34E-07 | 0.02669 | 0.0026 | 0.0192 | 0.8916 |
|  | rs6729741 | T | A | -0.1485 | 1.24E-08 | 0.02603 | 0.0063 | 0.0184 | 0.732799 |
|  | rs72705882 | T | C | -0.1714 | 7.12E-06 | 0.03812 | -0.0697 | 0.0319 | 0.02886 |
|  | rs74010649 | T | G | -0.2226 | 3.90E-06 | 0.04815 | -0.0025 | 0.0348 | 0.943 |
|  | rs74443612 | G | A | -0.5672 | 4.78E-06 | 0.1238 | -0.0919 | 0.0433 | 0.03355 |
|  | rs75275482 | T | C | -2.355 | 6.21E-06 | 0.5203 | -0.2618 | 0.1926 | 0.1741 |
|  | rs75603168 | T | C | -0.6847 | 7.33E-06 | 0.1525 | -0.0216 | 0.0602 | 0.719201 |
|  | rs7737130 | A | C | 0.2305 | 3.24E-06 | 0.04943 | -0.0579 | 0.0339 | 0.08744 |
|  | rs77802035 | T | C | 0.3907 | 8.32E-06 | 0.08754 | 3.00E-04 | 0.084 | 0.997 |
|  | rs78082758 | G | A | -0.3978 | 1.27E-06 | 0.08196 | -0.0316 | 0.0606 | 0.6016 |
|  | rs9961909 | C | T | -0.3209 | 3.68E-07 | 0.06299 | 0.0064 | 0.0226 | 0.7754 |
|  | rs9967156 | C | G | 0.1203 | 3.07E-06 | 0.02573 | 0.0054 | 0.0191 | 0.7784 |
| CD45RA+ CD8br AC | rs11110042 | T | C | 0.1248 | 0.02717 | 4.52E-06 | 0.0055 | 0.0201 | 0.7863 |
|  | rs114799586 | T | C | 0.2664 | 0.05548 | 1.64E-06 | 0.1863 | 0.1155 | 0.1068 |
|  | rs114968045 | C | T | -0.1586 | 0.02793 | 1.48E-08 | 0.0101 | 0.0394 | 0.7971 |
|  | rs117284243 | T | A | 3.818 | 0.8406 | 5.77E-06 | -0.1037 | 0.0605 | 0.08673 |
|  | rs118124576 | G | T | -0.6898 | 0.149 | 3.81E-06 | 0.0013 | 0.0676 | 0.9844 |
|  | rs11896593 | C | G | 0.197 | 0.02252 | 3.31E-18 | -0.0166 | 0.0271 | 0.5387 |
|  | rs13381634 | G | A | 0.0983 | 0.02175 | 6.44E-06 | 2.00E-04 | 0.021 | 0.9932 |
|  | rs138746551 | G | A | 0.3584 | 0.08052 | 8.84E-06 | -0.0042 | 0.0409 | 0.918 |
|  | rs140940501 | A | G | -0.616 | 0.1358 | 5.91E-06 | -0.0206 | 0.0418 | 0.6226 |
|  | rs1550856 | G | A | -0.1222 | 0.02651 | 4.16E-06 | -0.0704 | 0.0257 | 0.00612 |
|  | rs16968252 | A | G | 0.4151 | 0.09362 | 9.53E-06 | 0.1074 | 0.1397 | 0.4417 |
|  | rs17655711 | C | T | -0.2433 | 0.05486 | 9.47E-06 | -0.0576 | 0.066 | 0.383 |
|  | rs185587352 | A | G | 1.982 | 0.4431 | 8.00E-06 | -0.1964 | 0.0951 | 0.03894 |
|  | rs2837838 | G | A | 0.1995 | 0.04112 | 1.28E-06 | -0.0413 | 0.0403 | 0.3046 |
|  | rs28578699 | C | A | 0.1216 | 0.02273 | 9.48E-08 | 0.0281 | 0.0183 | 0.1245 |
|  | rs3104369 | C | T | 0.1747 | 0.02866 | 1.22E-09 | -0.052 | 0.0201 | 0.009505 |
|  | rs35644926 | C | T | 0.1782 | 0.03967 | 7.26E-06 | 0.016 | 0.0269 | 0.5525 |
|  | rs62077212 | C | T | 0.1285 | 0.02492 | 2.65E-07 | 0.0267 | 0.0218 | 0.2209 |
|  | rs62321187 | T | C | 3.706 | 0.8273 | 7.70E-06 | 0.0058 | 0.1 | 0.9537 |
|  | rs6943221 | A | T | 0.1253 | 0.02717 | 4.11E-06 | -0.0628 | 0.028 | 0.0252 |
|  | rs72791631 | A | T | -0.1561 | 0.03447 | 6.13E-06 | -0.0225 | 0.0268 | 0.4001 |
|  | rs72826622 | T | C | -0.1555 | 0.03443 | 6.51E-06 | 0.0022 | 0.0337 | 0.9483 |
|  | rs72933273 | T | C | -0.4537 | 0.1023 | 9.41E-06 | -0.0197 | 0.0729 | 0.7866 |
|  | rs75402344 | T | G | 3.282 | 0.6946 | 2.40E-06 | 0.0403 | 0.0455 | 0.3757 |
|  | rs76444609 | A | G | 3.806 | 0.8351 | 5.34E-06 | -0.044 | 0.0589 | 0.4552 |
|  | rs76552842 | A | G | 4.001 | 0.8064 | 7.32E-07 | -0.1001 | 0.0831 | 0.2283 |
|  | rs78750136 | C | T | 7.755 | 1.682 | 4.16E-06 | -0.1154 | 0.0551 | 0.03638 |
|  | rs79777035 | C | T | 0.9887 | 0.198 | 6.22E-07 | 0.0629 | 0.1154 | 0.5857 |
| EM DN (CD4-CD8-) %T cell | rs10225877 | T | A | -0.1278 | 1.55E-06 | 0.02656 | 0.0449 | 0.0254 | 0.07674 |
|  | rs11180778 | C | T | 0.1213 | 8.31E-06 | 0.02717 | -0.0548 | 0.0201 | 0.00633 |
|  | rs111902980 | A | G | 0.4328 | 8.35E-07 | 0.08768 | 0.0038 | 0.0363 | 0.9175 |
|  | rs113075410 | G | A | 0.2447 | 7.79E-06 | 0.05465 | 0.0081 | 0.1222 | 0.947 |
|  | rs113500908 | T | C | 0.3476 | 2.83E-09 | 0.05835 | -0.1131 | 0.0403 | 0.004997 |
|  | rs116230798 | T | C | -0.2684 | 2.40E-06 | 0.05681 | 0.0228 | 0.0918 | 0.8035 |
|  | rs11635763 | G | A | 0.1159 | 3.09E-06 | 0.02481 | -0.0333 | 0.0204 | 0.1026 |
|  | rs116877116 | A | G | 0.1973 | 1.09E-06 | 0.04041 | -0.1173 | 0.0681 | 0.085271 |
|  | rs13210358 | T | C | 0.4707 | 4.24E-11 | 0.07114 | -0.1505 | 0.0726 | 0.03816 |
|  | rs140247402 | T | C | -0.2519 | 5.68E-06 | 0.05542 | 0.0465 | 0.0244 | 0.05658 |
|  | rs141519401 | T | G | 0.1737 | 9.22E-06 | 0.03911 | -0.0052 | 0.0761 | 0.9452 |
|  | rs28555522 | G | A | 0.1258 | 1.78E-06 | 0.02629 | -0.0263 | 0.0224 | 0.24 |
|  | rs28647367 | A | G | 0.2288 | 8.41E-06 | 0.05128 | -0.2526 | 0.1619 | 0.1188 |
|  | rs34546581 | A | G | 0.2019 | 5.06E-11 | 0.03063 | -0.0214 | 0.0233 | 0.3586 |
|  | rs437936 | G | T | -0.1625 | 5.43E-06 | 0.03568 | 0.0194 | 0.0235 | 0.4093 |
|  | rs4583837 | T | C | 0.2578 | 3.58E-06 | 0.05553 | -0.0029 | 0.0229 | 0.8998 |
|  | rs527345021 | C | T | -0.8611 | 2.78E-06 | 0.1834 | 0.0358 | 0.0593 | 0.5466 |
|  | rs541367304 | A | G | -1.24 | 9.05E-07 | 0.252 | -0.1433 | 0.1366 | 0.2943 |
|  | rs55781756 | A | G | 0.1501 | 3.52E-06 | 0.0323 | -0.0137 | 0.0326 | 0.6736 |
|  | rs75679242 | A | C | -0.1752 | 3.79E-06 | 0.03783 | 0.0055 | 0.023 | 0.8119 |
|  | rs76747637 | A | G | 0.6931 | 4.57E-06 | 0.151 | 0.0055 | 0.0407 | 0.8928 |
|  | rs77784181 | A | G | 1.494 | 9.40E-06 | 0.3368 | -0.1239 | 0.1036 | 0.2318 |
|  | rs78118522 | C | A | -0.1278 | 3.66E-06 | 0.02756 | 0.0128 | 0.0183 | 0.4857 |
|  | rs78522661 | T | C | 0.1964 | 1.11E-06 | 0.04026 | 0.0438 | 0.0449 | 0.3296 |
|  | rs9896155 | G | A | 0.1315 | 3.91E-07 | 0.02587 | 0.0267 | 0.0185 | 0.1484 |
| CD14+ CD16- monocyte %monocyte | rs10833398 | C | T | 0.132 | 0.02957 | 8.30E-06 | -0.0513 | 0.0264 | 0.05254 |
|  | rs111690368 | T | C | -0.7379 | 0.1589 | 3.54E-06 | 0.0582 | 0.1717 | 0.734401 |
|  | rs115383270 | A | G | 0.5518 | 0.03171 | 3.21E-65 | -0.0456 | 0.0387 | 0.2393 |
|  | rs116915583 | T | C | -2.112 | 0.4662 | 6.10E-06 | -0.0948 | 0.1425 | 0.5058 |
|  | rs12637779 | G | A | 0.1521 | 0.03338 | 5.37E-06 | 0.0044 | 0.0259 | 0.8646 |
|  | rs13183256 | A | G | 0.172 | 0.0372 | 3.90E-06 | -0.0052 | 0.0246 | 0.8322 |
|  | rs13330943 | C | T | 0.122 | 0.02599 | 2.81E-06 | 0.0043 | 0.0186 | 0.8172 |
|  | rs137976321 | A | G | -0.4021 | 0.08741 | 4.35E-06 | -0.1694 | 0.0921 | 0.065801 |
|  | rs138743668 | A | G | 0.6107 | 0.1355 | 6.83E-06 | 0.0517 | 0.0705 | 0.4634 |
|  | rs139379948 | A | G | 0.5387 | 0.115 | 2.89E-06 | -0.0472 | 0.0483 | 0.3285 |
|  | rs141826566 | T | C | -2.132 | 0.4804 | 9.34E-06 | 0.0467 | 0.1787 | 0.793699 |
|  | rs142340414 | T | C | -3.05 | 0.6547 | 3.29E-06 | 0.0686 | 0.0401 | 0.08738 |
|  | rs143984211 | T | G | -0.3205 | 0.06928 | 3.85E-06 | -0.0469 | 0.0887 | 0.5972 |
|  | rs149602828 | A | G | 0.3065 | 0.06864 | 8.25E-06 | -0.0667 | 0.0775 | 0.3891 |
|  | rs150125023 | A | G | -0.3023 | 0.06636 | 5.41E-06 | -0.0861 | 0.0531 | 0.1049 |
|  | rs155144 | A | G | -0.1135 | 0.02438 | 3.36E-06 | 0.0222 | 0.0195 | 0.255 |
|  | rs4656993 | G | A | -0.1142 | 0.02492 | 4.71E-06 | 0.0231 | 0.0183 | 0.2072 |
|  | rs4783204 | C | T | -0.1438 | 0.03117 | 4.10E-06 | 0.0324 | 0.0202 | 0.1092 |
|  | rs56347556 | T | A | -0.1892 | 0.04165 | 5.74E-06 | 0.0198 | 0.0568 | 0.728201 |
|  | rs62083033 | C | A | -1.287 | 0.2814 | 4.99E-06 | -0.0252 | 0.1132 | 0.8237 |
|  | rs659192 | G | C | -0.166 | 0.0345 | 1.55E-06 | 0.019 | 0.0255 | 0.4566 |
|  | rs67760360 | A | G | 0.1575 | 0.02992 | 1.49E-07 | -0.0422 | 0.022 | 0.054541 |
|  | rs74341264 | A | G | -0.2169 | 0.03712 | 5.60E-09 | 0.0247 | 0.0314 | 0.4307 |
|  | rs76293503 | A | G | -1.209 | 0.2647 | 5.09E-06 | -0.0047 | 0.12 | 0.9689 |
|  | rs78047442 | T | G | 0.1458 | 0.0328 | 9.00E-06 | -0.0184 | 0.0269 | 0.4947 |
|  | rs79562353 | C | T | 0.2405 | 0.05147 | 3.08E-06 | 0.0597 | 0.0788 | 0.4487 |
| CD16+ monocyte %monocyte | rs10765883 | T | C | 0.1122 | 0.025 | 7.43E-06 | 0.0257 | 0.0189 | 0.1733 |
|  | rs111690368 | T | C | 0.7119 | 0.159 | 7.76E-06 | 0.0582 | 0.1717 | 0.734401 |
|  | rs115383270 | A | G | -0.5431 | 0.03176 | 4.06E-63 | -0.0456 | 0.0387 | 0.2393 |
|  | rs115440084 | T | C | 0.3715 | 0.0834 | 8.67E-06 | 0.0756 | 0.0915 | 0.4087 |
|  | rs12582907 | C | T | -0.1424 | 0.03159 | 6.82E-06 | -0.0019 | 0.0212 | 0.9294 |
|  | rs12637779 | G | A | -0.1513 | 0.03339 | 6.06E-06 | 0.0044 | 0.0259 | 0.8646 |
|  | rs13183256 | A | G | -0.1714 | 0.03722 | 4.24E-06 | -0.0052 | 0.0246 | 0.8322 |
|  | rs13330943 | C | T | -0.1248 | 0.026 | 1.64E-06 | 0.0043 | 0.0186 | 0.8172 |
|  | rs137976321 | A | G | 0.3913 | 0.08745 | 7.91E-06 | -0.1694 | 0.0921 | 0.065801 |
|  | rs138743668 | A | G | -0.6208 | 0.1356 | 4.83E-06 | 0.0517 | 0.0705 | 0.4634 |
|  | rs139379948 | A | G | -0.5341 | 0.115 | 3.54E-06 | -0.0472 | 0.0483 | 0.3285 |
|  | rs143356597 | A | G | 0.2916 | 0.06193 | 2.59E-06 | -0.0792 | 0.0764 | 0.3003 |
|  | rs144707945 | C | G | -1.095 | 0.2433 | 6.96E-06 | 0.0035 | 0.1063 | 0.9739 |
|  | rs150125023 | A | G | 0.302 | 0.06638 | 5.56E-06 | -0.0861 | 0.0531 | 0.1049 |
|  | rs150451378 | A | C | -0.4106 | 0.09264 | 9.57E-06 | 0.0589 | 0.1611 | 0.7145 |
|  | rs155144 | A | G | 0.1127 | 0.02439 | 3.97E-06 | 0.0222 | 0.0195 | 0.255 |
|  | rs4656993 | G | A | 0.1155 | 0.02492 | 3.68E-06 | 0.0231 | 0.0183 | 0.2072 |
|  | rs4783204 | C | T | 0.1482 | 0.03118 | 2.07E-06 | 0.0324 | 0.0202 | 0.1092 |
|  | rs659192 | G | C | 0.1659 | 0.03451 | 1.59E-06 | 0.019 | 0.0255 | 0.4566 |
|  | rs67760360 | A | G | -0.1632 | 0.02992 | 5.21E-08 | -0.0422 | 0.022 | 0.054541 |
|  | rs72982284 | T | C | 0.1303 | 0.02919 | 8.30E-06 | 0.0872 | 0.0318 | 0.006172 |
|  | rs74341264 | A | G | 0.2168 | 0.03713 | 5.67E-09 | 0.0247 | 0.0314 | 0.4307 |
|  | rs7613930 | T | C | -0.1264 | 0.02825 | 7.94E-06 | -0.0207 | 0.0234 | 0.3748 |
|  | rs79562353 | C | T | -0.2421 | 0.05148 | 2.68E-06 | 0.0597 | 0.0788 | 0.4487 |
| HLA DR+ CD8br AC | rs10152591 | C | A | -0.2253 | 5.96E-06 | 0.04968 | -0.0042 | 0.0373 | 0.9096 |
|  | rs1084560 | G | A | -0.1331 | 1.06E-07 | 0.02498 | -0.0238 | 0.0198 | 0.2297 |
|  | rs11153123 | G | A | -0.1305 | 8.32E-06 | 0.02924 | 0.0011 | 0.0185 | 0.9524 |
|  | rs11197647 | T | G | 0.2373 | 2.27E-07 | 0.04575 | 0.0017 | 0.028 | 0.9523 |
|  | rs112455120 | T | C | 0.3535 | 7.79E-07 | 0.07143 | -0.0285 | 0.0283 | 0.3129 |
|  | rs117517764 | G | A | 0.1906 | 6.98E-06 | 0.04234 | 0.0036 | 0.0236 | 0.879 |
|  | rs12029484 | G | A | -0.2266 | 9.79E-07 | 0.0462 | -0.0428 | 0.0387 | 0.2687 |
|  | rs138213638 | C | T | 1.495 | 6.69E-06 | 0.3314 | 0.0514 | 0.0439 | 0.2413 |
|  | rs138678763 | C | T | -0.8815 | 2.65E-07 | 0.1709 | 0.2019 | 0.1282 | 0.1153 |
|  | rs17286630 | G | A | -0.3262 | 1.53E-06 | 0.06773 | 0.0213 | 0.063 | 0.736 |
|  | rs2181036 | C | T | -0.1446 | 3.17E-08 | 0.02608 | -0.0096 | 0.0182 | 0.597601 |
|  | rs2516465 | T | G | 0.1607 | 3.43E-07 | 0.03146 | 0.0032 | 0.0205 | 0.8753 |
|  | rs2808067 | C | T | 0.1243 | 2.01E-06 | 0.0261 | 0.0101 | 0.0184 | 0.581599 |
|  | rs3087456 | A | G | -0.4286 | 5.57E-52 | 0.0278 | -0.0097 | 0.0206 | 0.6376 |
|  | rs35669214 | C | T | -0.1242 | 9.05E-06 | 0.02793 | -0.0087 | 0.0216 | 0.6889 |
|  | rs506151 | T | C | -0.4162 | 2.26E-06 | 0.08787 | 0.029 | 0.03 | 0.3325 |
|  | rs567794 | A | G | -0.1197 | 1.18E-06 | 0.02459 | 0.0037 | 0.0182 | 0.8392 |
|  | rs57068864 | A | G | 0.196 | 6.80E-06 | 0.0435 | 0.0106 | 0.0248 | 0.6708 |
|  | rs59231583 | T | G | 0.1239 | 7.93E-06 | 0.02769 | 0.031 | 0.0192 | 0.107 |
|  | rs61500216 | C | T | 0.2173 | 4.05E-06 | 0.04709 | 0.0194 | 0.0276 | 0.4819 |
|  | rs6686155 | A | C | 0.2308 | 1.25E-06 | 0.04753 | 0.0194 | 0.0325 | 0.550201 |
|  | rs72779785 | G | A | -0.2122 | 1.36E-10 | 0.03296 | 0.0158 | 0.0369 | 0.668499 |
|  | rs73365443 | A | G | 0.1456 | 1.05E-06 | 0.02977 | -0.004 | 0.021 | 0.849 |
|  | rs74743779 | T | C | -0.2114 | 7.27E-06 | 0.04705 | -0.0485 | 0.0332 | 0.1441 |
|  | rs75495211 | T | G | -0.2997 | 6.88E-06 | 0.06655 | -0.0162 | 0.0618 | 0.793601 |
|  | rs75799568 | T | C | 1.176 | 4.39E-06 | 0.2558 | 0.0529 | 0.0396 | 0.1811 |
|  | rs75913581 | A | G | 0.1133 | 4.82E-06 | 0.02474 | -0.0054 | 0.0187 | 0.7706 |
|  | rs76443179 | A | G | 1.513 | 7.06E-06 | 0.3363 | 0.0891 | 0.0464 | 0.05496 |
|  | rs76997602 | G | A | -0.4225 | 3.67E-06 | 0.09112 | -0.0832 | 0.053 | 0.1163 |
|  | rs771140 | G | C | -0.1377 | 3.22E-06 | 0.02952 | 0.0223 | 0.0385 | 0.5634 |
|  | rs78581629 | A | G | -0.1869 | 4.78E-06 | 0.0408 | 0.0137 | 0.0386 | 0.722301 |
|  | rs78648104 | C | T | -0.1496 | 8.81E-06 | 0.03361 | 0.0262 | 0.0248 | 0.2906 |
|  | rs78744340 | A | G | -0.4444 | 4.76E-06 | 0.09699 | 0.0167 | 0.0614 | 0.785599 |
|  | rs79684162 | T | C | 0.4692 | 1.50E-06 | 0.09735 | 0.015 | 0.0403 | 0.7103 |
|  | rs9378213 | G | T | 0.2434 | 5.16E-15 | 0.03098 | -0.0385 | 0.0224 | 0.08586 |
| CD28+ CD45RA- CD8dim %CD8dim | rs10517119 | G | A | -0.1189 | 9.22E-06 | 0.02678 | 0.008 | 0.02 | 0.6879 |
|  | rs11579717 | A | G | -1.217 | 6.36E-11 | 0.1857 | -0.0033 | 0.0347 | 0.9245 |
|  | rs117626397 | G | A | -2.612 | 4.58E-06 | 0.5689 | 0.1619 | 0.0774 | 0.03643 |
|  | rs118141156 | G | C | 0.2511 | 7.20E-06 | 0.05587 | -0.0981 | 0.0482 | 0.04177 |
|  | rs12231396 | G | A | 0.1154 | 9.87E-06 | 0.02606 | -0.0109 | 0.0192 | 0.5693 |
|  | rs12576570 | A | G | -0.205 | 6.45E-06 | 0.04538 | 2.00E-04 | 0.0288 | 0.9936 |
|  | rs137931178 | G | A | 0.1721 | 3.37E-06 | 0.03698 | 0.032 | 0.1695 | 0.8503 |
|  | rs138915779 | A | C | -1.146 | 4.68E-07 | 0.227 | 0.0703 | 0.075 | 0.3486 |
|  | rs142531546 | T | C | -0.3326 | 4.80E-06 | 0.07262 | -0.0182 | 0.0811 | 0.8225 |
|  | rs147207959 | T | C | 1.093 | 8.95E-06 | 0.2458 | -0.0101 | 0.0753 | 0.8931 |
|  | rs1997323 | A | G | 0.9482 | 6.53E-06 | 0.21 | -0.1107 | 0.0595 | 0.06276 |
|  | rs2085746 | T | C | -0.2163 | 1.48E-06 | 0.04486 | 0.0093 | 0.0217 | 0.6672 |
|  | rs2177324 | A | G | -0.5618 | 1.01E-06 | 0.1147 | -0.0023 | 0.0465 | 0.9603 |
|  | rs2710824 | T | C | 0.1241 | 4.54E-06 | 0.02702 | -0.0169 | 0.0184 | 0.3591 |
|  | rs4796089 | C | T | 0.1982 | 5.93E-08 | 0.03647 | -0.042 | 0.0316 | 0.1835 |
|  | rs541367304 | A | G | -2.245 | 2.09E-18 | 0.255 | -0.1433 | 0.1366 | 0.2943 |
|  | rs55711612 | G | A | 0.1586 | 3.80E-07 | 0.03116 | 0.0139 | 0.0208 | 0.5046 |
|  | rs56206359 | G | A | -0.1189 | 6.71E-06 | 0.02636 | 0.01 | 0.0208 | 0.6313 |
|  | rs72694845 | C | A | -2.221 | 4.66E-06 | 0.4842 | 0.1255 | 0.0895 | 0.1611 |
|  | rs72774994 | T | C | -0.1388 | 9.84E-06 | 0.03136 | 0.0485 | 0.0217 | 0.02559 |
|  | rs7316570 | G | C | -0.1515 | 3.40E-06 | 0.03255 | 0.0193 | 0.0209 | 0.3559 |
|  | rs7649029 | G | A | -0.1828 | 3.77E-07 | 0.0359 | -0.0114 | 0.0225 | 0.612099 |
|  | rs7679241 | A | G | -0.1121 | 5.53E-06 | 0.02462 | 0.0027 | 0.0183 | 0.8845 |
|  | rs76919728 | G | T | 2.135 | 1.35E-06 | 0.441 | -0.0983 | 0.1257 | 0.4341 |
|  | rs78170509 | C | G | 0.1594 | 3.05E-06 | 0.03409 | 0.0214 | 0.0444 | 0.630099 |
|  | rs78336738 | T | C | -0.6195 | 2.98E-06 | 0.1324 | -0.0043 | 0.0419 | 0.9178 |
| CD28+ CD45RA+ CD8br %T cell | rs10183338 | T | G | 0.1132 | 0.02243 | 4.71E-07 | -0.0143 | 0.02 | 0.4764 |
|  | rs10973214 | G | A | 13.61 | 2.9 | 2.79E-06 | 0.1364 | 0.1183 | 0.2489 |
|  | rs111275136 | A | G | 2.141 | 0.4738 | 6.46E-06 | -0.0685 | 0.0653 | 0.2947 |
|  | rs111283043 | C | A | 4.463 | 0.7922 | 1.90E-08 | -0.0694 | 0.0633 | 0.2728 |
|  | rs111346930 | T | C | -0.1678 | 0.0379 | 9.82E-06 | -0.0443 | 0.0374 | 0.2362 |
|  | rs111503027 | A | G | 3.141 | 0.639 | 9.27E-07 | -0.0126 | 0.0956 | 0.8952 |
|  | rs112258643 | T | C | 3.436 | 0.6021 | 1.25E-08 | 0.0613 | 0.1172 | 0.6008 |
|  | rs112280034 | G | A | 4.67 | 0.9514 | 9.63E-07 | -0.1671 | 0.1957 | 0.3932 |
|  | rs112471936 | A | T | 9.954 | 1.725 | 8.55E-09 | -0.0189 | 0.0469 | 0.686501 |
|  | rs112571466 | G | A | 3.787 | 0.6773 | 2.44E-08 | -0.0095 | 0.0763 | 0.9005 |
|  | rs112637846 | A | C | 2.345 | 0.4689 | 5.96E-07 | -0.0734 | 0.0491 | 0.1348 |
|  | rs112731098 | A | G | 1.692 | 0.3573 | 2.26E-06 | 0.0688 | 0.0518 | 0.1837 |
|  | rs112779860 | A | G | 2.492 | 0.5273 | 2.39E-06 | 0.0029 | 0.0972 | 0.9762 |
|  | rs113046419 | C | T | 2.579 | 0.5321 | 1.31E-06 | 0.0378 | 0.0573 | 0.51 |
|  | rs113116201 | C | T | 0.6358 | 0.1227 | 2.30E-07 | 0.0949 | 0.0879 | 0.2798 |
|  | rs113447229 | C | T | 4.765 | 0.8459 | 1.92E-08 | 0.0733 | 0.0664 | 0.2698 |
|  | rs114795342 | T | A | 1.407 | 0.2995 | 2.73E-06 | -0.041 | 0.0541 | 0.4484 |
|  | rs115077170 | G | T | 7.992 | 1.404 | 1.37E-08 | -0.0794 | 0.0816 | 0.3308 |
|  | rs115218435 | G | T | 4.272 | 0.7543 | 1.60E-08 | 0.0529 | 0.0673 | 0.4319 |
|  | rs116131582 | C | T | -0.7833 | 0.1645 | 2.01E-06 | -0.1477 | 0.0636 | 0.02016 |
|  | rs11652705 | G | A | -0.1174 | 0.02339 | 5.52E-07 | -0.0104 | 0.0202 | 0.6052 |
|  | rs116562707 | G | A | 0.4024 | 0.06819 | 3.97E-09 | -0.0099 | 0.0439 | 0.8223 |
|  | rs116767883 | T | C | 9.1 | 1.583 | 9.88E-09 | 0.0027 | 0.0753 | 0.9713 |
|  | rs116954000 | A | T | 0.7802 | 0.1463 | 1.04E-07 | 0.1495 | 0.086 | 0.08212 |
|  | rs116984539 | T | C | 8.74 | 1.55 | 1.83E-08 | -0.076 | 0.0881 | 0.3886 |
|  | rs11735841 | A | G | 0.4503 | 0.09716 | 3.72E-06 | 0.0206 | 0.0609 | 0.7349 |
|  | rs117403703 | C | T | 3.353 | 0.6161 | 5.62E-08 | 0.0119 | 0.0592 | 0.8401 |
|  | rs117475733 | T | C | 1.818 | 0.2979 | 1.17E-09 | 0.0934 | 0.0705 | 0.1851 |
|  | rs117494082 | A | G | 3.894 | 0.6914 | 1.94E-08 | -0.0063 | 0.153 | 0.9672 |
|  | rs117795441 | A | G | 2.115 | 0.4548 | 3.42E-06 | -0.0515 | 0.0737 | 0.4845 |
|  | rs117927481 | A | G | 3.783 | 0.6683 | 1.63E-08 | -0.0919 | 0.0826 | 0.266 |
|  | rs118016388 | C | T | 7.82 | 1.424 | 4.27E-08 | 0.0088 | 0.0626 | 0.8882 |
|  | rs118127261 | A | G | 3.614 | 0.6762 | 9.65E-08 | -0.1013 | 0.1237 | 0.4128 |
|  | rs12258605 | A | T | 0.1289 | 0.02859 | 6.75E-06 | -0.0866 | 0.0278 | 0.001864 |
|  | rs12314907 | G | A | 6.244 | 1.145 | 5.33E-08 | -0.0465 | 0.0324 | 0.1516 |
|  | rs12755474 | G | A | 6.643 | 1.199 | 3.22E-08 | 0.0532 | 0.0783 | 0.4972 |
|  | rs13115676 | A | G | -0.1825 | 0.03943 | 3.84E-06 | -0.0287 | 0.0341 | 0.3992 |
|  | rs13243221 | G | T | 2.985 | 0.6167 | 1.35E-06 | -0.0554 | 0.0742 | 0.4558 |
|  | rs137858581 | C | T | 3.974 | 0.7251 | 4.54E-08 | 0.0466 | 0.0785 | 0.5532 |
|  | rs138649004 | A | C | 8.056 | 1.552 | 2.20E-07 | -0.0401 | 0.0576 | 0.4871 |
|  | rs138653244 | C | G | 2.288 | 0.4624 | 7.85E-07 | 0.0049 | 0.0413 | 0.9056 |
|  | rs138736730 | G | T | 2.587 | 0.526 | 9.13E-07 | 0.094 | 0.0754 | 0.2125 |
|  | rs139463423 | T | C | 1.613 | 0.356 | 6.03E-06 | 0.0713 | 0.1076 | 0.5075 |
|  | rs140064908 | T | C | 5.454 | 1.049 | 2.12E-07 | 0.028 | 0.0961 | 0.771101 |
|  | rs141341926 | T | G | 4.416 | 0.8469 | 1.95E-07 | 0.033 | 0.0542 | 0.5419 |
|  | rs141357353 | A | G | 1.943 | 0.4052 | 1.71E-06 | -0.0026 | 0.0823 | 0.9751 |
|  | rs141596789 | C | G | 1.57 | 0.2927 | 8.71E-08 | -0.0495 | 0.0648 | 0.4451 |
|  | rs141878021 | T | C | 3.021 | 0.6256 | 1.43E-06 | -0.1788 | 0.0641 | 0.005313 |
|  | rs142208889 | T | C | 3.814 | 0.6612 | 8.68E-09 | -0.0359 | 0.0904 | 0.6915 |
|  | rs142530179 | G | A | 3.783 | 0.6683 | 1.62E-08 | 0.0918 | 0.0593 | 0.1214 |
|  | rs142967914 | C | G | 3.794 | 0.6676 | 1.44E-08 | -0.0135 | 0.0433 | 0.7558 |
|  | rs143498795 | A | C | 3.717 | 0.67 | 3.11E-08 | -0.0044 | 0.0581 | 0.9402 |
|  | rs144547268 | C | T | 1.67 | 0.375 | 8.68E-06 | -0.0725 | 0.0709 | 0.3069 |
|  | rs144591335 | G | A | 3.946 | 0.6967 | 1.60E-08 | -0.0635 | 0.0576 | 0.2706 |
|  | rs144593458 | T | C | 3.122 | 0.6954 | 7.36E-06 | -0.0412 | 0.0532 | 0.4386 |
|  | rs144788631 | T | C | 3.533 | 0.6507 | 6.06E-08 | -0.0474 | 0.0849 | 0.5767 |
|  | rs145208064 | T | C | 1.818 | 0.403 | 6.66E-06 | -0.0561 | 0.0834 | 0.5013 |
|  | rs145827119 | T | C | 4.893 | 0.8386 | 5.91E-09 | -0.0673 | 0.0527 | 0.2018 |
|  | rs145865638 | T | C | 12.72 | 2.366 | 8.24E-08 | -0.1097 | 0.0389 | 0.004811 |
|  | rs146073003 | T | C | 7.599 | 1.346 | 1.80E-08 | -0.1119 | 0.0936 | 0.2318 |
|  | rs146985291 | G | A | 6.493 | 1.126 | 8.69E-09 | 0.061 | 0.0655 | 0.3513 |
|  | rs147066619 | T | A | 5.2 | 1.132 | 4.52E-06 | 0.0464 | 0.1137 | 0.683 |
|  | rs148064908 | A | G | 11.05 | 2.095 | 1.42E-07 | 0.0738 | 0.0622 | 0.2353 |
|  | rs149023182 | G | A | 4.52 | 0.7897 | 1.13E-08 | 0.0664 | 0.0878 | 0.4493 |
|  | rs149926070 | A | G | 3.533 | 0.6368 | 3.12E-08 | 0.2616 | 0.1898 | 0.1682 |
|  | rs149948027 | T | C | 2.886 | 0.5527 | 1.88E-07 | 0.058 | 0.0674 | 0.3896 |
|  | rs150096082 | A | G | 10.96 | 2.094 | 1.77E-07 | -0.033 | 0.0756 | 0.6625 |
|  | rs17292948 | T | C | 7.692 | 1.481 | 2.16E-07 | -0.0603 | 0.0498 | 0.2263 |
|  | rs17685199 | C | A | -0.1143 | 0.02221 | 2.82E-07 | -0.0307 | 0.035 | 0.3811 |
|  | rs179609 | T | G | 0.09014 | 0.01925 | 2.95E-06 | -0.0028 | 0.0195 | 0.884 |
|  | rs182236332 | G | A | 4.848 | 0.8097 | 2.34E-09 | -0.1661 | 0.1431 | 0.2459 |
|  | rs184082746 | C | T | 4.025 | 0.6399 | 3.59E-10 | -0.0849 | 0.0748 | 0.2566 |
|  | rs184134422 | T | C | 2.468 | 0.5223 | 2.40E-06 | 0.1191 | 0.0861 | 0.1666 |
|  | rs188748536 | C | T | 13.7 | 2.197 | 5.06E-10 | -0.0655 | 0.0512 | 0.2008 |
|  | rs189801254 | A | C | 4.11 | 0.7256 | 1.60E-08 | 0.0922 | 0.1353 | 0.4956 |
|  | rs192267028 | T | A | 9.558 | 1.918 | 6.53E-07 | 0.1193 | 0.0588 | 0.04241 |
|  | rs192475369 | T | A | 5.704 | 1.002 | 1.35E-08 | -0.0281 | 0.0674 | 0.6772 |
|  | rs2075682 | T | A | 0.1213 | 0.02348 | 2.51E-07 | -9.00E-04 | 0.0288 | 0.9763 |
|  | rs28460753 | A | G | 0.6042 | 0.1196 | 4.65E-07 | 0.0249 | 0.0406 | 0.5394 |
|  | rs35890579 | T | G | 0.6544 | 0.1439 | 5.58E-06 | -0.0348 | 0.049 | 0.4771 |
|  | rs3923561 | A | G | 7.88 | 1.638 | 1.58E-06 | -0.0208 | 0.0535 | 0.6977 |
|  | rs56095154 | C | G | 0.3131 | 0.06931 | 6.49E-06 | -0.0155 | 0.0394 | 0.6944 |
|  | rs56125740 | G | A | 2.385 | 0.4755 | 5.55E-07 | 0.0222 | 0.0381 | 0.5606 |
|  | rs571177836 | T | G | 8.771 | 1.619 | 6.45E-08 | -0.0038 | 0.0359 | 0.9162 |
|  | rs58471414 | G | T | 3.833 | 0.6771 | 1.63E-08 | 0.008 | 0.0666 | 0.9043 |
|  | rs60455861 | G | T | 0.08737 | 0.01887 | 3.81E-06 | 0.0057 | 0.0233 | 0.807 |
|  | rs62477133 | G | C | 2.164 | 0.4684 | 4.00E-06 | -0.0883 | 0.0908 | 0.3313 |
|  | rs6833962 | T | C | -0.1202 | 0.02413 | 6.57E-07 | -0.0172 | 0.0398 | 0.6655 |
|  | rs72968960 | G | A | 0.6373 | 0.1276 | 6.14E-07 | -0.0988 | 0.056 | 0.07782 |
|  | rs73404248 | A | G | -0.1468 | 0.03049 | 1.54E-06 | -0.0059 | 0.0504 | 0.9063 |
|  | rs73648165 | G | C | 2.119 | 0.4729 | 7.68E-06 | 0.0602 | 0.0614 | 0.3271 |
|  | rs74577787 | T | C | 2.349 | 0.4689 | 5.75E-07 | 0.1317 | 0.0829 | 0.112 |
|  | rs74723190 | C | A | 6.235 | 1.209 | 2.66E-07 | 0.1155 | 0.1037 | 0.2653 |
|  | rs74926697 | A | G | 2.169 | 0.4751 | 5.16E-06 | -0.0728 | 0.1203 | 0.544999 |
|  | rs75069185 | C | T | 6.736 | 1.26 | 9.50E-08 | -0.062 | 0.051 | 0.2235 |
|  | rs7567302 | G | C | -0.08589 | 0.0179 | 1.66E-06 | 0.0286 | 0.0185 | 0.1219 |
|  | rs7583259 | G | C | 0.104 | 0.01759 | 3.71E-09 | -0.0214 | 0.0184 | 0.2441 |
|  | rs76325389 | G | A | 4.877 | 0.8599 | 1.53E-08 | -0.0812 | 0.0639 | 0.2038 |
|  | rs76604940 | C | A | 3.867 | 0.6826 | 1.59E-08 | -0.0326 | 0.062 | 0.599601 |
|  | rs77414299 | T | C | 4.312 | 0.8683 | 7.16E-07 | -0.0239 | 0.0577 | 0.6787 |
|  | rs77458346 | G | A | 1.609 | 0.3292 | 1.06E-06 | -0.059 | 0.0622 | 0.3431 |
|  | rs77670766 | A | G | 4.66 | 0.8838 | 1.43E-07 | -0.0629 | 0.0708 | 0.3741 |
|  | rs77780671 | C | T | 0.4657 | 0.1052 | 9.82E-06 | -0.1099 | 0.1069 | 0.304 |
|  | rs77935824 | G | A | 3.755 | 0.6644 | 1.72E-08 | 0.1665 | 0.1432 | 0.2451 |
|  | rs78743764 | A | C | 2.171 | 0.4586 | 2.29E-06 | 0.1113 | 0.0693 | 0.1083 |
|  | rs79112776 | T | C | 1.676 | 0.3604 | 3.44E-06 | 0.0123 | 0.0389 | 0.752501 |
|  | rs80194890 | G | T | 4.68 | 0.8111 | 8.65E-09 | 0.0342 | 0.0618 | 0.580801 |
|  | rs8067835 | G | A | 0.1025 | 0.02096 | 1.05E-06 | 0.0144 | 0.0269 | 0.592199 |
|  | rs8180936 | T | C | 0.08508 | 0.01843 | 4.05E-06 | 0.0069 | 0.0193 | 0.719399 |
|  | rs9592479 | C | G | 4.763 | 0.8417 | 1.65E-08 | 0.0849 | 0.0706 | 0.2291 |
| CD45RA- CD28- CD8br %CD8br | rs10970967 | G | T | 1.525 | 0.3153 | 1.37E-06 | 0.0033 | 0.0225 | 0.8845 |
|  | rs112774387 | G | A | 20.96 | 3.949 | 1.18E-07 | 0.0066 | 0.057 | 0.9076 |
|  | rs113679275 | C | A | 7.293 | 1.623 | 7.22E-06 | -0.137 | 0.1093 | 0.2102 |
|  | rs114135956 | C | T | 53.93 | 8.721 | 6.98E-10 | 0.0465 | 0.0733 | 0.5257 |
|  | rs114216104 | T | C | 56.58 | 11.01 | 2.91E-07 | -0.1544 | 0.0611 | 0.01146 |
|  | rs116077033 | T | C | 37.63 | 8.283 | 5.73E-06 | 0.1005 | 0.0589 | 0.087989 |
|  | rs117428784 | A | C | 36.89 | 8.022 | 4.41E-06 | 0.0153 | 0.1213 | 0.8995 |
|  | rs12791920 | G | A | -1.512 | 0.306 | 8.14E-07 | -0.0195 | 0.0185 | 0.2924 |
|  | rs130071 | A | G | 2.545 | 0.4129 | 7.93E-10 | -0.0324 | 0.022 | 0.1405 |
|  | rs138754438 | T | C | 14.12 | 3.162 | 8.29E-06 | 0.0693 | 0.0665 | 0.2976 |
|  | rs138758802 | T | C | 16.73 | 3.78 | 9.90E-06 | -0.0556 | 0.0473 | 0.24 |
|  | rs140385926 | G | A | 28.75 | 5.206 | 3.59E-08 | 0.102 | 0.0604 | 0.091361 |
|  | rs142399295 | T | A | 18.12 | 4.089 | 9.62E-06 | 0.0094 | 0.0701 | 0.8929 |
|  | rs142507918 | A | G | 11.97 | 2.464 | 1.23E-06 | 2.00E-04 | 0.0528 | 0.9972 |
|  | rs148265436 | T | C | 5.121 | 1.023 | 5.91E-07 | -0.0373 | 0.0373 | 0.3179 |
|  | rs149487182 | T | C | 36.99 | 7.317 | 4.54E-07 | -0.0321 | 0.0646 | 0.619699 |
|  | rs149600977 | G | A | 19.53 | 4.148 | 2.59E-06 | -0.0166 | 0.0463 | 0.7195 |
|  | rs149716976 | G | A | 8.664 | 1.937 | 8.01E-06 | 0.0071 | 0.0425 | 0.8672 |
|  | rs150146567 | A | T | 15.46 | 3.422 | 6.51E-06 | 0.0371 | 0.0773 | 0.6311 |
|  | rs150856260 | T | C | 28.31 | 6.128 | 4.00E-06 | 0.0372 | 0.082 | 0.6502 |
|  | rs151237482 | C | A | 26.26 | 5.048 | 2.10E-07 | -0.0413 | 0.0696 | 0.5525 |
|  | rs151336928 | C | T | 7.629 | 1.661 | 4.55E-06 | 0.008 | 0.042 | 0.8485 |
|  | rs17212021 | C | T | -2.715 | 0.473 | 1.03E-08 | -0.0365 | 0.0629 | 0.562 |
|  | rs1736913 | C | T | 2.095 | 0.3672 | 1.26E-08 | 0.0025 | 0.0197 | 0.8981 |
|  | rs17388638 | A | G | 20.89 | 4.337 | 1.52E-06 | 0.0382 | 0.0445 | 0.3906 |
|  | rs17556536 | T | A | 3.019 | 0.6515 | 3.71E-06 | -0.0171 | 0.0273 | 0.5308 |
|  | rs190312656 | A | G | 26.32 | 4.629 | 1.42E-08 | 0.0581 | 0.0691 | 0.4003 |
|  | rs3133835 | A | G | 2.722 | 0.584 | 3.28E-06 | -0.0094 | 0.0292 | 0.7484 |
|  | rs450175 | C | A | -1.704 | 0.3071 | 3.08E-08 | -0.0155 | 0.0184 | 0.3981 |
|  | rs538264691 | C | A | 38.41 | 8.177 | 2.74E-06 | 0.185 | 0.1068 | 0.08328 |
|  | rs55881385 | T | G | 55.35 | 10.46 | 1.31E-07 | 0.0442 | 0.0454 | 0.3301 |
|  | rs55896100 | T | C | 7.798 | 1.675 | 3.36E-06 | -0.0109 | 0.0533 | 0.8383 |
|  | rs58905133 | G | A | 3.541 | 0.3183 | 2.91E-28 | 3.00E-04 | 0.0184 | 0.985 |
|  | rs6001335 | C | G | -1.811 | 0.3762 | 1.53E-06 | 0.0225 | 0.0189 | 0.2333 |
|  | rs61188057 | G | C | 5.753 | 1.259 | 5.04E-06 | 0.0063 | 0.0363 | 0.8624 |
|  | rs62033204 | A | T | 19.65 | 4.049 | 1.27E-06 | 0.1666 | 0.0875 | 0.05696 |
|  | rs62488802 | T | C | 47.63 | 9.619 | 7.71E-07 | 0.1132 | 0.0525 | 0.03113 |
|  | rs66657257 | G | T | 2.142 | 0.472 | 5.86E-06 | 0.0104 | 0.0238 | 0.662599 |
|  | rs66707192 | A | G | 1.892 | 0.4138 | 5.00E-06 | -0.0278 | 0.0207 | 0.1797 |
|  | rs6941748 | T | C | 1.524 | 0.3207 | 2.12E-06 | -0.0215 | 0.0211 | 0.3086 |
|  | rs72815709 | C | T | 18.44 | 3.073 | 2.16E-09 | -0.0188 | 0.0494 | 0.703301 |
|  | rs72978357 | A | G | 19.06 | 3.981 | 1.76E-06 | 0.0512 | 0.0402 | 0.2036 |
|  | rs742867 | T | C | 14.88 | 2.692 | 3.49E-08 | 0.1579 | 0.1395 | 0.2577 |
|  | rs75420474 | C | T | 44.86 | 8.442 | 1.14E-07 | 0.0524 | 0.0595 | 0.3784 |
|  | rs75427771 | A | C | 14.23 | 3.197 | 8.77E-06 | -0.1024 | 0.0996 | 0.3039 |
|  | rs75573571 | A | T | 10.34 | 2.307 | 7.57E-06 | 0.0536 | 0.0633 | 0.3971 |
|  | rs76343453 | C | T | 18.5 | 3.684 | 5.37E-07 | -0.1005 | 0.2373 | 0.6718 |
|  | rs77920451 | G | A | 5.131 | 1.11 | 3.94E-06 | -0.0333 | 0.0749 | 0.656299 |
|  | rs77946649 | A | G | 27.72 | 5.936 | 3.13E-06 | 0.0692 | 0.0589 | 0.2399 |
|  | rs77953154 | T | C | 6.375 | 1.362 | 2.95E-06 | 0.0196 | 0.0395 | 0.6193 |
|  | rs9468632 | T | C | -2.744 | 0.5907 | 3.52E-06 | -0.0073 | 0.0753 | 0.9231 |
| CD45RA+ CD28- CD8br %CD8br | rs117284243 | T | A | 64.01 | 3.08E-06 | 13.7 | -0.1037 | 0.0605 | 0.08673 |
|  | rs117858699 | T | C | 7.144 | 1.62E-06 | 1.487 | 0.0136 | 0.043 | 0.751799 |
|  | rs13133130 | G | A | 1.65 | 2.33E-06 | 0.3487 | 0.0198 | 0.019 | 0.2992 |
|  | rs139329941 | T | C | 11.48 | 3.37E-06 | 2.466 | -0.136 | 0.09 | 0.131 |
|  | rs144378515 | G | C | 9.884 | 3.04E-06 | 2.114 | -0.0266 | 0.0809 | 0.742699 |
|  | rs146620586 | T | G | 18.78 | 2.97E-06 | 4.013 | -0.058 | 0.1248 | 0.642 |
|  | rs147119730 | T | A | 9.832 | 8.65E-06 | 2.207 | 0.0297 | 0.038 | 0.4355 |
|  | rs147841321 | T | C | 17.82 | 5.13E-06 | 3.901 | 0.0762 | 0.1549 | 0.6229 |
|  | rs148524572 | C | T | 11.65 | 1.48E-06 | 2.417 | -0.0583 | 0.0592 | 0.3254 |
|  | rs150885241 | A | G | 42.58 | 4.46E-06 | 9.265 | -0.0525 | 0.0553 | 0.3421 |
|  | rs17175340 | T | C | 2.222 | 5.62E-06 | 0.4886 | 0.0263 | 0.0212 | 0.2145 |
|  | rs1747869 | C | G | 1.727 | 6.68E-06 | 0.3828 | -0.0377 | 0.0198 | 0.05762 |
|  | rs184701860 | G | A | 18.22 | 1.56E-06 | 3.787 | -0.0222 | 0.0909 | 0.807 |
|  | rs199797711 | C | T | 37.99 | 5.20E-09 | 6.487 | -0.0052 | 0.078 | 0.9464 |
|  | rs375356899 | G | A | 9.772 | 1.93E-07 | 1.873 | -0.0198 | 0.0644 | 0.758799 |
|  | rs45609839 | T | G | 5.923 | 8.00E-06 | 1.324 | 0.1041 | 0.0508 | 0.04039 |
|  | rs4648125 | A | G | 36.64 | 6.72E-08 | 6.772 | -0.0092 | 0.0751 | 0.903 |
|  | rs4796089 | C | T | -2.611 | 4.62E-07 | 0.517 | -0.042 | 0.0316 | 0.1835 |
|  | rs4853078 | G | T | 17.66 | 6.74E-06 | 3.916 | 0.1618 | 0.0502 | 0.001283 |
|  | rs62461164 | C | G | 3.528 | 9.46E-06 | 0.7953 | -0.005 | 0.0311 | 0.8726 |
|  | rs7186214 | C | T | 2.946 | 2.14E-06 | 0.6206 | 0.0248 | 0.0267 | 0.3537 |
|  | rs73719023 | C | G | 6.923 | 5.05E-07 | 1.375 | 0.0011 | 0.1151 | 0.9925 |
|  | rs74055305 | T | C | -2.987 | 6.48E-06 | 0.6613 | -0.0474 | 0.0392 | 0.2266 |
|  | rs74828441 | C | A | 10.36 | 3.55E-07 | 2.03 | 0.0127 | 0.0781 | 0.8706 |
|  | rs7559619 | G | A | -2.745 | 1.96E-13 | 0.372 | 3.00E-04 | 0.0184 | 0.985 |
|  | rs7605634 | C | T | 1.721 | 7.70E-06 | 0.3841 | 0.0131 | 0.0202 | 0.5144 |
|  | rs76444609 | A | G | 60.43 | 8.67E-06 | 13.57 | -0.044 | 0.0589 | 0.4552 |
|  | rs76552842 | A | G | 59.5 | 5.82E-06 | 13.1 | -0.1001 | 0.0831 | 0.2283 |
|  | rs7819099 | G | A | 1.611 | 4.22E-06 | 0.3496 | -0.0257 | 0.0186 | 0.1672 |
|  | rs78750136 | C | T | 130.7 | 1.92E-06 | 27.4 | -0.1154 | 0.0551 | 0.03638 |
|  | rs79638560 | A | C | 27.8 | 4.48E-06 | 6.049 | -0.0385 | 0.1297 | 0.7663 |
|  | rs7989683 | T | C | -3.328 | 4.66E-06 | 0.7255 | 0.0088 | 0.0306 | 0.7742 |
|  | rs80181351 | T | C | 16.82 | 7.27E-06 | 3.744 | 0.0453 | 0.0975 | 0.6422 |
| CD45RA+ CD28- CD8br %T cell | rs10274897 | G | T | 19.66 | 4.1 | 1.70E-06 | -0.0983 | 0.1306 | 0.4517 |
|  | rs10492710 | A | G | 5.636 | 1.205 | 3.02E-06 | 0.0189 | 0.0571 | 0.740399 |
|  | rs10505739 | C | A | 18.81 | 3.393 | 3.21E-08 | 0.0085 | 0.042 | 0.84 |
|  | rs10769706 | A | T | -0.9132 | 0.2061 | 9.66E-06 | 0.0531 | 0.038 | 0.1615 |
|  | rs10824158 | T | G | 9.768 | 1.725 | 1.62E-08 | 0.0737 | 0.0351 | 0.03582 |
|  | rs1106305 | T | C | 2.681 | 0.5792 | 3.82E-06 | 0.0374 | 0.0775 | 0.6292 |
|  | rs111065494 | T | A | 4.133 | 0.8427 | 9.81E-07 | -0.1559 | 0.1213 | 0.1988 |
|  | rs111747460 | A | G | 26.82 | 4.296 | 4.81E-10 | -0.182 | 0.1516 | 0.2299 |
|  | rs111972324 | A | C | 9.939 | 2.208 | 7.00E-06 | -0.12 | 0.0677 | 0.076201 |
|  | rs11200733 | A | G | 5.664 | 1.165 | 1.21E-06 | 0.0367 | 0.0444 | 0.409 |
|  | rs11202534 | T | G | 13.13 | 2.792 | 2.66E-06 | -0.0123 | 0.04 | 0.7591 |
|  | rs112048022 | G | T | 9.197 | 2.038 | 6.58E-06 | -0.0121 | 0.0424 | 0.775001 |
|  | rs112254351 | A | G | 19.55 | 3.788 | 2.60E-07 | -0.123 | 0.0426 | 0.00392 |
|  | rs113007178 | A | T | 7.787 | 1.749 | 8.73E-06 | 0.1548 | 0.2607 | 0.5526 |
|  | rs113598754 | G | A | 11.3 | 2.457 | 4.39E-06 | 0.0478 | 0.0489 | 0.3291 |
|  | rs114021246 | C | G | 13.26 | 2.594 | 3.34E-07 | -0.0843 | 0.0542 | 0.12 |
|  | rs114219959 | T | G | 14.57 | 3.276 | 8.91E-06 | 0.0335 | 0.0751 | 0.656 |
|  | rs114230155 | A | G | 9.265 | 1.585 | 5.47E-09 | -0.002 | 0.07 | 0.9773 |
|  | rs114430346 | A | G | 20.36 | 4.31 | 2.42E-06 | 0.2072 | 0.0784 | 0.008249 |
|  | rs114559319 | A | T | 18.49 | 3.731 | 7.51E-07 | -0.1115 | 0.0603 | 0.06469 |
|  | rs114581990 | G | A | 8.874 | 1.564 | 1.51E-08 | -0.052 | 0.1116 | 0.641 |
|  | rs114619811 | G | A | 15.63 | 2.895 | 7.15E-08 | 0.0271 | 0.0492 | 0.5815 |
|  | rs114672498 | G | C | 7.853 | 1.619 | 1.28E-06 | -0.0204 | 0.0756 | 0.7871 |
|  | rs114685875 | A | G | 17.06 | 3.509 | 1.22E-06 | 0.0106 | 0.0728 | 0.8844 |
|  | rs114827401 | T | A | 10.71 | 2.132 | 5.39E-07 | -0.0105 | 0.0248 | 0.6709 |
|  | rs115715247 | T | C | 27.58 | 5.685 | 1.28E-06 | 0.1818 | 0.2395 | 0.4479 |
|  | rs11576563 | T | C | 13.78 | 2.988 | 4.16E-06 | 0.0186 | 0.0437 | 0.6712 |
|  | rs11580627 | C | T | 15.69 | 3.028 | 2.33E-07 | 0.0156 | 0.0394 | 0.6917 |
|  | rs115879183 | G | A | 12.99 | 2.578 | 4.96E-07 | -0.0896 | 0.0826 | 0.278 |
|  | rs115943331 | A | G | 39.6 | 8.915 | 9.17E-06 | 0.1107 | 0.1838 | 0.547 |
|  | rs116399750 | A | G | 17.96 | 3.205 | 2.27E-08 | 0.0078 | 0.0635 | 0.9026 |
|  | rs116581718 | C | T | 22.4 | 3.301 | 1.35E-11 | -0.1229 | 0.0872 | 0.1589 |
|  | rs116915023 | T | C | 10.65 | 1.782 | 2.50E-09 | 0.0111 | 0.082 | 0.8921 |
|  | rs116916598 | G | A | 19.07 | 3.407 | 2.33E-08 | -0.0248 | 0.1848 | 0.8932 |
|  | rs116979366 | A | T | 5.888 | 1.203 | 1.04E-06 | -0.0439 | 0.0869 | 0.6132 |
|  | rs117038196 | A | G | 11.96 | 2.665 | 7.38E-06 | 0.0084 | 0.0464 | 0.8562 |
|  | rs117173207 | A | G | 18.77 | 3.342 | 2.10E-08 | -0.1 | 0.1273 | 0.4321 |
|  | rs117284243 | T | A | 49.65 | 5.795 | 1.57E-17 | -0.1037 | 0.0605 | 0.08673 |
|  | rs117296998 | T | C | 10.5 | 2.211 | 2.10E-06 | -0.0586 | 0.0498 | 0.2394 |
|  | rs117469911 | A | C | 6.228 | 1.155 | 7.39E-08 | -0.0255 | 0.0891 | 0.774399 |
|  | rs117478414 | T | C | 32.87 | 5.796 | 1.53E-08 | 0.0392 | 0.1225 | 0.7493 |
|  | rs117692894 | T | G | 33.95 | 5.804 | 5.41E-09 | -0.0307 | 0.0494 | 0.5351 |
|  | rs117708929 | C | T | 3.153 | 0.6419 | 9.43E-07 | -0.0636 | 0.2489 | 0.7983 |
|  | rs117785622 | T | G | 13.02 | 2.395 | 5.79E-08 | 0.0281 | 0.0601 | 0.6399 |
|  | rs117808048 | A | G | 6.969 | 1.557 | 7.90E-06 | 0.022 | 0.1822 | 0.904 |
|  | rs117867588 | A | G | 36.56 | 6.272 | 6.06E-09 | 0.0029 | 0.0823 | 0.972 |
|  | rs118089863 | T | C | 12.62 | 2.243 | 1.98E-08 | -0.0057 | 0.0587 | 0.9231 |
|  | rs118180479 | C | T | 11.65 | 2.356 | 7.99E-07 | 0.0415 | 0.0744 | 0.577 |
|  | rs12068756 | C | T | 2.264 | 0.4741 | 1.86E-06 | -0.0658 | 0.0772 | 0.3938 |
|  | rs12279408 | T | C | 16 | 2.92 | 4.60E-08 | 0.034 | 0.1656 | 0.8372 |
|  | rs12450105 | C | T | 6.576 | 1.424 | 4.02E-06 | 0.0111 | 0.0488 | 0.8199 |
|  | rs12499962 | G | A | 25.15 | 3.141 | 1.59E-15 | 0.0824 | 0.0486 | 0.089861 |
|  | rs12501785 | A | G | 10.8 | 1.881 | 1.00E-08 | 0.0669 | 0.039 | 0.085801 |
|  | rs12596264 | A | G | 7.351 | 1.534 | 1.71E-06 | -0.0229 | 0.0638 | 0.719 |
|  | rs132751 | T | C | 11.84 | 2.178 | 5.73E-08 | -0.0829 | 0.0514 | 0.1064 |
|  | rs137942933 | T | G | 23.37 | 3.154 | 1.58E-13 | 0.1342 | 0.0887 | 0.1303 |
|  | rs138327786 | A | G | 21.5 | 3.886 | 3.38E-08 | -0.1022 | 0.0536 | 0.05644 |
|  | rs138410422 | A | G | 28.99 | 5.954 | 1.17E-06 | -0.0461 | 0.0695 | 0.507 |
|  | rs138547687 | A | G | 11.19 | 2.452 | 5.22E-06 | 0.0568 | 0.0889 | 0.5224 |
|  | rs138697156 | G | A | 12.79 | 2.872 | 8.71E-06 | 0.0771 | 0.0852 | 0.3656 |
|  | rs138765259 | G | T | 15.44 | 2.921 | 1.33E-07 | -0.0235 | 0.0776 | 0.7625 |
|  | rs138766897 | T | A | 4.991 | 0.9808 | 3.81E-07 | -0.0384 | 0.0871 | 0.6595 |
|  | rs138901088 | A | C | 12.28 | 2.651 | 3.74E-06 | 0.0914 | 0.0657 | 0.1646 |
|  | rs138937927 | T | C | 23.94 | 3.906 | 9.81E-10 | 0.0855 | 0.0529 | 0.1062 |
|  | rs139329941 | T | C | 5.185 | 1.061 | 1.07E-06 | -0.136 | 0.09 | 0.131 |
|  | rs139898536 | T | A | 5.214 | 1.178 | 9.86E-06 | 0.0406 | 0.3761 | 0.914 |
|  | rs140963286 | T | C | 33.4 | 5.736 | 6.30E-09 | 0.0422 | 0.064 | 0.5098 |
|  | rs140976870 | T | C | 3.271 | 0.7129 | 4.64E-06 | 0.0153 | 0.0953 | 0.8728 |
|  | rs141044525 | T | C | 7.807 | 1.526 | 3.27E-07 | 0.0056 | 0.131 | 0.9658 |
|  | rs141656769 | T | G | 14.34 | 2.896 | 7.73E-07 | -0.0272 | 0.0526 | 0.6058 |
|  | rs141850210 | A | G | 9.155 | 1.747 | 1.71E-07 | 0.0225 | 0.0511 | 0.6598 |
|  | rs142866937 | C | A | 4.265 | 0.9493 | 7.28E-06 | -0.0025 | 0.0624 | 0.9685 |
|  | rs142884906 | A | G | 13.5 | 2.962 | 5.34E-06 | -0.0425 | 0.0746 | 0.568499 |
|  | rs143129647 | T | C | 15.69 | 3.443 | 5.37E-06 | 0.042 | 0.1047 | 0.6884 |
|  | rs143395500 | T | C | 51.17 | 8.845 | 7.92E-09 | -0.0696 | 0.0514 | 0.1754 |
|  | rs143845801 | C | G | 18.54 | 2.753 | 1.90E-11 | -0.012 | 0.0331 | 0.7168 |
|  | rs144104650 | A | G | 15.28 | 3.08 | 7.31E-07 | -0.0586 | 0.0679 | 0.3881 |
|  | rs144275943 | G | T | 19.21 | 4.183 | 4.53E-06 | -0.0792 | 0.0678 | 0.2425 |
|  | rs144613377 | C | T | 3.834 | 0.7616 | 5.04E-07 | 0.0165 | 0.0726 | 0.8208 |
|  | rs145410896 | G | C | 5.395 | 1.046 | 2.66E-07 | 0.0217 | 0.0449 | 0.628699 |
|  | rs146171997 | G | A | 35.54 | 7.417 | 1.73E-06 | -0.0081 | 0.1215 | 0.947 |
|  | rs146374482 | A | G | 19.45 | 3.678 | 1.31E-07 | -0.0205 | 0.2403 | 0.932 |
|  | rs146627868 | A | G | 2.823 | 0.6163 | 4.79E-06 | -0.0677 | 0.1635 | 0.6786 |
|  | rs146650864 | A | G | 9.123 | 1.757 | 2.19E-07 | -0.0061 | 0.067 | 0.9271 |
|  | rs146822590 | T | A | 14.19 | 2.379 | 2.68E-09 | -0.0917 | 0.0696 | 0.1878 |
|  | rs147093663 | T | G | 42.63 | 7.413 | 9.65E-09 | 0.1892 | 0.1137 | 0.096079 |
|  | rs147107195 | T | C | 23.9 | 4.049 | 3.92E-09 | -0.0061 | 0.0669 | 0.9268 |
|  | rs147207602 | A | G | 25.5 | 5.734 | 8.99E-06 | 0.0485 | 0.0468 | 0.3001 |
|  | rs147572731 | T | C | 14.48 | 3.001 | 1.44E-06 | -0.0275 | 0.0672 | 0.6826 |
|  | rs147600774 | T | A | 2.407 | 0.5345 | 6.91E-06 | -0.0392 | 0.0752 | 0.601999 |
|  | rs147841321 | T | C | 8.69 | 1.67 | 2.08E-07 | 0.0762 | 0.1549 | 0.6229 |
|  | rs147968748 | G | A | 4.517 | 0.9486 | 2.00E-06 | 0.0512 | 0.0873 | 0.5573 |
|  | rs148291161 | G | A | 14.11 | 2.744 | 2.86E-07 | 0.0802 | 0.094 | 0.3936 |
|  | rs148658406 | T | C | 32.81 | 5.716 | 1.02E-08 | 0.0403 | 0.0563 | 0.474 |
|  | rs148947489 | T | C | 8.302 | 1.533 | 6.58E-08 | -0.0072 | 0.0482 | 0.8814 |
|  | rs149021870 | G | A | 3.698 | 0.7669 | 1.49E-06 | 0.0293 | 0.066 | 0.6576 |
|  | rs149050426 | C | G | 12.68 | 2.647 | 1.72E-06 | -0.087 | 0.0619 | 0.16 |
|  | rs149208831 | C | A | 25.69 | 4.095 | 3.97E-10 | -0.1594 | 0.0574 | 0.005463 |
|  | rs149394991 | A | C | 15.07 | 2.977 | 4.40E-07 | -0.1751 | 0.1327 | 0.1871 |
|  | rs149908314 | T | C | 8.634 | 1.919 | 7.01E-06 | -0.0025 | 0.0524 | 0.9619 |
|  | rs150156077 | G | A | 4.047 | 0.8896 | 5.56E-06 | -0.0237 | 0.0684 | 0.7294 |
|  | rs150400236 | T | C | 8.214 | 1.748 | 2.70E-06 | 0.0238 | 0.0797 | 0.7651 |
|  | rs150885241 | A | G | 31.1 | 3.924 | 3.08E-15 | -0.0525 | 0.0553 | 0.3421 |
|  | rs151244510 | A | G | 10.23 | 2.22 | 4.19E-06 | -0.0286 | 0.0411 | 0.4859 |
|  | rs16864501 | A | G | 33.31 | 5.744 | 7.31E-09 | -0.167 | 0.1713 | 0.3298 |
|  | rs17524895 | T | C | 4.477 | 0.9611 | 3.30E-06 | 0.0155 | 0.0357 | 0.6645 |
|  | rs17642570 | C | T | 3.649 | 0.7857 | 3.54E-06 | 0.0964 | 0.0978 | 0.3243 |
|  | rs17791728 | C | T | 15.79 | 2.811 | 2.10E-08 | -0.025 | 0.0425 | 0.5571 |
|  | rs17878699 | A | G | 8.176 | 1.62 | 4.73E-07 | 0.046 | 0.06 | 0.4435 |
|  | rs180697858 | G | A | 9.438 | 2.031 | 3.51E-06 | 0.0842 | 0.07 | 0.229 |
|  | rs180927720 | G | A | 11.34 | 2.525 | 7.39E-06 | 0.0348 | 0.108 | 0.7472 |
|  | rs180972485 | A | G | 12.36 | 2.205 | 2.22E-08 | -0.0031 | 0.0589 | 0.9581 |
|  | rs184040186 | A | T | 10.18 | 2.081 | 1.05E-06 | -0.0622 | 0.1843 | 0.7358 |
|  | rs185587352 | A | G | 18.17 | 3.072 | 3.62E-09 | -0.1964 | 0.0951 | 0.03894 |
|  | rs185754925 | G | A | 8.527 | 1.56 | 4.90E-08 | 0.0563 | 0.0533 | 0.2913 |
|  | rs186251573 | C | A | 14.06 | 2.974 | 2.35E-06 | 0.0533 | 0.0706 | 0.4508 |
|  | rs1879612 | C | T | -0.6741 | 0.1475 | 5.05E-06 | -0.0093 | 0.0188 | 0.6191 |
|  | rs188074116 | A | G | 22.71 | 4.723 | 1.59E-06 | 0.0832 | 0.0993 | 0.4017 |
|  | rs188087675 | A | G | 7.213 | 1.516 | 2.04E-06 | -0.1067 | 0.0751 | 0.1553 |
|  | rs189412340 | T | C | 16.05 | 3.457 | 3.59E-06 | -0.0787 | 0.0654 | 0.2286 |
|  | rs189796120 | T | C | 4.47 | 0.763 | 5.14E-09 | 0.0331 | 0.1814 | 0.855 |
|  | rs190658660 | A | T | 6.819 | 1.504 | 6.03E-06 | 0.055 | 0.052 | 0.29 |
|  | rs190665193 | G | A | 35.37 | 4.058 | 4.44E-18 | 0.0224 | 0.0375 | 0.5506 |
|  | rs190764862 | T | C | 19.5 | 4.4 | 9.65E-06 | -0.0829 | 0.0452 | 0.06687 |
|  | rs191607214 | A | C | 12.67 | 2.608 | 1.23E-06 | -0.038 | 0.175 | 0.828 |
|  | rs193135177 | A | G | 14.59 | 2.874 | 4.08E-07 | 0.0416 | 0.0851 | 0.624901 |
|  | rs200198801 | A | G | 8.758 | 1.857 | 2.50E-06 | 0.0256 | 0.0759 | 0.7357 |
|  | rs202047604 | C | A | 15.6 | 3.164 | 8.58E-07 | -0.0195 | 0.0255 | 0.4458 |
|  | rs2152857 | G | A | 4.942 | 0.9868 | 5.77E-07 | -0.0226 | 0.0462 | 0.6246 |
|  | rs2192500 | G | A | 5.306 | 1.184 | 7.66E-06 | -0.0851 | 0.1275 | 0.5049 |
|  | rs34039708 | A | C | 12.51 | 1.85 | 1.58E-11 | -0.0223 | 0.0715 | 0.755099 |
|  | rs34947488 | C | G | 1.023 | 0.2179 | 2.80E-06 | -0.0104 | 0.0262 | 0.6901 |
|  | rs35068665 | G | T | 53.41 | 5.65 | 5.87E-21 | 0.0835 | 0.0481 | 0.082459 |
|  | rs35772393 | T | C | 38.7 | 6.463 | 2.34E-09 | 0.016 | 0.0725 | 0.825 |
|  | rs374964617 | C | A | 6.492 | 1.196 | 6.12E-08 | -0.0592 | 0.0556 | 0.2874 |
|  | rs375356899 | G | A | 4.521 | 0.8043 | 2.04E-08 | -0.0198 | 0.0644 | 0.758799 |
|  | rs41276984 | G | C | 15.14 | 2.254 | 2.20E-11 | 0.0644 | 0.0688 | 0.3491 |
|  | rs449647 | T | A | 0.8492 | 0.1799 | 2.44E-06 | -0.021 | 0.0269 | 0.4352 |
|  | rs4670265 | G | T | 0.773 | 0.1507 | 3.07E-07 | 0.0234 | 0.0183 | 0.202 |
|  | rs4796089 | C | T | -1.046 | 0.2225 | 2.70E-06 | -0.042 | 0.0316 | 0.1835 |
|  | rs4853078 | G | T | 9.274 | 1.681 | 3.74E-08 | 0.1618 | 0.0502 | 0.001283 |
|  | rs4919341 | T | A | 1.034 | 0.2034 | 3.90E-07 | -0.0185 | 0.0255 | 0.47 |
|  | rs559632285 | A | C | 3.047 | 0.6636 | 4.55E-06 | -0.0345 | 0.0508 | 0.4967 |
|  | rs59615051 | C | T | 3.355 | 0.6746 | 6.91E-07 | -0.0232 | 0.0397 | 0.559099 |
|  | rs59745713 | A | G | 3.978 | 0.8973 | 9.60E-06 | -0.1628 | 0.1288 | 0.2061 |
|  | rs61578886 | T | C | 28.15 | 6.045 | 3.34E-06 | -0.0303 | 0.0397 | 0.4451 |
|  | rs61746209 | C | G | 1.998 | 0.391 | 3.39E-07 | 0.1114 | 0.0673 | 0.098039 |
|  | rs61759531 | G | C | 2.632 | 0.5897 | 8.32E-06 | -0.0508 | 0.0935 | 0.587 |
|  | rs62218442 | A | T | 15.67 | 2.862 | 4.65E-08 | -0.0256 | 0.0497 | 0.607 |
|  | rs62321187 | T | C | 49 | 5.703 | 1.26E-17 | 0.0058 | 0.1 | 0.9537 |
|  | rs62491100 | T | C | 5.693 | 1.172 | 1.24E-06 | 0.0564 | 0.0768 | 0.4626 |
|  | rs6982554 | C | G | 3.632 | 0.8104 | 7.66E-06 | 0.1567 | 0.172 | 0.3622 |
|  | rs7044904 | G | A | -14.67 | 3.168 | 3.78E-06 | 0.0927 | 0.1016 | 0.3614 |
|  | rs71528265 | C | G | 4.332 | 0.9119 | 2.11E-06 | 0.004 | 0.0474 | 0.933 |
|  | rs72719812 | G | A | 13.01 | 2.45 | 1.16E-07 | -0.0382 | 0.0374 | 0.307 |
|  | rs72723467 | A | G | 27.24 | 5.333 | 3.44E-07 | 0.0458 | 0.0931 | 0.6227 |
|  | rs72758450 | C | T | 33.05 | 5.644 | 5.19E-09 | 0.0173 | 0.0724 | 0.8107 |
|  | rs72931475 | G | A | 31.04 | 4.31 | 7.30E-13 | 0.0311 | 0.0779 | 0.6892 |
|  | rs73105597 | G | C | 8.765 | 1.905 | 4.36E-06 | 0.0687 | 0.0393 | 0.08021 |
|  | rs73129374 | A | G | 5.971 | 1.268 | 2.60E-06 | -0.2426 | 0.1363 | 0.07509 |
|  | rs73173143 | C | T | 1.87 | 0.4185 | 8.16E-06 | -0.0167 | 0.0379 | 0.6599 |
|  | rs73218592 | T | C | 10.01 | 2.227 | 7.23E-06 | -0.0263 | 0.0829 | 0.7515 |
|  | rs73400437 | T | C | 15.37 | 3.316 | 3.72E-06 | 0.0606 | 0.1092 | 0.578699 |
|  | rs73719023 | C | G | 3.222 | 0.5907 | 5.25E-08 | 0.0011 | 0.1151 | 0.9925 |
|  | rs73956020 | G | C | 92.8 | 17.04 | 5.57E-08 | -0.016 | 0.0729 | 0.826 |
|  | rs74157741 | T | C | 10.21 | 2.298 | 9.19E-06 | 0.0337 | 0.1339 | 0.8013 |
|  | rs74828441 | C | A | 3.976 | 0.8718 | 5.29E-06 | 0.0127 | 0.0781 | 0.8706 |
|  | rs75146333 | C | G | 37.39 | 7.091 | 1.43E-07 | -0.0581 | 0.0859 | 0.499001 |
|  | rs75438005 | A | G | 38.95 | 5.695 | 9.31E-12 | 0.0526 | 0.0673 | 0.4347 |
|  | rs75600477 | G | T | 13.07 | 2.441 | 9.25E-08 | 0.0557 | 0.0768 | 0.4682 |
|  | rs75772839 | T | C | 14.87 | 2.792 | 1.06E-07 | -0.2148 | 0.2664 | 0.4201 |
|  | rs75993384 | C | T | 38.22 | 4.737 | 9.73E-16 | -0.0326 | 0.0623 | 0.6009 |
|  | rs76128446 | G | A | 0.9087 | 0.2045 | 9.10E-06 | -0.0918 | 0.0389 | 0.01834 |
|  | rs76337573 | G | A | 5.366 | 1.027 | 1.85E-07 | 0.0021 | 0.0501 | 0.9662 |
|  | rs76444609 | A | G | 51.58 | 5.751 | 4.82E-19 | -0.044 | 0.0589 | 0.4552 |
|  | rs76552842 | A | G | 49.64 | 5.557 | 6.60E-19 | -0.1001 | 0.0831 | 0.2283 |
|  | rs76602542 | T | C | 9.611 | 2.144 | 7.59E-06 | -0.1077 | 0.0641 | 0.092901 |
|  | rs76942041 | A | G | 6.779 | 1.373 | 8.28E-07 | 0.0404 | 0.0615 | 0.5111 |
|  | rs77113660 | G | A | 52.91 | 5.64 | 1.16E-20 | -0.0033 | 0.072 | 0.9636 |
|  | rs77465871 | G | A | 13.03 | 1.94 | 2.13E-11 | 0.023 | 0.0553 | 0.677699 |
|  | rs77467380 | T | C | 34.38 | 5.85 | 4.56E-09 | -0.1051 | 0.0604 | 0.081969 |
|  | rs77676367 | C | T | 13.71 | 2.962 | 3.80E-06 | -0.183 | 0.1891 | 0.3331 |
|  | rs77689186 | A | C | 7.95 | 1.654 | 1.60E-06 | -0.0347 | 0.1055 | 0.7426 |
|  | rs78069205 | G | A | 46.68 | 7.967 | 5.07E-09 | -0.1734 | 0.1277 | 0.1746 |
|  | rs78088167 | G | A | 19.81 | 3.224 | 8.99E-10 | 0.0584 | 0.0692 | 0.3991 |
|  | rs7823439 | C | T | 5.545 | 1.177 | 2.54E-06 | 0.0215 | 0.0649 | 0.7402 |
|  | rs78470553 | A | G | 3.909 | 0.7324 | 1.01E-07 | -0.0199 | 0.0292 | 0.4943 |
|  | rs78750136 | C | T | 100.8 | 11.59 | 5.21E-18 | -0.1154 | 0.0551 | 0.03638 |
|  | rs78922116 | T | C | 6.968 | 1.353 | 2.73E-07 | -0.1097 | 0.0771 | 0.1549 |
|  | rs79027575 | C | T | 9.036 | 1.652 | 4.84E-08 | 0.0091 | 0.0913 | 0.9203 |
|  | rs79218577 | C | G | 15.3 | 3.059 | 5.93E-07 | 0.0377 | 0.0554 | 0.4965 |
|  | rs79290306 | A | G | 6.88 | 1.357 | 4.22E-07 | 0.127 | 0.0563 | 0.02406 |
|  | rs79395509 | T | C | 26.23 | 4.191 | 4.38E-10 | 0.3292 | 0.2015 | 0.1023 |
|  | rs79518488 | C | T | 24.55 | 4.474 | 4.37E-08 | -0.1003 | 0.1149 | 0.3828 |
|  | rs79647440 | T | C | 37.38 | 4.922 | 3.94E-14 | -0.0273 | 0.0613 | 0.6557 |
|  | rs79725470 | C | T | 19.78 | 3.684 | 8.40E-08 | 0.2745 | 0.0754 | 0.000274 |
|  | rs79851411 | A | G | 35.15 | 5.793 | 1.44E-09 | -0.0364 | 0.0547 | 0.5055 |
|  | rs80181351 | T | C | 7.334 | 1.616 | 5.87E-06 | 0.0453 | 0.0975 | 0.6422 |
|  | rs80331554 | G | A | 44.6 | 7.703 | 7.70E-09 | 0.0104 | 0.0705 | 0.8829 |
|  | rs8040007 | C | G | 0.7129 | 0.1611 | 9.92E-06 | 0.0364 | 0.0233 | 0.1184 |
|  | rs8142081 | A | G | 1.373 | 0.2961 | 3.68E-06 | 0.0109 | 0.0488 | 0.8233 |
|  | rs9408992 | G | C | 5.704 | 1.27 | 7.29E-06 | -0.156 | 0.0888 | 0.07883 |
| CD20 on IgD+ CD24+ | rs114915558 | G | A | 0.305 | 3.46E-06 | 0.06562 | -0.0105 | 0.0463 | 0.8209 |
|  | rs11662753 | G | C | 0.1706 | 9.48E-06 | 0.03846 | -0.0352 | 0.029 | 0.2252 |
|  | rs116729050 | A | G | -0.2379 | 5.17E-06 | 0.05211 | -0.1225 | 0.1086 | 0.2592 |
|  | rs13207893 | C | T | 0.1399 | 1.95E-06 | 0.02935 | -0.0564 | 0.036 | 0.1176 |
|  | rs149204679 | G | A | -1.317 | 5.97E-06 | 0.2905 | 0.0258 | 0.1075 | 0.8102 |
|  | rs149214933 | G | T | -0.7345 | 7.24E-06 | 0.1635 | 0.0942 | 0.0454 | 0.038 |
|  | rs1569590 | G | A | 0.2082 | 1.85E-08 | 0.03692 | -0.0199 | 0.0284 | 0.4837 |
|  | rs1941033 | T | C | -0.2221 | 9.60E-08 | 0.04155 | 0.0684 | 0.0447 | 0.1265 |
|  | rs2532969 | T | C | 0.1154 | 7.80E-06 | 0.02577 | 0.0341 | 0.0183 | 0.06276 |
|  | rs28572463 | C | G | 0.2436 | 2.90E-07 | 0.0474 | 0.0907 | 0.3026 | 0.764401 |
|  | rs3129750 | A | G | -0.1603 | 2.96E-06 | 0.03424 | -3.00E-04 | 0.0261 | 0.9912 |
|  | rs35525697 | T | A | 0.114 | 4.96E-06 | 0.02492 | -0.0265 | 0.0184 | 0.1504 |
|  | rs4746507 | A | C | 0.1293 | 9.57E-07 | 0.02634 | -0.0485 | 0.0201 | 0.01566 |
|  | rs55809481 | C | T | -0.2125 | 1.61E-13 | 0.0287 | 0.027 | 0.0199 | 0.1743 |
|  | rs61731791 | T | C | 0.2089 | 7.15E-07 | 0.04207 | 0.0666 | 0.0555 | 0.2305 |
|  | rs62000215 | T | C | -0.8854 | 1.33E-06 | 0.1828 | -0.0127 | 0.0458 | 0.7812 |
|  | rs62340965 | A | T | -0.1558 | 6.08E-07 | 0.03118 | 0.0151 | 0.0341 | 0.6574 |
|  | rs62623429 | T | C | 0.6367 | 8.46E-06 | 0.1428 | -0.0696 | 0.0535 | 0.1933 |
|  | rs66637059 | T | C | -0.1563 | 8.74E-06 | 0.03511 | -0.0157 | 0.0255 | 0.537 |
|  | rs7128035 | C | T | -0.1284 | 2.42E-06 | 0.02718 | 0.0234 | 0.0212 | 0.2702 |
|  | rs76239564 | G | A | 0.6515 | 6.05E-06 | 0.1438 | 0.0132 | 0.0624 | 0.832 |
|  | rs77121649 | T | C | 0.2352 | 6.34E-07 | 0.04714 | 0.1829 | 0.1311 | 0.163 |
|  | rs79110866 | G | A | 0.2849 | 3.79E-06 | 0.06153 | -0.0265 | 0.0777 | 0.733199 |
|  | rs9461562 | G | A | 0.2717 | 6.65E-08 | 0.0502 | -0.0052 | 0.0767 | 0.9458 |
| CD20 on IgD- CD24- | rs10171238 | T | C | 0.1442 | 0.0302 | 1.86E-06 | -0.0056 | 0.021 | 0.7899 |
|  | rs11051833 | T | C | -0.3389 | 0.07414 | 5.03E-06 | -1.00E-04 | 0.031 | 0.9973 |
|  | rs11751198 | A | G | 0.1933 | 0.03626 | 1.03E-07 | 0.0799 | 0.0972 | 0.4112 |
|  | rs117961939 | T | C | -0.3034 | 0.06719 | 6.50E-06 | 0.0705 | 0.1304 | 0.5891 |
|  | rs12660382 | T | C | 0.1358 | 0.02903 | 3.00E-06 | -0.0113 | 0.0255 | 0.6591 |
|  | rs13181744 | C | T | -0.1152 | 0.02545 | 6.17E-06 | 0.0086 | 0.019 | 0.6497 |
|  | rs13381717 | T | A | -0.2077 | 0.04625 | 7.34E-06 | 0.0206 | 0.0407 | 0.613501 |
|  | rs139713744 | T | C | 1.747 | 0.3908 | 8.09E-06 | -0.0339 | 0.0762 | 0.656299 |
|  | rs1453509 | A | C | -0.1691 | 0.03813 | 9.53E-06 | -0.002 | 0.0213 | 0.9265 |
|  | rs17136624 | A | G | -0.1293 | 0.02835 | 5.30E-06 | 0.0324 | 0.0227 | 0.1546 |
|  | rs1838178 | G | A | 0.1146 | 0.02554 | 7.40E-06 | -5.00E-04 | 0.0194 | 0.9799 |
|  | rs30006 | C | T | -0.196 | 0.02941 | 3.03E-11 | 0.0267 | 0.0199 | 0.18 |
|  | rs35485457 | T | G | -0.1404 | 0.03173 | 9.94E-06 | 0.0011 | 0.0208 | 0.9595 |
|  | rs35567463 | A | C | 0.1709 | 0.02875 | 3.02E-09 | 0.0181 | 0.0337 | 0.5914 |
|  | rs4426778 | A | G | -0.1237 | 0.02524 | 9.87E-07 | 0.0067 | 0.0182 | 0.7117 |
|  | rs61731791 | T | C | 0.2019 | 0.04254 | 2.15E-06 | 0.0666 | 0.0555 | 0.2305 |
|  | rs62299189 | C | A | -0.2121 | 0.04588 | 3.90E-06 | 0.0327 | 0.0307 | 0.2879 |
|  | rs6454789 | T | C | 0.1147 | 0.02581 | 9.14E-06 | 0.026 | 0.0189 | 0.1697 |
|  | rs73196454 | A | G | -1.601 | 0.3586 | 8.24E-06 | 0.081 | 0.0663 | 0.2221 |
|  | rs74435765 | T | C | -0.3934 | 0.08597 | 4.89E-06 | 0.0335 | 0.1041 | 0.747901 |
|  | rs77463781 | C | G | 0.1693 | 0.03696 | 4.80E-06 | 0.0628 | 0.0626 | 0.3161 |
|  | rs7754613 | T | C | 0.1526 | 0.03238 | 2.52E-06 | -0.0205 | 0.0238 | 0.3893 |
|  | rs78287249 | T | G | -0.1865 | 0.04132 | 6.55E-06 | 0.0749 | 0.039 | 0.054739 |
|  | rs78373541 | G | A | 0.2667 | 0.05821 | 4.78E-06 | -0.0596 | 0.0461 | 0.1958 |
|  | rs787045 | C | G | -0.1629 | 0.03657 | 8.70E-06 | 0.0474 | 0.0292 | 0.1043 |
|  | rs849532 | G | A | 0.3351 | 0.0616 | 5.70E-08 | -0.0365 | 0.043 | 0.3955 |
| CD24 on IgD+ CD38br | rs10056811 | A | G | -0.1322 | 0.02752 | 1.62E-06 | 0.0065 | 0.019 | 0.733301 |
|  | rs10108273 | A | T | 0.1701 | 0.0373 | 5.28E-06 | -0.0175 | 0.0226 | 0.4386 |
|  | rs114151424 | A | G | 1.159 | 0.2197 | 1.40E-07 | -0.045 | 0.0353 | 0.2022 |
|  | rs116032501 | C | A | -0.1879 | 0.04125 | 5.42E-06 | 0.0282 | 0.0515 | 0.584501 |
|  | rs117920360 | T | C | 0.1459 | 0.03267 | 8.28E-06 | 0.0625 | 0.0423 | 0.139 |
|  | rs139160176 | C | G | -0.9719 | 0.2023 | 1.61E-06 | 0.2573 | 0.153 | 0.092619 |
|  | rs146379662 | T | C | 0.5365 | 0.1154 | 3.47E-06 | -0.0915 | 0.0984 | 0.3522 |
|  | rs16923647 | T | C | -0.164 | 0.03428 | 1.79E-06 | 0.0118 | 0.0243 | 0.6261 |
|  | rs1799693 | G | A | -0.2018 | 0.03253 | 6.12E-10 | 0.0499 | 0.0364 | 0.1711 |
|  | rs181036992 | C | T | -0.4375 | 0.091 | 1.59E-06 | 0.0348 | 0.0594 | 0.5575 |
|  | rs192325628 | T | C | 0.4876 | 0.09848 | 7.70E-07 | 0.1337 | 0.1547 | 0.3874 |
|  | rs3097667 | C | G | -0.1519 | 0.03161 | 1.61E-06 | 0.0329 | 0.0465 | 0.4793 |
|  | rs35093039 | A | G | -0.21 | 0.04748 | 9.99E-06 | 0.034 | 0.0246 | 0.1666 |
|  | rs4368221 | C | G | 0.1558 | 0.03385 | 4.33E-06 | 0.0054 | 0.0209 | 0.7953 |
|  | rs45609135 | A | G | -0.1767 | 0.03959 | 8.31E-06 | 0.0074 | 0.033 | 0.8233 |
|  | rs4765854 | C | A | -0.2274 | 0.05027 | 6.26E-06 | -0.0169 | 0.031 | 0.5848 |
|  | rs57680652 | T | C | 0.128 | 0.02606 | 9.41E-07 | 0.0353 | 0.0188 | 0.06022 |
|  | rs59655785 | G | A | 0.1174 | 0.02474 | 2.18E-06 | 0.0385 | 0.0205 | 0.05996 |
|  | rs6666130 | T | G | -0.2436 | 0.05141 | 2.25E-06 | 0.0327 | 0.0694 | 0.637801 |
|  | rs75059545 | C | A | 0.1761 | 0.03786 | 3.39E-06 | -0.0337 | 0.0263 | 0.2002 |
|  | rs7781145 | A | C | -0.1421 | 0.0291 | 1.09E-06 | 7.00E-04 | 0.0232 | 0.9776 |
|  | rs78405192 | C | T | 1.577 | 0.3497 | 6.72E-06 | -0.1071 | 0.052 | 0.03933 |
|  | rs9326867 | G | T | 0.1697 | 0.03713 | 5.04E-06 | 0.0062 | 0.024 | 0.7958 |
|  | rs9398107 | G | A | -0.4386 | 0.0546 | 1.28E-15 | -0.0153 | 0.0529 | 0.7722 |
|  | rs9452529 | T | C | -0.5098 | 0.1073 | 2.09E-06 | 0.0293 | 0.0272 | 0.2822 |
| CD62L on CD62L+ DC | rs10435954 | T | C | -0.1261 | 0.0266 | 2.21E-06 | 0.0099 | 0.0189 | 0.5987 |
|  | rs11029935 | C | T | -0.3486 | 0.07863 | 9.61E-06 | 0.085 | 0.0394 | 0.03086 |
|  | rs112295585 | T | C | -1.243 | 0.2597 | 1.79E-06 | 0.0272 | 0.0534 | 0.6107 |
|  | rs113947601 | T | C | 0.331 | 0.07451 | 9.24E-06 | 0.0176 | 0.0368 | 0.6324 |
|  | rs115980202 | C | G | 0.3845 | 0.08218 | 3.03E-06 | -0.0174 | 0.0991 | 0.8608 |
|  | rs11692722 | G | A | -0.1277 | 0.02811 | 5.78E-06 | 0.03 | 0.0239 | 0.2088 |
|  | rs11773763 | T | C | -0.1607 | 0.03014 | 1.04E-07 | 0.0318 | 0.0196 | 0.1049 |
|  | rs12040885 | A | G | -0.1219 | 0.02617 | 3.32E-06 | 0.0027 | 0.0182 | 0.8822 |
|  | rs12041883 | A | C | -0.2019 | 0.0393 | 2.97E-07 | -0.0293 | 0.0237 | 0.2169 |
|  | rs12864970 | A | C | 0.1155 | 0.02591 | 8.55E-06 | 0.0046 | 0.0183 | 0.8031 |
|  | rs133635 | C | T | 0.2292 | 0.05142 | 8.63E-06 | -0.0103 | 0.0337 | 0.759299 |
|  | rs140597204 | T | C | -0.4709 | 0.09393 | 5.68E-07 | -0.1677 | 0.1589 | 0.2911 |
|  | rs149982484 | T | C | -0.2888 | 0.06366 | 5.97E-06 | -0.009 | 0.0686 | 0.8957 |
|  | rs150038452 | C | G | 0.4093 | 0.0837 | 1.06E-06 | 0.0249 | 0.0574 | 0.6642 |
|  | rs2428305 | G | A | 0.2654 | 0.05112 | 2.22E-07 | -0.0148 | 0.0263 | 0.5751 |
|  | rs35443755 | C | G | -0.5143 | 0.1159 | 9.45E-06 | -0.0116 | 0.0696 | 0.868 |
|  | rs3917768 | G | T | 0.1797 | 0.02746 | 7.06E-11 | -0.0389 | 0.0185 | 0.03514 |
|  | rs4731265 | A | G | 0.1235 | 0.02738 | 6.74E-06 | -0.0091 | 0.019 | 0.6303 |
|  | rs58315932 | A | G | 0.3204 | 0.07039 | 5.54E-06 | -0.0207 | 0.0405 | 0.6096 |
|  | rs62236308 | T | G | -0.1633 | 0.03376 | 1.39E-06 | -5.00E-04 | 0.0273 | 0.9847 |
|  | rs703648 | C | T | 0.2647 | 0.05566 | 2.08E-06 | -0.0495 | 0.073 | 0.498 |
|  | rs72997833 | A | G | 0.1594 | 0.03415 | 3.18E-06 | -0.0233 | 0.0218 | 0.2847 |
|  | rs742800 | T | G | 0.2642 | 0.05264 | 5.51E-07 | -0.0113 | 0.0318 | 0.7213 |
|  | rs7526645 | T | C | 0.1721 | 0.02612 | 5.33E-11 | -0.0432 | 0.019 | 0.02262 |
|  | rs75307154 | T | C | -0.306 | 0.06553 | 3.16E-06 | -0.0379 | 0.0253 | 0.1343 |
|  | rs75943000 | C | G | 0.2084 | 0.04232 | 8.94E-07 | -0.0177 | 0.0226 | 0.4333 |
| CD3 on Naive CD4+ | rs112171230 | T | C | 0.3132 | 3.32E-07 | 0.06122 | -0.0196 | 0.0299 | 0.511601 |
|  | rs113208949 | T | C | 0.6287 | 1.06E-06 | 0.1285 | -0.0793 | 0.099 | 0.4228 |
|  | rs113731203 | C | T | -0.4916 | 4.11E-06 | 0.1065 | 0.0159 | 0.0863 | 0.854 |
|  | rs11715223 | A | G | 0.1539 | 3.90E-06 | 0.03327 | -0.0032 | 0.0203 | 0.8748 |
|  | rs118065562 | A | G | -0.6044 | 3.92E-06 | 0.1307 | -0.0918 | 0.074 | 0.215 |
|  | rs12502302 | T | C | 0.1301 | 2.86E-06 | 0.02774 | -0.0155 | 0.0206 | 0.452 |
|  | rs139257983 | G | A | -0.332 | 7.19E-06 | 0.07384 | 0.054 | 0.089 | 0.544 |
|  | rs141784254 | G | A | -1.116 | 2.12E-07 | 0.2145 | 0.0853 | 0.068 | 0.2096 |
|  | rs146330992 | T | C | 0.2517 | 5.05E-07 | 0.04997 | 0.05 | 0.0792 | 0.5278 |
|  | rs146993166 | T | C | 0.2723 | 9.06E-06 | 0.06124 | 0.0446 | 0.0682 | 0.5132 |
|  | rs2003830 | G | C | -0.2093 | 4.45E-10 | 0.03343 | -0.0306 | 0.0184 | 0.097089 |
|  | rs2213429 | T | C | -0.1619 | 3.38E-06 | 0.03478 | -2.00E-04 | 0.0246 | 0.9951 |
|  | rs2413113 | C | T | 0.13 | 1.91E-06 | 0.02724 | 0.0164 | 0.0184 | 0.3707 |
|  | rs28535434 | C | T | 0.1895 | 4.21E-06 | 0.04111 | 0.0022 | 0.0208 | 0.9146 |
|  | rs28607988 | C | A | 0.2334 | 1.46E-10 | 0.03629 | 0.0173 | 0.031 | 0.5774 |
|  | rs2949661 | T | C | 0.6864 | 5.66E-154 | 0.02442 | -0.0351 | 0.0207 | 0.090511 |
|  | rs34064330 | G | T | -0.1285 | 9.55E-06 | 0.02897 | -0.0297 | 0.0183 | 0.1046 |
|  | rs4396110 | A | G | -0.1303 | 2.09E-06 | 0.0274 | -0.0222 | 0.0206 | 0.2816 |
|  | rs4657649 | C | T | 0.5489 | 3.34E-19 | 0.06086 | -0.0146 | 0.0292 | 0.6169 |
|  | rs4925475 | C | T | 0.1214 | 9.54E-06 | 0.02737 | -0.0253 | 0.0187 | 0.1769 |
|  | rs61814872 | T | C | 0.3354 | 4.48E-14 | 0.04422 | -0.0211 | 0.03 | 0.4824 |
|  | rs62035375 | C | G | 0.1419 | 1.10E-06 | 0.02906 | 0.0435 | 0.0186 | 0.01909 |
|  | rs62232208 | G | A | -0.1325 | 1.53E-06 | 0.02751 | 8.00E-04 | 0.0208 | 0.9689 |
|  | rs62465786 | T | C | 1.065 | 6.83E-06 | 0.2364 | 0.0017 | 0.0393 | 0.9654 |
|  | rs74620952 | C | T | -1.343 | 5.25E-08 | 0.246 | 0.1409 | 0.0579 | 0.0149 |
|  | rs74744824 | G | A | 0.3526 | 8.22E-06 | 0.07893 | -0.0312 | 0.0542 | 0.564901 |
|  | rs7502332 | A | G | -0.1403 | 8.25E-07 | 0.0284 | 0.0046 | 0.0188 | 0.8078 |
|  | rs7530127 | C | T | -0.1373 | 8.24E-07 | 0.0278 | 0.0072 | 0.0199 | 0.7173 |
|  | rs76066554 | T | C | -1.041 | 1.58E-06 | 0.2163 | -0.1487 | 0.1998 | 0.4566 |
|  | rs9318267 | G | T | 0.1354 | 6.71E-06 | 0.03002 | -0.0272 | 0.0195 | 0.1634 |
| CD3 on EM CD4+ | rs115265205 | A | G | 1.579 | 0.3329 | 2.20E-06 | -0.0481 | 0.0523 | 0.3582 |
|  | rs150528455 | A | G | -0.3078 | 0.06611 | 3.38E-06 | -0.0464 | 0.0852 | 0.5857 |
|  | rs2244434 | A | G | -0.171 | 0.0342 | 6.08E-07 | 0.0098 | 0.0193 | 0.612999 |
|  | rs226503 | T | G | -0.1987 | 0.04321 | 4.43E-06 | 0.0516 | 0.0241 | 0.03274 |
|  | rs28649534 | T | C | 0.2174 | 0.04693 | 3.77E-06 | 0.0214 | 0.0418 | 0.608199 |
|  | rs2995089 | A | G | 0.4829 | 0.02629 | 2.05E-71 | -0.0317 | 0.0207 | 0.1266 |
|  | rs4288797 | A | C | -0.5981 | 0.1239 | 1.45E-06 | 0.0602 | 0.1341 | 0.6536 |
|  | rs4514905 | G | A | -0.1403 | 0.028 | 5.72E-07 | -3.00E-04 | 0.0187 | 0.9886 |
|  | rs4656440 | G | C | -0.3205 | 0.06356 | 4.87E-07 | 0.0383 | 0.0345 | 0.2672 |
|  | rs4657649 | C | T | 0.3343 | 0.06234 | 8.87E-08 | -0.0146 | 0.0292 | 0.6169 |
|  | rs62028293 | C | T | 0.2449 | 0.05279 | 3.64E-06 | 0.0221 | 0.0655 | 0.7361 |
|  | rs62046402 | A | C | 0.3895 | 0.083 | 2.81E-06 | -0.0544 | 0.0411 | 0.1858 |
|  | rs6684311 | G | C | -0.2348 | 0.03907 | 2.09E-09 | 0.0304 | 0.0211 | 0.1502 |
|  | rs727908 | C | G | -0.1394 | 0.03119 | 8.13E-06 | -0.0038 | 0.0196 | 0.8455 |
|  | rs72940579 | T | C | -0.14 | 0.03143 | 8.73E-06 | -0.0069 | 0.0202 | 0.7348 |
|  | rs73068237 | C | T | 1.929 | 0.3967 | 1.23E-06 | -0.0309 | 0.2309 | 0.8935 |
|  | rs74139031 | G | A | -0.6324 | 0.1424 | 9.37E-06 | -0.0725 | 0.0486 | 0.1361 |
|  | rs7519927 | A | G | -0.1522 | 0.02803 | 6.16E-08 | 0.0019 | 0.0188 | 0.9214 |
|  | rs7595804 | C | T | -0.2313 | 0.05202 | 9.04E-06 | 0.0582 | 0.0315 | 0.06488 |
| CD3 on HLA DR+ CD4+ | rs10195369 | A | G | -0.2203 | 7.74E-06 | 0.04916 | 0.0149 | 0.0286 | 0.604 |
|  | rs10753692 | T | C | 0.1242 | 5.27E-06 | 0.02722 | -0.0212 | 0.0199 | 0.287 |
|  | rs10933796 | A | G | 0.1235 | 9.69E-06 | 0.02787 | -3.00E-04 | 0.0192 | 0.988 |
|  | rs11042750 | T | C | 0.1283 | 3.78E-06 | 0.02769 | -0.0114 | 0.0184 | 0.5374 |
|  | rs113710453 | A | G | 0.2699 | 6.35E-06 | 0.05969 | -0.0064 | 0.0363 | 0.8592 |
|  | rs114102310 | A | T | -0.1872 | 5.22E-06 | 0.04102 | -0.0643 | 0.0531 | 0.2254 |
|  | rs12596540 | G | A | 0.1435 | 2.98E-06 | 0.03065 | 0.0011 | 0.0212 | 0.9601 |
|  | rs13072593 | G | A | 0.1445 | 3.85E-06 | 0.03122 | 0.0194 | 0.0207 | 0.3509 |
|  | rs13170201 | C | T | -0.1416 | 3.11E-06 | 0.03031 | 0.0052 | 0.0184 | 0.777499 |
|  | rs145799392 | T | C | -0.3383 | 1.96E-06 | 0.07098 | -0.0051 | 0.0475 | 0.9141 |
|  | rs147209445 | T | C | 0.5455 | 8.36E-06 | 0.1222 | -0.084 | 0.0588 | 0.1529 |
|  | rs16867696 | C | A | -0.3544 | 4.17E-07 | 0.06988 | 0.0604 | 0.0283 | 0.03317 |
|  | rs1723018 | G | A | 0.4384 | 1.01E-61 | 0.02585 | -0.0357 | 0.0205 | 0.08179 |
|  | rs188731571 | T | C | 0.7877 | 7.09E-06 | 0.1751 | 0.2544 | 0.2994 | 0.3955 |
|  | rs2010043 | A | C | 0.2196 | 3.47E-07 | 0.04299 | -0.0026 | 0.0261 | 0.9222 |
|  | rs27265 | T | C | 0.1941 | 2.28E-07 | 0.03743 | 0.0058 | 0.0273 | 0.8326 |
|  | rs2742060 | A | G | 0.1752 | 8.49E-07 | 0.03551 | -0.0192 | 0.0197 | 0.3292 |
|  | rs4787901 | G | T | 0.1293 | 4.11E-06 | 0.02802 | 0.0044 | 0.0192 | 0.8182 |
|  | rs55952666 | C | T | 0.2538 | 5.01E-06 | 0.05551 | 0.0142 | 0.023 | 0.5374 |
|  | rs59738549 | G | C | 0.3869 | 2.38E-10 | 0.06088 | 0.0674 | 0.055 | 0.2197 |
|  | rs6684311 | G | C | -0.2046 | 1.18E-07 | 0.03854 | 0.0304 | 0.0211 | 0.1502 |
|  | rs6765892 | T | C | 0.1716 | 4.45E-06 | 0.03733 | -0.0062 | 0.0301 | 0.8382 |
|  | rs73167431 | T | C | -1.73 | 2.56E-06 | 0.367 | 0.035 | 0.0896 | 0.6959 |
|  | rs75535842 | A | C | -0.2789 | 8.75E-07 | 0.05659 | -0.0053 | 0.0335 | 0.8745 |
|  | rs75771396 | C | T | -0.3015 | 4.43E-06 | 0.06557 | 0.0204 | 0.0289 | 0.481599 |
|  | rs9408777 | T | A | 0.1305 | 4.24E-06 | 0.02832 | -0.0075 | 0.0204 | 0.713801 |
| CD3 on HLA DR+ CD8br | rs11170960 | A | G | 0.3537 | 0.07979 | 9.61E-06 | -0.0313 | 0.0403 | 0.4385 |
|  | rs112025123 | C | T | -0.4918 | 0.1005 | 1.05E-06 | 0.1727 | 0.0542 | 0.001446 |
|  | rs11233873 | C | A | 0.2377 | 0.05106 | 3.39E-06 | -0.0452 | 0.0407 | 0.2673 |
|  | rs115825160 | A | C | -0.3658 | 0.08215 | 8.76E-06 | -0.0174 | 0.0366 | 0.6353 |
|  | rs11624277 | A | G | -1.165 | 0.2603 | 7.87E-06 | 0.1035 | 0.1173 | 0.3774 |
|  | rs116911862 | A | G | 1.012 | 0.2244 | 6.73E-06 | 0.0217 | 0.0475 | 0.6475 |
|  | rs12032130 | T | C | 0.143 | 0.02834 | 4.79E-07 | 0.0072 | 0.0188 | 0.702301 |
|  | rs12141641 | T | G | -0.1758 | 0.03928 | 7.93E-06 | -0.0272 | 0.0291 | 0.3494 |
|  | rs1773542 | C | T | 0.2805 | 0.0264 | 6.52E-26 | -0.036 | 0.0205 | 0.079219 |
|  | rs181802824 | G | A | -0.391 | 0.08208 | 1.99E-06 | 0.1528 | 0.2813 | 0.587 |
|  | rs2036009 | A | G | 0.6276 | 0.1326 | 2.30E-06 | -0.0274 | 0.0513 | 0.5931 |
|  | rs2863272 | A | G | 0.1701 | 0.03312 | 2.96E-07 | -0.0013 | 0.02 | 0.9478 |
|  | rs288415 | A | G | 0.2517 | 0.05506 | 5.03E-06 | 0.0651 | 0.0467 | 0.163 |
|  | rs4608913 | G | A | 0.2143 | 0.04812 | 8.78E-06 | -0.011 | 0.029 | 0.705801 |
|  | rs4668614 | A | G | 0.1269 | 0.02653 | 1.81E-06 | -0.0034 | 0.0184 | 0.8517 |
|  | rs4742559 | C | T | 0.279 | 0.06208 | 7.25E-06 | -0.0734 | 0.0397 | 0.064899 |
|  | rs56383740 | C | T | -0.5456 | 0.1231 | 9.74E-06 | 0.0569 | 0.0964 | 0.555 |
|  | rs57740770 | G | T | -0.1567 | 0.03481 | 6.96E-06 | -0.0113 | 0.1278 | 0.9298 |
|  | rs6498116 | T | A | 0.1434 | 0.03109 | 4.12E-06 | 8.00E-04 | 0.0212 | 0.9683 |
|  | rs73167431 | T | C | -1.655 | 0.3674 | 6.90E-06 | 0.035 | 0.0896 | 0.6959 |
|  | rs76442794 | G | A | -0.3516 | 0.07319 | 1.64E-06 | 4.00E-04 | 0.0577 | 0.9948 |
|  | rs7788673 | T | A | -0.1553 | 0.03223 | 1.51E-06 | 3.00E-04 | 0.0215 | 0.9889 |
|  | rs78539923 | A | C | 0.2869 | 0.06449 | 8.93E-06 | -0.0569 | 0.0406 | 0.1615 |
|  | rs79048891 | A | T | 0.1679 | 0.03715 | 6.42E-06 | -0.0057 | 0.0212 | 0.788 |
|  | rs8057798 | T | G | 0.1361 | 0.03032 | 7.42E-06 | -0.0141 | 0.0185 | 0.444 |
|  | rs864075 | T | C | -0.1558 | 0.03361 | 3.71E-06 | 0.0064 | 0.0205 | 0.756 |
| CD3 on secreting Treg | rs114672530 | A | G | -0.6777 | 1.29E-10 | 0.105 | 0.0274 | 0.0711 | 0.6996 |
|  | rs115839712 | A | T | -0.4329 | 7.54E-06 | 0.0965 | -0.2049 | 0.1718 | 0.2331 |
|  | rs116168222 | T | C | -0.4436 | 5.80E-06 | 0.09767 | -0.0698 | 0.0987 | 0.4794 |
|  | rs12884372 | G | T | 0.2567 | 9.42E-07 | 0.05224 | 0.0527 | 0.075 | 0.4822 |
|  | rs1324073 | C | G | -0.1502 | 2.18E-07 | 0.0289 | -0.0266 | 0.0266 | 0.3183 |
|  | rs142130759 | A | G | 0.6893 | 4.00E-07 | 0.1357 | 0.0134 | 0.0721 | 0.8527 |
|  | rs142935756 | G | A | -0.9409 | 2.18E-06 | 0.1983 | 0.0158 | 0.045 | 0.7248 |
|  | rs2949661 | T | C | 0.6149 | 4.33E-119 | 0.02529 | -0.0351 | 0.0207 | 0.090511 |
|  | rs4396110 | A | G | -0.1308 | 2.20E-06 | 0.02757 | -0.0222 | 0.0206 | 0.2816 |
|  | rs4656440 | G | C | -0.3547 | 2.39E-08 | 0.06338 | 0.0383 | 0.0345 | 0.2672 |
|  | rs4657649 | C | T | 0.3533 | 1.33E-08 | 0.062 | -0.0146 | 0.0292 | 0.6169 |
|  | rs4763361 | G | C | 1.75 | 8.38E-06 | 0.3921 | -0.1291 | 0.1176 | 0.2724 |
|  | rs55799444 | T | C | -0.131 | 8.26E-06 | 0.02933 | 0.0107 | 0.0189 | 0.5708 |
|  | rs57844740 | A | G | 0.3747 | 3.58E-06 | 0.0807 | -0.0199 | 0.044 | 0.6507 |
|  | rs6684311 | G | C | -0.2656 | 1.02E-11 | 0.03889 | 0.0304 | 0.0211 | 0.1502 |
|  | rs7094641 | A | G | 0.1386 | 9.36E-06 | 0.03123 | 0.0042 | 0.02 | 0.8347 |
|  | rs74620952 | C | T | -1.108 | 8.48E-06 | 0.2485 | 0.1409 | 0.0579 | 0.0149 |
|  | rs75466245 | C | T | 0.2643 | 8.24E-06 | 0.05917 | 0.0265 | 0.0282 | 0.3464 |
|  | rs78042544 | G | A | 0.4272 | 4.81E-06 | 0.09325 | -0.0068 | 0.0354 | 0.8474 |
|  | rs8024202 | A | G | -0.1356 | 1.18E-06 | 0.02784 | -0.0133 | 0.0204 | 0.5133 |
|  | rs869139 | C | T | -0.128 | 3.45E-06 | 0.02752 | 0.0016 | 0.0186 | 0.9324 |
| CD3 on CD39+ CD4+ | rs10483184 | T | G | -0.5745 | 9.51E-06 | 0.1295 | -0.0034 | 0.033 | 0.9188 |
|  | rs113842802 | T | C | 0.2248 | 5.03E-06 | 0.04917 | -0.0449 | 0.0384 | 0.2424 |
|  | rs117734680 | G | A | -1.156 | 3.22E-06 | 0.2478 | -0.0332 | 0.0549 | 0.5454 |
|  | rs12035159 | T | C | -0.1491 | 8.26E-06 | 0.03338 | 0.0169 | 0.0229 | 0.4598 |
|  | rs12077809 | A | C | -0.2013 | 1.43E-07 | 0.03817 | -0.0273 | 0.0255 | 0.2847 |
|  | rs12138291 | A | G | -0.2773 | 7.97E-13 | 0.03854 | 0.0299 | 0.0212 | 0.1575 |
|  | rs139257983 | G | A | -0.3386 | 5.08E-06 | 0.07409 | 0.054 | 0.089 | 0.544 |
|  | rs141328863 | T | C | 1.284 | 7.48E-06 | 0.2862 | 0.0525 | 0.0667 | 0.4315 |
|  | rs142664794 | T | C | 0.3573 | 7.32E-06 | 0.07954 | 0.0183 | 0.0314 | 0.56 |
|  | rs2700 | C | A | 0.1643 | 6.11E-06 | 0.03626 | -0.0491 | 0.0199 | 0.01356 |
|  | rs2949661 | T | C | 0.644 | 3.76E-132 | 0.02499 | -0.0351 | 0.0207 | 0.090511 |
|  | rs41434450 | A | G | -0.2405 | 2.52E-06 | 0.05099 | 0.0138 | 0.0371 | 0.711001 |
|  | rs4456952 | T | C | -0.3788 | 9.00E-06 | 0.08517 | 0.0699 | 0.0507 | 0.1678 |
|  | rs4656440 | G | C | -0.3594 | 1.47E-08 | 0.06326 | 0.0383 | 0.0345 | 0.2672 |
|  | rs4763361 | G | C | 1.745 | 8.24E-06 | 0.3906 | -0.1291 | 0.1176 | 0.2724 |
|  | rs4796568 | G | C | -0.1337 | 2.40E-06 | 0.02829 | -0.0296 | 0.0188 | 0.116 |
|  | rs59302791 | T | C | 0.3655 | 7.48E-06 | 0.08144 | -0.0352 | 0.037 | 0.3408 |
|  | rs61845257 | C | T | 0.3595 | 8.41E-06 | 0.08057 | -0.0453 | 0.0483 | 0.3479 |
|  | rs61991437 | G | C | -0.1405 | 1.01E-06 | 0.02868 | 0.0203 | 0.0196 | 0.3014 |
|  | rs6697436 | C | T | -0.1863 | 9.37E-06 | 0.04197 | 0.0126 | 0.0273 | 0.6448 |
|  | rs7251035 | C | T | 0.1293 | 7.59E-06 | 0.02882 | -0.0018 | 0.0183 | 0.9228 |
|  | rs72697615 | T | C | -0.2253 | 3.57E-06 | 0.04852 | 0.1127 | 0.0636 | 0.076421 |
|  | rs74425152 | G | A | 0.9585 | 4.32E-06 | 0.2082 | -0.1361 | 0.0678 | 0.04466 |
|  | rs75443629 | C | T | -0.2248 | 2.26E-06 | 0.04745 | -0.0052 | 0.0521 | 0.9206 |
|  | rs78042544 | G | A | 0.4243 | 5.35E-06 | 0.09306 | -0.0068 | 0.0354 | 0.8474 |
|  | rs78694250 | A | G | -0.4695 | 2.74E-06 | 0.09991 | 0.0355 | 0.0382 | 0.3516 |
|  | rs858496 | G | T | 0.1732 | 1.69E-09 | 0.02866 | 0.0082 | 0.0208 | 0.6941 |
|  | rs9894706 | G | C | -0.1323 | 6.48E-06 | 0.02929 | -0.0065 | 0.0199 | 0.744399 |
| CD3 on CD4 Treg | rs114672530 | A | G | -0.6683 | 1.46E-10 | 0.1039 | 0.0274 | 0.0711 | 0.6996 |
|  | rs142394936 | G | T | 0.6923 | 6.41E-06 | 0.1531 | -0.1965 | 0.2433 | 0.4192 |
|  | rs2595260 | C | T | -0.1558 | 5.41E-06 | 0.03419 | -0.0044 | 0.0207 | 0.833 |
|  | rs2949661 | T | C | 0.6285 | 6.97E-128 | 0.02484 | -0.0351 | 0.0207 | 0.090511 |
|  | rs35055340 | T | C | -0.4838 | 2.49E-10 | 0.07619 | 0.0796 | 0.0411 | 0.05273 |
|  | rs4396110 | A | G | -0.1253 | 4.51E-06 | 0.02728 | -0.0222 | 0.0206 | 0.2816 |
|  | rs552999 | A | C | -0.1527 | 4.83E-07 | 0.03028 | 1.00E-04 | 0.0192 | 0.9959 |
|  | rs57844740 | A | G | 0.3817 | 1.76E-06 | 0.07971 | -0.0199 | 0.044 | 0.6507 |
|  | rs6684311 | G | C | -0.2758 | 8.80E-13 | 0.03842 | 0.0304 | 0.0211 | 0.1502 |
|  | rs6697436 | C | T | -0.2029 | 1.10E-06 | 0.04155 | 0.0126 | 0.0273 | 0.6448 |
|  | rs73072986 | T | C | -0.2745 | 9.73E-06 | 0.06194 | 0.1339 | 0.1225 | 0.2744 |
| CD3 on resting Treg | rs115662161 | G | A | -0.1939 | 3.32E-06 | 0.04161 | -0.0193 | 0.0304 | 0.5263 |
|  | rs12096911 | C | T | -0.1374 | 4.90E-06 | 0.03001 | 0.0459 | 0.0192 | 0.01688 |
|  | rs1214596 | A | T | 0.6523 | 1.60E-136 | 0.02485 | -0.0326 | 0.0208 | 0.1164 |
|  | rs12752576 | G | A | -0.7279 | 4.57E-11 | 0.1101 | 0.0301 | 0.0602 | 0.6172 |
|  | rs1324073 | C | G | -0.1407 | 1.10E-06 | 0.0288 | -0.0266 | 0.0266 | 0.3183 |
|  | rs1398059 | T | G | 0.137 | 6.61E-06 | 0.03035 | -0.0039 | 0.0206 | 0.8509 |
|  | rs141784254 | G | A | -1.123 | 2.64E-07 | 0.2176 | 0.0853 | 0.068 | 0.2096 |
|  | rs146597227 | T | G | -0.2189 | 4.15E-06 | 0.04747 | -0.0028 | 0.0265 | 0.9156 |
|  | rs147902311 | T | C | 0.8593 | 7.28E-06 | 0.1912 | 0.3299 | 0.1837 | 0.07248 |
|  | rs150306980 | C | T | -0.3481 | 1.60E-06 | 0.07239 | -0.0069 | 0.0684 | 0.9197 |
|  | rs1537547 | A | G | 0.227 | 4.01E-06 | 0.04914 | -0.0447 | 0.0384 | 0.244 |
|  | rs181383275 | T | C | 0.8288 | 6.28E-06 | 0.1832 | 0.125 | 0.0785 | 0.1113 |
|  | rs187281020 | G | A | -0.5056 | 6.55E-07 | 0.1014 | -0.4014 | 0.2266 | 0.07645 |
|  | rs2015682 | T | C | -0.1482 | 2.23E-06 | 0.03125 | 0.045 | 0.0185 | 0.0151 |
|  | rs209858 | A | G | 0.3463 | 3.40E-06 | 0.07442 | 0.1014 | 0.0731 | 0.1654 |
|  | rs2122146 | C | T | -0.1597 | 3.84E-06 | 0.0345 | -0.0011 | 0.021 | 0.9601 |
|  | rs3756564 | A | G | -0.3329 | 5.08E-06 | 0.07285 | 0.0353 | 0.0478 | 0.4605 |
|  | rs4657608 | T | C | 0.1334 | 2.93E-06 | 0.02847 | -0.0023 | 0.0185 | 0.9027 |
|  | rs4707319 | G | T | -0.1526 | 8.36E-06 | 0.0342 | 0.0096 | 0.0194 | 0.619699 |
|  | rs55799444 | T | C | -0.1331 | 5.33E-06 | 0.0292 | 0.0107 | 0.0189 | 0.5708 |
|  | rs59604901 | C | T | -0.2701 | 7.59E-06 | 0.06022 | 7.00E-04 | 0.0489 | 0.9888 |
|  | rs61814886 | A | G | 0.3619 | 2.58E-16 | 0.04392 | -0.0113 | 0.0295 | 0.7007 |
|  | rs62084754 | T | C | -0.2687 | 4.46E-06 | 0.05846 | -0.051 | 0.0451 | 0.2575 |
|  | rs631487 | C | T | -0.1302 | 3.58E-06 | 0.02803 | 0.0103 | 0.0191 | 0.588901 |
|  | rs6586224 | C | T | 0.1975 | 1.15E-06 | 0.04051 | 0.0206 | 0.0344 | 0.549 |
|  | rs6658665 | G | A | 0.1538 | 2.35E-08 | 0.02746 | 0.0022 | 0.0206 | 0.9166 |
|  | rs6668517 | C | A | -0.2306 | 2.66E-08 | 0.04135 | 0.0117 | 0.0273 | 0.6687 |
|  | rs722898 | G | C | -0.1272 | 8.49E-06 | 0.02852 | 0.0356 | 0.0183 | 0.051 |
|  | rs72928038 | A | G | 0.1824 | 1.06E-07 | 0.03422 | -0.0565 | 0.0286 | 0.04845 |
|  | rs73036765 | T | C | -0.266 | 6.96E-06 | 0.05908 | 0.0365 | 0.0262 | 0.1629 |
|  | rs73072986 | T | C | -0.2906 | 3.23E-06 | 0.0623 | 0.1339 | 0.1225 | 0.2744 |
|  | rs73099996 | A | G | -0.2766 | 1.09E-06 | 0.05662 | 0.0236 | 0.0502 | 0.6391 |
|  | rs73136665 | A | C | -0.3115 | 9.51E-06 | 0.07022 | 0.0223 | 0.0665 | 0.7374 |
|  | rs74620952 | C | T | -1.355 | 4.46E-08 | 0.247 | 0.1409 | 0.0579 | 0.0149 |
|  | rs74863989 | A | C | 1.017 | 3.93E-06 | 0.2199 | -0.0598 | 0.0854 | 0.4834 |
|  | rs78042544 | G | A | 0.4514 | 1.21E-06 | 0.09279 | -0.0068 | 0.0354 | 0.8474 |
|  | rs79486472 | G | C | -0.1948 | 3.92E-06 | 0.04213 | -0.0178 | 0.0276 | 0.5196 |
|  | rs79844317 | T | C | -0.4024 | 4.23E-06 | 0.08733 | -0.321 | 0.1266 | 0.01123 |
| CD28 on CD28+ CD45RA+ CD8br | rs10447212 | T | C | 0.1449 | 0.03191 | 5.81E-06 | 0.0034 | 0.0218 | 0.8768 |
|  | rs11074594 | A | G | -0.1335 | 0.02879 | 3.70E-06 | 0.0169 | 0.0203 | 0.4034 |
|  | rs112581826 | A | G | -0.2422 | 0.04219 | 1.04E-08 | -0.059 | 0.0712 | 0.4078 |
|  | rs13147958 | A | G | -0.1874 | 0.04167 | 7.15E-06 | 0.0317 | 0.0297 | 0.2869 |
|  | rs17521485 | C | A | 0.1951 | 0.04057 | 1.59E-06 | 0.0174 | 0.0234 | 0.4567 |
|  | rs2734904 | A | G | 0.152 | 0.03068 | 7.66E-07 | -1.00E-04 | 0.0187 | 0.9978 |
|  | rs28421958 | T | C | 0.3413 | 0.07475 | 5.19E-06 | -0.031 | 0.037 | 0.4021 |
|  | rs3845728 | A | T | 2.082 | 0.4395 | 2.28E-06 | -0.161 | 0.0797 | 0.04324 |
|  | rs4747821 | C | T | -0.2408 | 0.05056 | 1.99E-06 | -0.0114 | 0.0283 | 0.686 |
|  | rs72820300 | C | T | 0.342 | 0.07046 | 1.27E-06 | 0.081 | 0.0448 | 0.07022 |
|  | rs72928038 | A | G | 0.5885 | 0.03315 | 5.13E-67 | -0.0565 | 0.0286 | 0.04845 |
|  | rs74321916 | T | A | 0.3135 | 0.06881 | 5.41E-06 | -0.0031 | 0.0312 | 0.9208 |
|  | rs75321567 | T | C | 0.879 | 0.1967 | 8.13E-06 | -0.0189 | 0.0479 | 0.6928 |
|  | rs78307830 | C | G | 0.4819 | 0.06136 | 5.64E-15 | 0.0118 | 0.0732 | 0.8725 |
|  | rs788008 | T | C | -0.1482 | 0.03094 | 1.75E-06 | 0.0238 | 0.0221 | 0.2812 |
|  | rs79921170 | A | C | 0.3378 | 0.07589 | 8.83E-06 | 0.0175 | 0.0351 | 0.6187 |
|  | rs79921170 | A | C | 0.3378 | 0.07589 | 8.83E-06 | -0.477 | 0.2906 | 0.1007 |
| CD45 on HLA DR+ T cell | rs10411493 | G | A | 0.3586 | 0.07583 | 2.35E-06 | -0.0671 | 0.0451 | 0.1366 |
|  | rs11195975 | T | C | 0.6485 | 0.1444 | 7.33E-06 | 0.069 | 0.0449 | 0.1242 |
|  | rs12884891 | A | C | 0.165 | 0.03315 | 6.79E-07 | -0.0032 | 0.0214 | 0.8808 |
|  | rs12924236 | C | G | 0.2877 | 0.03115 | 4.55E-20 | -1.00E-04 | 0.0212 | 0.9951 |
|  | rs141959698 | A | G | -1.77 | 0.3841 | 4.22E-06 | -0.1271 | 0.056 | 0.02321 |
|  | rs142492586 | C | T | -0.1762 | 0.0355 | 7.32E-07 | 0.0318 | 0.2831 | 0.9106 |
|  | rs2002814 | C | T | 0.1578 | 0.034 | 3.59E-06 | 0.0062 | 0.0214 | 0.771401 |
|  | rs200751912 | A | T | 0.5205 | 0.1102 | 2.44E-06 | -0.0416 | 0.069 | 0.5462 |
|  | rs34970051 | C | T | -0.2606 | 0.05042 | 2.50E-07 | -0.1051 | 0.0481 | 0.02879 |
|  | rs4287807 | G | A | 0.1279 | 0.02887 | 9.77E-06 | 0.0176 | 0.0188 | 0.3496 |
|  | rs45490493 | T | C | 0.6698 | 0.1491 | 7.28E-06 | 0.0498 | 0.1948 | 0.798 |
|  | rs56073410 | T | C | 0.3336 | 0.07525 | 9.60E-06 | 0.0162 | 0.1024 | 0.8745 |
|  | rs71543398 | C | T | -0.2774 | 0.06237 | 9.01E-06 | 0.1685 | 0.091 | 0.06423 |
|  | rs7296607 | A | G | 0.3008 | 0.06447 | 3.22E-06 | 0.0322 | 0.0296 | 0.2762 |
|  | rs76771115 | T | C | 0.2469 | 0.05298 | 3.30E-06 | 0.0188 | 0.0481 | 0.696299 |
|  | rs77299479 | A | G | -1.205 | 0.2573 | 2.95E-06 | 0.0206 | 0.0483 | 0.6698 |
|  | rs77647448 | A | G | 0.1994 | 0.04499 | 9.67E-06 | 0.0088 | 0.029 | 0.7625 |
|  | rs79909944 | A | G | -0.4234 | 0.09459 | 7.86E-06 | -0.0966 | 0.0383 | 0.01167 |
| CD25 on CD4 Treg | rs115155204 | T | C | 0.419 | 2.50E-06 | 0.08885 | 0.13 | 0.0738 | 0.078041 |
|  | rs115584720 | C | T | -0.6209 | 9.69E-06 | 0.1401 | -0.2351 | 0.4356 | 0.5894 |
|  | rs11598494 | C | T | -0.1145 | 6.29E-06 | 0.02531 | 0.013 | 0.0194 | 0.5036 |
|  | rs117223635 | T | C | -0.3275 | 3.26E-06 | 0.07027 | -0.0555 | 0.0939 | 0.5544 |
|  | rs12244238 | G | A | 0.1324 | 2.95E-07 | 0.02577 | -0.0033 | 0.0189 | 0.8592 |
|  | rs141518333 | T | C | 1.769 | 1.28E-06 | 0.3647 | -0.039 | 0.0518 | 0.4514 |
|  | rs141933481 | C | A | -1.52 | 9.70E-06 | 0.343 | -0.1032 | 0.0416 | 0.01317 |
|  | rs148961261 | G | A | 0.6979 | 8.45E-06 | 0.1565 | 0.0166 | 0.2669 | 0.9504 |
|  | rs1601402 | A | G | -0.1202 | 2.88E-06 | 0.02564 | -0.0033 | 0.0209 | 0.874 |
|  | rs17602422 | T | C | -0.3439 | 9.60E-06 | 0.07758 | -0.0082 | 0.0307 | 0.788501 |
|  | rs4109437 | A | G | -0.6655 | 9.90E-06 | 0.1504 | 0.0037 | 0.0512 | 0.9419 |
|  | rs4791980 | C | T | -0.1119 | 7.25E-06 | 0.0249 | 0.0035 | 0.0183 | 0.8484 |
|  | rs6939307 | T | G | -0.1245 | 2.91E-06 | 0.02657 | -0.0381 | 0.0231 | 0.099001 |
|  | rs73178594 | T | A | 0.142 | 6.83E-06 | 0.03152 | 0.0448 | 0.024 | 0.06253 |
|  | rs7324524 | A | G | -0.1813 | 8.41E-06 | 0.04064 | -0.0063 | 0.0283 | 0.8236 |
|  | rs75991976 | T | C | -0.2459 | 2.22E-06 | 0.05188 | 0.0263 | 0.0268 | 0.3262 |
|  | rs7601164 | C | T | 0.1204 | 1.52E-06 | 0.02499 | 0.04 | 0.0188 | 0.03335 |
|  | rs80185999 | T | C | -0.5247 | 2.01E-06 | 0.1102 | -0.1221 | 0.0753 | 0.1046 |
|  | rs80267112 | A | C | -3.291 | 5.69E-06 | 0.7242 | -0.0997 | 0.0641 | 0.1199 |
|  | rs9422867 | A | G | 0.1767 | 2.63E-06 | 0.03755 | 0.0239 | 0.022 | 0.276 |
|  | rs9596433 | T | C | -0.153 | 9.08E-06 | 0.03442 | 0.0022 | 0.0279 | 0.9371 |
| CD16 on CD14- CD16+ monocyte | rs112655418 | C | A | -0.166 | 6.91E-06 | 0.03687 | 0.0031 | 0.0201 | 0.8794 |
|  | rs11860582 | A | T | -0.1431 | 9.25E-06 | 0.03223 | 0.0146 | 0.0275 | 0.595799 |
|  | rs12239946 | C | T | 0.2423 | 1.10E-07 | 0.04555 | -0.0296 | 0.0229 | 0.1965 |
|  | rs12737621 | G | T | -0.1212 | 1.24E-06 | 0.02496 | 0.0087 | 0.0187 | 0.6405 |
|  | rs139085673 | G | A | -1.468 | 5.92E-06 | 0.3236 | 0.0325 | 0.0681 | 0.632799 |
|  | rs139731096 | C | G | 0.122 | 1.25E-06 | 0.02514 | -0.0061 | 0.0221 | 0.7824 |
|  | rs140966292 | C | A | -0.5466 | 1.37E-07 | 0.1035 | 0.0391 | 0.0608 | 0.5203 |
|  | rs17400517 | T | G | -1.146 | 1.00E-200 | 0.02322 | -0.0035 | 0.0299 | 0.9069 |
|  | rs17817999 | C | T | 0.1414 | 8.25E-06 | 0.03168 | 0.0351 | 0.0248 | 0.158 |
|  | rs2184197 | G | C | -0.1114 | 9.71E-06 | 0.02514 | -0.019 | 0.0184 | 0.3013 |
|  | rs2502806 | G | A | 0.209 | 5.19E-16 | 0.02566 | 0.0217 | 0.0185 | 0.241 |
|  | rs2638546 | A | G | 0.2146 | 7.42E-06 | 0.04782 | -0.0143 | 0.0461 | 0.756599 |
|  | rs35804175 | A | T | 0.2027 | 9.81E-06 | 0.04578 | 0.0241 | 0.0281 | 0.3914 |
|  | rs3856266 | G | T | -0.2278 | 9.59E-11 | 0.03508 | -0.0388 | 0.0202 | 0.05475 |
|  | rs4644525 | C | A | -0.2397 | 5.30E-06 | 0.05257 | -0.0543 | 0.0509 | 0.2863 |
|  | rs61806480 | A | G | -0.5168 | 6.83E-06 | 0.1147 | -0.0621 | 0.1234 | 0.615 |
|  | rs6671271 | A | G | -0.5501 | 2.70E-13 | 0.07499 | -0.0721 | 0.0333 | 0.03049 |
|  | rs72700199 | T | C | -0.2586 | 6.87E-13 | 0.03587 | -0.0251 | 0.0205 | 0.2213 |
|  | rs73152567 | C | T | 2.332 | 5.73E-06 | 0.5132 | 0.2634 | 0.0998 | 0.008298 |
|  | rs74341264 | A | G | 0.5172 | 1.96E-42 | 0.0374 | 0.0247 | 0.0314 | 0.4307 |
|  | rs78639067 | G | A | -0.5895 | 4.26E-06 | 0.128 | 0.0036 | 0.0709 | 0.9595 |
|  | rs78804199 | T | C | 0.4148 | 2.26E-07 | 0.07996 | 0.1412 | 0.1533 | 0.3568 |
|  | rs79433597 | A | G | 0.3949 | 6.35E-06 | 0.08736 | 0.0359 | 0.0638 | 0.5733 |
| CD40 on CD14+ CD16+ monocyte | rs113063605 | G | A | 0.1912 | 1.23E-06 | 0.03935 | 8.00E-04 | 0.0266 | 0.9757 |
|  | rs117572234 | C | T | 0.4177 | 8.06E-06 | 0.09345 | 0.2827 | 0.5047 | 0.5754 |
|  | rs11906777 | A | G | 0.2037 | 2.89E-07 | 0.03963 | 0.0446 | 0.0257 | 0.08202 |
|  | rs11992049 | T | C | 0.1873 | 5.83E-06 | 0.04126 | -0.0213 | 0.0362 | 0.5564 |
|  | rs12443555 | A | G | 1.31 | 5.79E-07 | 0.2617 | 0.0014 | 0.0433 | 0.9739 |
|  | rs12517933 | C | T | -0.1387 | 4.72E-06 | 0.03026 | -0.0891 | 0.0395 | 0.02399 |
|  | rs138540869 | T | C | 0.3866 | 1.18E-06 | 0.07943 | 0.1255 | 0.1558 | 0.4204 |
|  | rs139839882 | T | C | 0.3046 | 7.89E-07 | 0.06158 | -0.0274 | 0.0785 | 0.727301 |
|  | rs141130258 | T | G | 0.4455 | 4.58E-06 | 0.09706 | 0.1069 | 0.1521 | 0.4821 |
|  | rs148922794 | T | C | 0.2587 | 8.55E-08 | 0.0482 | -0.0673 | 0.0652 | 0.302 |
|  | rs149632680 | G | A | 0.8538 | 4.76E-06 | 0.1863 | 0.0204 | 0.1526 | 0.8936 |
|  | rs1801274 | G | A | 0.4646 | 2.06E-70 | 0.02562 | 0.0353 | 0.0182 | 0.0522 |
|  | rs2664523 | A | G | 0.3595 | 1.03E-08 | 0.06265 | -0.0186 | 0.0292 | 0.5255 |
|  | rs4810481 | G | T | -0.4198 | 7.18E-06 | 0.0934 | 0.0383 | 0.0335 | 0.2531 |
|  | rs60072425 | C | A | 1.117 | 1.34E-06 | 0.2307 | 0.0812 | 0.0411 | 0.04822 |
|  | rs6687275 | G | T | 0.1985 | 3.17E-12 | 0.02838 | -0.0375 | 0.0254 | 0.1401 |
|  | rs72749639 | A | C | 1.566 | 9.71E-07 | 0.3192 | 0.0549 | 0.0411 | 0.182 |
|  | rs72927495 | C | T | 0.3454 | 7.47E-06 | 0.077 | -0.0562 | 0.0647 | 0.3844 |
|  | rs745307 | A | G | 0.6978 | 3.78E-92 | 0.03329 | 0.0127 | 0.0209 | 0.5425 |
|  | rs77649171 | T | C | 0.5129 | 4.00E-06 | 0.1111 | -0.0738 | 0.203 | 0.716199 |
| HLA DR on CD14+ CD16- monocyte | rs112651313 | C | T | 0.1781 | 0.03977 | 7.72E-06 | -0.0124 | 0.0304 | 0.6834 |
|  | rs113337372 | A | G | -0.3065 | 0.06892 | 8.97E-06 | -0.0508 | 0.069 | 0.462 |
|  | rs113555953 | A | C | 0.5125 | 0.1129 | 5.86E-06 | 0.0201 | 0.0636 | 0.7523 |
|  | rs11788133 | A | G | -0.1567 | 0.03359 | 3.20E-06 | -0.0339 | 0.0291 | 0.2438 |
|  | rs13168479 | T | C | -0.5935 | 0.1334 | 8.85E-06 | -0.0026 | 0.112 | 0.9816 |
|  | rs143858253 | C | T | 0.1754 | 0.039 | 7.12E-06 | -0.1065 | 0.0589 | 0.070551 |
|  | rs146479897 | G | C | 0.4693 | 0.1013 | 3.72E-06 | 0.0428 | 0.0877 | 0.625801 |
|  | rs146897285 | A | C | 0.9798 | 0.2073 | 2.36E-06 | -0.0044 | 0.0761 | 0.9543 |
|  | rs148974843 | G | C | 0.2723 | 0.05871 | 3.64E-06 | -0.026 | 0.0389 | 0.5038 |
|  | rs150542843 | T | C | -0.3123 | 0.07004 | 8.51E-06 | -0.0265 | 0.2388 | 0.9118 |
|  | rs150649461 | C | G | -0.2944 | 0.06482 | 5.77E-06 | -0.0035 | 0.0565 | 0.9505 |
|  | rs1800973 | A | C | -0.3123 | 0.04579 | 1.06E-11 | -0.0485 | 0.0415 | 0.2426 |
|  | rs188394267 | T | C | -0.2065 | 0.04611 | 7.72E-06 | 0.0606 | 0.0669 | 0.3649 |
|  | rs2394447 | T | C | 0.1741 | 0.03333 | 1.85E-07 | -0.0126 | 0.0307 | 0.6818 |
|  | rs35848415 | G | A | -0.274 | 0.05943 | 4.14E-06 | 0.0244 | 0.0318 | 0.4424 |
|  | rs62005962 | A | G | -0.1224 | 0.02703 | 6.10E-06 | -0.0082 | 0.0184 | 0.6546 |
|  | rs80032720 | A | G | 0.4253 | 0.0545 | 7.76E-15 | 0.2037 | 0.1629 | 0.211 |
|  | rs8043889 | T | C | -0.1291 | 0.0272 | 2.16E-06 | 0.0227 | 0.0199 | 0.255 |
|  | rs9261756 | G | A | -0.1749 | 0.03415 | 3.22E-07 | 0.0689 | 0.0309 | 0.02599 |
|  | rs9270585 | T | C | -0.6054 | 0.0279 | 3.04E-98 | 0.04 | 0.0206 | 0.05211 |
|  | rs9271768 | G | A | -0.5252 | 0.02936 | 1.21E-68 | -0.0526 | 0.0216 | 0.01483 |
|  | rs9271768 | G | A | -0.5252 | 0.02936 | 1.21E-68 | 0.0112 | 0.0272 | 0.680801 |
|  | rs9469245 | T | C | -0.2672 | 0.02801 | 2.52E-21 | 0.0373 | 0.0208 | 0.07365 |
| CD40 on CD14- CD16+ monocyte | rs10762188 | A | G | 0.1247 | 9.71E-06 | 0.02815 | -0.0199 | 0.0185 | 0.2819 |
|  | rs11032433 | C | T | -0.1433 | 3.15E-06 | 0.0307 | 0.0251 | 0.0224 | 0.2638 |
|  | rs111436326 | T | C | 0.4119 | 2.63E-09 | 0.06902 | 0.0404 | 0.1012 | 0.6896 |
|  | rs12122366 | T | C | 0.2105 | 3.00E-07 | 0.04101 | -0.0271 | 0.0416 | 0.514301 |
|  | rs12233381 | A | G | 0.1878 | 6.16E-06 | 0.04148 | 0.0062 | 0.0271 | 0.8194 |
|  | rs139376787 | A | G | -0.3125 | 6.85E-11 | 0.04775 | -0.176 | 0.1106 | 0.1116 |
|  | rs139922750 | A | C | 2.548 | 5.24E-06 | 0.5587 | 0.1007 | 0.0615 | 0.1019 |
|  | rs140839053 | T | C | 0.3081 | 5.17E-06 | 0.0675 | 0.028 | 0.0496 | 0.572 |
|  | rs143602490 | T | A | 0.6066 | 2.85E-06 | 0.1294 | 0.0019 | 0.0543 | 0.9722 |
|  | rs146963227 | A | G | -0.5007 | 8.23E-07 | 0.1014 | 0.2025 | 0.2885 | 0.4829 |
|  | rs148765873 | C | T | -0.3497 | 2.69E-08 | 0.06275 | 0.1402 | 0.1047 | 0.1805 |
|  | rs1801274 | G | A | 0.5292 | 1.29E-90 | 0.02548 | 0.0353 | 0.0182 | 0.0522 |
|  | rs34658867 | C | A | 0.4042 | 3.04E-11 | 0.06064 | -0.1024 | 0.0629 | 0.1032 |
|  | rs35989582 | A | G | 0.1363 | 7.96E-06 | 0.03048 | 0.0055 | 0.0193 | 0.778001 |
|  | rs3788395 | A | G | 0.1299 | 6.38E-06 | 0.02874 | -0.005 | 0.0229 | 0.8287 |
|  | rs4766578 | A | T | -0.1365 | 2.19E-07 | 0.0263 | 0.0164 | 0.0184 | 0.3711 |
|  | rs55872505 | G | T | 0.5977 | 9.88E-06 | 0.1351 | 0.082 | 0.0543 | 0.1305 |
|  | rs56330830 | C | T | 0.1752 | 1.31E-06 | 0.03614 | -0.0246 | 0.0212 | 0.2458 |
|  | rs60072425 | C | A | 1.1 | 1.57E-06 | 0.2288 | 0.0812 | 0.0411 | 0.04822 |
|  | rs6032211 | A | G | 0.189 | 5.95E-09 | 0.0324 | 0.0064 | 0.0272 | 0.8138 |
|  | rs6073077 | A | G | 0.2468 | 6.04E-06 | 0.05447 | 0.1585 | 0.1296 | 0.2216 |
|  | rs61784602 | C | T | 1.404 | 7.92E-06 | 0.3137 | -0.0871 | 0.1256 | 0.488 |
|  | rs6687275 | G | T | 0.339 | 7.56E-33 | 0.02812 | -0.0375 | 0.0254 | 0.1401 |
|  | rs745307 | A | G | 0.7089 | 5.03E-94 | 0.03346 | 0.0127 | 0.0209 | 0.5425 |
|  | rs75698615 | A | T | -0.2587 | 3.34E-06 | 0.05556 | -0.021 | 0.0565 | 0.710699 |
|  | rs77649171 | T | C | 0.5253 | 2.11E-06 | 0.1106 | -0.0738 | 0.203 | 0.716199 |
|  | rs77774351 | G | C | 0.374 | 2.29E-08 | 0.06677 | -0.017 | 0.0366 | 0.6426 |
|  | rs78162364 | T | G | 0.4438 | 7.11E-06 | 0.09869 | 0.0471 | 0.0601 | 0.433 |
|  | rs78824072 | T | G | 0.1931 | 5.89E-06 | 0.04257 | 0.0601 | 0.0566 | 0.2887 |
| HLA DR on CD14+ monocyte | rs112651313 | C | T | 0.1779 | 8.22E-06 | 0.03984 | -0.0124 | 0.0304 | 0.6834 |
|  | rs113337372 | A | G | -0.3163 | 4.80E-06 | 0.06905 | -0.0508 | 0.069 | 0.462 |
|  | rs113555953 | A | C | 0.5169 | 5.09E-06 | 0.1132 | 0.0201 | 0.0636 | 0.7523 |
|  | rs11788133 | A | G | -0.1591 | 2.37E-06 | 0.03366 | -0.0339 | 0.0291 | 0.2438 |
|  | rs13168479 | T | C | -0.5921 | 9.67E-06 | 0.1336 | -0.0026 | 0.112 | 0.9816 |
|  | rs143858253 | C | T | 0.1762 | 6.69E-06 | 0.03908 | -0.1065 | 0.0589 | 0.070551 |
|  | rs146479897 | G | C | 0.4649 | 4.79E-06 | 0.1015 | 0.0428 | 0.0877 | 0.625801 |
|  | rs146897285 | A | C | 0.9514 | 4.85E-06 | 0.2078 | -0.0044 | 0.0761 | 0.9543 |
|  | rs147628007 | G | A | 0.2853 | 8.04E-06 | 0.06382 | 0.0363 | 0.0545 | 0.5052 |
|  | rs148974843 | G | C | 0.2717 | 3.97E-06 | 0.05882 | -0.026 | 0.0389 | 0.5038 |
|  | rs1800973 | A | C | -0.315 | 7.74E-12 | 0.04587 | -0.0485 | 0.0415 | 0.2426 |
|  | rs188394267 | T | C | -0.2077 | 7.13E-06 | 0.04619 | 0.0606 | 0.0669 | 0.3649 |
|  | rs2394447 | T | C | 0.1669 | 5.91E-07 | 0.03337 | -0.0126 | 0.0307 | 0.6818 |
|  | rs35848415 | G | A | -0.2693 | 6.31E-06 | 0.05955 | 0.0244 | 0.0318 | 0.4424 |
|  | rs62005962 | A | G | -0.1213 | 7.66E-06 | 0.02708 | -0.0082 | 0.0184 | 0.6546 |
|  | rs80032720 | A | G | 0.4142 | 3.97E-14 | 0.05456 | 0.2037 | 0.1629 | 0.211 |
|  | rs8043889 | T | C | -0.1283 | 2.59E-06 | 0.02725 | 0.0227 | 0.0199 | 0.255 |
|  | rs9261756 | G | A | -0.1687 | 8.29E-07 | 0.03418 | 0.0689 | 0.0309 | 0.02599 |
|  | rs9270585 | T | C | -0.5871 | 5.56E-92 | 0.02804 | 0.04 | 0.0206 | 0.05211 |
|  | rs9271768 | G | A | -0.5084 | 3.56E-64 | 0.02947 | -0.0526 | 0.0216 | 0.01483 |
|  | rs9271768 | G | A | -0.5084 | 3.56E-64 | 0.02947 | 0.0112 | 0.0272 | 0.680801 |
|  | rs9469245 | T | C | -0.2607 | 2.56E-20 | 0.02805 | 0.0373 | 0.0208 | 0.07365 |
| CX3CR1 on CD14- CD16- | rs115232009 | G | T | 1.535 | 0.325 | 2.43E-06 | -0.0149 | 0.0372 | 0.6891 |
|  | rs117377596 | C | T | 0.2749 | 0.06158 | 8.26E-06 | -0.3707 | 0.2868 | 0.1963 |
|  | rs117379843 | G | C | 0.3719 | 0.07579 | 9.70E-07 | -0.0154 | 0.0406 | 0.704601 |
|  | rs12614991 | A | C | 2.311 | 0.5204 | 9.25E-06 | -0.214 | 0.0829 | 0.009828 |
|  | rs139062294 | C | A | 1.618 | 0.3585 | 6.57E-06 | -0.2362 | 0.1079 | 0.02854 |
|  | rs144126567 | G | C | 0.5108 | 0.03154 | 5.51E-57 | -0.0371 | 0.0395 | 0.3476 |
|  | rs146027392 | C | T | -0.4479 | 0.09878 | 5.97E-06 | 0.0396 | 0.063 | 0.5294 |
|  | rs147571616 | A | T | -1.407 | 0.3002 | 2.87E-06 | 0.0194 | 0.0363 | 0.5939 |
|  | rs17672079 | A | T | 0.2135 | 0.0468 | 5.22E-06 | 0.0217 | 0.0265 | 0.4133 |
|  | rs34331363 | T | C | 0.2708 | 0.05796 | 3.07E-06 | -0.0808 | 0.0256 | 0.001623 |
|  | rs35579164 | C | G | 0.3663 | 0.07954 | 4.27E-06 | -0.0058 | 0.0517 | 0.9103 |
|  | rs3826742 | T | C | -0.1098 | 0.02446 | 7.35E-06 | -0.0152 | 0.021 | 0.4681 |
|  | rs4648363 | T | C | -0.1302 | 0.0257 | 4.30E-07 | 0.0059 | 0.0184 | 0.747799 |
|  | rs61847551 | G | A | 0.1931 | 0.04202 | 4.48E-06 | 0.027 | 0.0321 | 0.4 |
|  | rs62435155 | A | C | 2.072 | 0.456 | 5.69E-06 | -0.0269 | 0.0674 | 0.6896 |
|  | rs6661135 | T | C | 0.6151 | 0.1356 | 5.90E-06 | -0.0147 | 0.0402 | 0.715501 |
|  | rs6813694 | C | A | -0.844 | 0.1784 | 2.32E-06 | 0.0136 | 0.058 | 0.814 |
|  | rs71521373 | G | C | -0.1591 | 0.03588 | 9.46E-06 | 1.00E-04 | 0.0228 | 0.9956 |
|  | rs876036 | C | T | 0.1655 | 0.02824 | 5.07E-09 | 0.028 | 0.0198 | 0.1571 |
|  | rs9823718 | G | C | -0.2149 | 0.03622 | 3.24E-09 | -0.01 | 0.036 | 0.780601 |
| CX3CR1 on CD14+ CD16+ monocyte | rs10011142 | T | C | -0.1374 | 9.37E-06 | 0.03097 | 0.0576 | 0.0319 | 0.07113 |
|  | rs10499824 | T | C | 0.439 | 6.23E-06 | 0.09702 | -0.2451 | 0.3384 | 0.469 |
|  | rs117043578 | T | C | 2.527 | 9.91E-06 | 0.5709 | -0.0785 | 0.0915 | 0.3908 |
|  | rs11768517 | T | G | 0.1326 | 8.75E-07 | 0.02691 | -0.002 | 0.0189 | 0.9142 |
|  | rs117871360 | G | A | -0.7288 | 8.47E-06 | 0.1634 | 0.0718 | 0.0349 | 0.03959 |
|  | rs117896307 | C | T | -0.257 | 8.36E-06 | 0.0576 | 0.1447 | 0.065 | 0.02591 |
|  | rs117940302 | A | G | 0.4039 | 2.28E-06 | 0.08531 | -0.047 | 0.0761 | 0.536701 |
|  | rs12372861 | G | C | -1.136 | 6.27E-06 | 0.2511 | -0.0877 | 0.1321 | 0.5066 |
|  | rs1376624 | C | T | 0.1412 | 3.90E-06 | 0.03053 | -0.0464 | 0.0241 | 0.05412 |
|  | rs143002526 | A | T | -0.55 | 1.10E-06 | 0.1127 | -0.0479 | 0.0704 | 0.4963 |
|  | rs148057702 | T | C | -0.3442 | 4.01E-06 | 0.07454 | -0.0942 | 0.0656 | 0.151 |
|  | rs149100 | G | T | -0.1347 | 8.49E-08 | 0.0251 | 0.0024 | 0.0193 | 0.9009 |
|  | rs16843658 | T | C | -0.3609 | 1.01E-06 | 0.07369 | 0.0614 | 0.0675 | 0.3634 |
|  | rs16867973 | G | A | 0.1506 | 8.30E-06 | 0.03374 | 0.0025 | 0.0282 | 0.9301 |
|  | rs17095454 | C | T | -1.15 | 3.15E-07 | 0.2244 | 0.2846 | 0.1642 | 0.08309 |
|  | rs17549676 | A | G | -0.1916 | 7.84E-06 | 0.04281 | 0.0383 | 0.046 | 0.4049 |
|  | rs1801274 | G | A | -0.4628 | 2.62E-69 | 0.02573 | 0.0353 | 0.0182 | 0.0522 |
|  | rs182244806 | T | C | -0.7264 | 6.93E-06 | 0.1613 | -0.0868 | 0.0985 | 0.3782 |
|  | rs2649752 | A | C | 0.1581 | 3.51E-08 | 0.02862 | -0.0096 | 0.0199 | 0.630001 |
|  | rs28491136 | T | C | -0.1603 | 7.18E-06 | 0.03567 | 0.0281 | 0.0216 | 0.1926 |
|  | rs372655475 | T | G | -0.2806 | 9.19E-06 | 0.06317 | -0.0154 | 0.0357 | 0.6656 |
|  | rs4957969 | G | A | -0.2538 | 2.10E-06 | 0.05341 | -0.0466 | 0.0363 | 0.1994 |
|  | rs58215329 | T | C | 0.1816 | 3.44E-06 | 0.03906 | 0.0215 | 0.0356 | 0.5445 |
|  | rs59871438 | C | T | -0.962 | 6.14E-06 | 0.2124 | 0.1433 | 0.2552 | 0.574399 |
|  | rs62170788 | T | C | -0.2816 | 4.93E-06 | 0.06155 | 0.002 | 0.0356 | 0.955 |
|  | rs6952061 | T | C | 0.2141 | 8.18E-06 | 0.04792 | 0.0437 | 0.0327 | 0.1825 |
|  | rs72918158 | A | G | -1.499 | 1.33E-06 | 0.3095 | 0.0839 | 0.0543 | 0.1223 |
|  | rs74574092 | C | T | 0.2025 | 2.68E-06 | 0.04307 | -0.0588 | 0.0396 | 0.1377 |
|  | rs7791415 | T | C | -0.4614 | 1.69E-06 | 0.0962 | -0.0236 | 0.0458 | 0.6052 |
|  | rs894107 | C | T | 0.1991 | 3.41E-07 | 0.03898 | 0.0159 | 0.0237 | 0.5011 |
|  | rs9542786 | C | A | -0.2675 | 2.94E-06 | 0.05713 | -0.0053 | 0.0379 | 0.8888 |
|  | rs9823718 | G | C | -0.3271 | 5.15E-19 | 0.03651 | -0.01 | 0.036 | 0.780601 |
| CCR2 on CD14+ CD16- monocyte | rs10081735 | A | G | 0.2007 | 0.04193 | 1.76E-06 | 0.0344 | 0.0228 | 0.1316 |
|  | rs10878258 | G | A | -0.1348 | 0.02549 | 1.32E-07 | 0.0469 | 0.0188 | 0.01272 |
|  | rs112976913 | C | T | 2.633 | 0.5828 | 6.43E-06 | -0.0294 | 0.0547 | 0.590999 |
|  | rs114744368 | T | C | -0.6352 | 0.1408 | 6.60E-06 | 0.0202 | 0.034 | 0.552901 |
|  | rs116078872 | T | G | -0.8485 | 0.1914 | 9.52E-06 | 0.0271 | 0.0379 | 0.475 |
|  | rs12185230 | A | G | 2.962 | 0.6555 | 6.41E-06 | -0.1449 | 0.0664 | 0.02912 |
|  | rs12613485 | A | G | -0.1948 | 0.04389 | 9.32E-06 | -0.0215 | 0.036 | 0.551099 |
|  | rs13060505 | G | C | 0.4217 | 0.08148 | 2.39E-07 | -0.027 | 0.0753 | 0.7206 |
|  | rs1386098 | A | G | -1.239 | 0.2791 | 9.22E-06 | -0.2201 | 0.231 | 0.3406 |
|  | rs141411805 | G | C | -0.5993 | 0.1027 | 5.80E-09 | 0.0105 | 0.0529 | 0.8432 |
|  | rs147404212 | A | G | 1.964 | 0.4343 | 6.30E-06 | -0.0234 | 0.0386 | 0.5454 |
|  | rs190371975 | G | C | 0.4458 | 0.1002 | 8.95E-06 | -0.2843 | 0.165 | 0.084971 |
|  | rs2160337 | G | T | 0.1301 | 0.02904 | 7.62E-06 | -0.0101 | 0.0213 | 0.6344 |
|  | rs2270454 | T | C | -0.2457 | 0.05383 | 5.19E-06 | -0.0218 | 0.027 | 0.4196 |
|  | rs2426939 | C | A | -0.1098 | 0.02439 | 6.97E-06 | -0.0147 | 0.0192 | 0.443 |
|  | rs2683681 | A | G | 0.1117 | 0.02488 | 7.35E-06 | -0.0045 | 0.0183 | 0.8075 |
|  | rs28561969 | T | C | -0.1136 | 0.02484 | 4.90E-06 | 0.0036 | 0.019 | 0.8507 |
|  | rs4683184 | A | G | 0.2454 | 0.02618 | 1.17E-20 | -0.0334 | 0.0188 | 0.075669 |
|  | rs56407854 | A | G | 0.1508 | 0.03271 | 4.15E-06 | -0.0073 | 0.0229 | 0.749701 |
|  | rs62327180 | A | G | -0.1199 | 0.02683 | 8.11E-06 | 0.0452 | 0.0192 | 0.01836 |
|  | rs7000391 | C | T | 0.16 | 0.0348 | 4.43E-06 | -0.0258 | 0.0316 | 0.4138 |
|  | rs777404 | A | G | 2.407 | 0.495 | 1.20E-06 | -0.0304 | 0.0432 | 0.4813 |
|  | rs78109162 | A | G | -0.2786 | 0.06076 | 4.69E-06 | 0.0195 | 0.0411 | 0.635501 |
|  | rs7896883 | C | T | 0.1675 | 0.0374 | 7.78E-06 | 0.0529 | 0.0222 | 0.01698 |
|  | rs79049673 | T | C | 0.351 | 0.07529 | 3.25E-06 | -0.0045 | 0.0668 | 0.9459 |
|  | rs79825014 | T | G | -0.4165 | 0.06803 | 1.02E-09 | 0.0163 | 0.0507 | 0.7474 |
| CCR2 on monocyte | rs10878258 | G | A | -0.1382 | 8.13E-08 | 0.02572 | 0.0469 | 0.0188 | 0.01272 |
|  | rs113111100 | A | G | -0.4412 | 2.82E-06 | 0.09405 | 0.1754 | 0.2295 | 0.4447 |
|  | rs11606662 | G | T | -0.1437 | 9.52E-06 | 0.03241 | -0.0134 | 0.0258 | 0.6045 |
|  | rs116966304 | A | G | -0.4221 | 5.91E-06 | 0.09304 | -0.0401 | 0.1136 | 0.723801 |
|  | rs12185230 | A | G | 3.053 | 4.01E-06 | 0.661 | -0.1449 | 0.0664 | 0.02912 |
|  | rs12613485 | A | G | -0.2065 | 3.36E-06 | 0.04436 | -0.0215 | 0.036 | 0.551099 |
|  | rs13060505 | G | C | 0.382 | 3.76E-06 | 0.08248 | -0.027 | 0.0753 | 0.7206 |
|  | rs141411805 | G | C | -0.5601 | 7.20E-08 | 0.1038 | 0.0105 | 0.0529 | 0.8432 |
|  | rs147120184 | T | A | 0.2975 | 5.46E-06 | 0.06535 | -0.054 | 0.0651 | 0.4063 |
|  | rs149527001 | A | G | 1.522 | 8.47E-06 | 0.3414 | -0.1108 | 0.1067 | 0.2988 |
|  | rs150805883 | C | T | -0.5869 | 8.34E-06 | 0.1315 | 0.1754 | 0.0978 | 0.072699 |
|  | rs17017001 | A | C | -0.1427 | 8.03E-06 | 0.03193 | 0.0473 | 0.024 | 0.04908 |
|  | rs187328631 | G | A | 0.3961 | 3.21E-06 | 0.08492 | -0.0058 | 0.0508 | 0.9088 |
|  | rs192191749 | T | C | 1.019 | 6.96E-06 | 0.2265 | -0.2227 | 0.1793 | 0.2143 |
|  | rs35526800 | T | A | 0.4494 | 7.30E-06 | 0.1001 | -0.0613 | 0.0313 | 0.05005 |
|  | rs4683184 | A | G | 0.2224 | 6.59E-17 | 0.0265 | -0.0334 | 0.0188 | 0.075669 |
|  | rs625761 | A | G | 0.1266 | 4.89E-06 | 0.02766 | -0.0108 | 0.0218 | 0.621999 |
|  | rs74124962 | G | T | -0.159 | 3.27E-07 | 0.03108 | 0.0505 | 0.0344 | 0.1419 |
|  | rs777415 | C | T | 2.348 | 3.02E-06 | 0.502 | -0.0246 | 0.0431 | 0.5681 |
|  | rs77772425 | T | G | -0.2415 | 4.05E-06 | 0.05232 | 0.0725 | 0.0546 | 0.1843 |
|  | rs78246754 | T | C | 0.3233 | 6.04E-06 | 0.07135 | -0.0034 | 0.0362 | 0.9242 |
|  | rs79049673 | T | C | 0.3447 | 6.26E-06 | 0.07619 | -0.0045 | 0.0668 | 0.9459 |
|  | rs79641389 | C | A | -0.4085 | 3.13E-09 | 0.06877 | 0.0159 | 0.0507 | 0.753999 |
| SSC-A on CD14+ monocyte | rs10202811 | A | G | -0.2937 | 8.29E-06 | 0.06578 | -0.0178 | 0.0659 | 0.787 |
|  | rs10488833 | G | C | -0.2117 | 1.39E-06 | 0.04376 | 0.0461 | 0.0474 | 0.3314 |
|  | rs10774278 | T | C | -0.1283 | 2.22E-06 | 0.02705 | -0.0124 | 0.0211 | 0.5581 |
|  | rs10784763 | G | C | -0.1296 | 1.53E-06 | 0.02691 | -0.002 | 0.0182 | 0.9136 |
|  | rs11228503 | T | C | -0.1555 | 9.84E-07 | 0.03171 | -0.0631 | 0.0205 | 0.002042 |
|  | rs1152928 | G | A | -0.7519 | 2.48E-13 | 0.1023 | -0.0946 | 0.0629 | 0.1326 |
|  | rs11554159 | A | G | -0.2988 | 1.82E-25 | 0.0284 | 0.0271 | 0.021 | 0.1965 |
|  | rs12276856 | T | C | 0.1441 | 2.09E-06 | 0.03031 | 0.0221 | 0.0279 | 0.4276 |
|  | rs12370645 | G | A | -0.6901 | 4.82E-06 | 0.1507 | -0.1027 | 0.0688 | 0.1354 |
|  | rs12478601 | T | C | -0.1442 | 2.90E-07 | 0.02805 | -0.0095 | 0.0182 | 0.6026 |
|  | rs13231864 | C | T | 0.1286 | 7.60E-06 | 0.02868 | 0.0069 | 0.0184 | 0.707301 |
|  | rs148146302 | C | G | -0.8012 | 1.65E-20 | 0.08571 | 0.0498 | 0.1432 | 0.7281 |
|  | rs1800973 | A | C | -1.048 | 3.30E-114 | 0.04423 | -0.0485 | 0.0415 | 0.2426 |
|  | rs35637068 | C | G | 0.1393 | 7.89E-07 | 0.02815 | -0.001 | 0.0184 | 0.9563 |
|  | rs445 | T | C | 0.229 | 4.94E-12 | 0.03302 | -0.0127 | 0.0392 | 0.746 |
|  | rs4947752 | C | T | 0.1395 | 4.63E-07 | 0.02761 | -0.0221 | 0.0194 | 0.2541 |
|  | rs57059400 | T | A | 0.1834 | 8.15E-06 | 0.04104 | -0.068 | 0.0349 | 0.05164 |
|  | rs62026668 | T | C | -0.1542 | 8.97E-06 | 0.03467 | -0.0701 | 0.0204 | 0.0006 |
|  | rs62070801 | C | T | 0.1178 | 8.83E-06 | 0.02646 | 0.0069 | 0.0185 | 0.7092 |
|  | rs72736734 | T | C | 0.3008 | 3.44E-06 | 0.06466 | 0.0258 | 0.0509 | 0.611999 |
|  | rs7309974 | A | G | 0.191 | 2.80E-06 | 0.04069 | 0.007 | 0.036 | 0.8461 |
|  | rs73175542 | A | G | 0.2957 | 2.36E-07 | 0.05709 | 0.0033 | 0.0469 | 0.9442 |
|  | rs75994780 | A | G | -0.3607 | 4.48E-11 | 0.05457 | -0.0105 | 0.0756 | 0.8895 |
|  | rs80216229 | C | T | -0.4355 | 1.94E-06 | 0.09132 | -0.1083 | 0.0546 | 0.04743 |
|  | rs9616832 | C | T | -0.2208 | 1.73E-06 | 0.04608 | -0.0278 | 0.0357 | 0.4374 |
| SSC-A on NK | rs1006368 | T | C | 0.4144 | 0.04052 | 3.61E-24 | 0.0241 | 0.0331 | 0.4659 |
|  | rs10512469 | T | C | -0.1684 | 0.03658 | 4.31E-06 | -0.048 | 0.0211 | 0.02286 |
|  | rs11081793 | G | A | -0.3945 | 0.08374 | 2.57E-06 | -0.1166 | 0.0932 | 0.2111 |
|  | rs11703193 | A | G | -0.1793 | 0.03727 | 1.57E-06 | 0.0275 | 0.0248 | 0.2684 |
|  | rs12594783 | C | T | -0.1395 | 0.03061 | 5.42E-06 | -0.0019 | 0.0202 | 0.9262 |
|  | rs147596499 | G | A | 0.4951 | 0.1098 | 6.78E-06 | -0.0152 | 0.1485 | 0.9186 |
|  | rs17606146 | A | G | -0.2168 | 0.04794 | 6.37E-06 | -0.0235 | 0.039 | 0.5461 |
|  | rs188107682 | A | G | -0.4965 | 0.1093 | 5.74E-06 | 0.2755 | 0.2709 | 0.3092 |
|  | rs2198787 | C | A | -0.2652 | 0.05319 | 6.55E-07 | 0.1082 | 0.0683 | 0.1133 |
|  | rs28691703 | C | T | 0.1706 | 0.03639 | 2.87E-06 | 0.0119 | 0.0206 | 0.5644 |
|  | rs2869804 | G | A | -0.1514 | 0.03364 | 7.08E-06 | -0.0218 | 0.0223 | 0.3276 |
|  | rs2915158 | G | A | 0.7087 | 0.1574 | 6.93E-06 | 0.0552 | 0.0432 | 0.2017 |
|  | rs35150190 | T | G | -0.1472 | 0.03246 | 6.00E-06 | 0.0251 | 0.0208 | 0.2259 |
|  | rs6850820 | A | G | -0.1642 | 0.03573 | 4.48E-06 | -0.0459 | 0.0198 | 0.02025 |
|  | rs6863338 | T | C | 0.2553 | 0.05018 | 3.85E-07 | 0.0116 | 0.0277 | 0.674899 |
|  | rs74856421 | T | C | 0.22 | 0.04913 | 7.79E-06 | 0.068 | 0.0348 | 0.05082 |
|  | rs75215140 | G | A | -0.1632 | 0.0367 | 9.04E-06 | -0.0061 | 0.0249 | 0.8076 |
|  | rs7575742 | G | A | 0.1383 | 0.02791 | 7.57E-07 | -0.0221 | 0.0192 | 0.2498 |
|  | rs767896 | T | C | 0.1637 | 0.03376 | 1.31E-06 | 0.0152 | 0.019 | 0.4238 |
|  | rs77154024 | C | T | 1.113 | 0.242 | 4.42E-06 | 0.0168 | 0.0473 | 0.722199 |
|  | rs873220 | A | G | -0.1277 | 0.02879 | 9.57E-06 | 0.0038 | 0.0187 | 0.838 |
|  | rs9912354 | T | C | 0.1471 | 0.02719 | 6.80E-08 | 0.0125 | 0.019 | 0.5126 |
| CD11c on myeloid DC | rs11004890 | A | G | 0.1239 | 9.01E-06 | 0.02786 | -0.0264 | 0.019 | 0.1656 |
|  | rs113167560 | G | T | -0.3213 | 2.12E-06 | 0.06762 | 0.13 | 0.0852 | 0.1268 |
|  | rs113404848 | G | A | 0.2758 | 7.96E-07 | 0.05575 | -0.0215 | 0.087 | 0.8048 |
|  | rs11708131 | A | G | -0.2627 | 9.12E-07 | 0.05339 | 0.0128 | 0.0312 | 0.681201 |
|  | rs13255633 | T | C | 0.1629 | 3.31E-06 | 0.03496 | -0.0076 | 0.0256 | 0.7676 |
|  | rs138069865 | C | T | 1.872 | 3.22E-06 | 0.4013 | 0.0101 | 0.0665 | 0.8788 |
|  | rs148343003 | T | C | 1.711 | 1.91E-06 | 0.3584 | -0.0381 | 0.0397 | 0.3374 |
|  | rs16881169 | G | A | 0.2616 | 9.26E-06 | 0.05889 | -0.0073 | 0.0429 | 0.8647 |
|  | rs17481824 | C | T | 0.1773 | 4.96E-06 | 0.03875 | -4.00E-04 | 0.029 | 0.9887 |
|  | rs2541232 | A | G | 0.1224 | 5.77E-06 | 0.02695 | -0.0417 | 0.0182 | 0.02195 |
|  | rs35945038 | A | G | -0.1261 | 5.18E-06 | 0.02762 | -0.0075 | 0.0196 | 0.7028 |
|  | rs4770411 | C | T | 0.1622 | 5.17E-07 | 0.03224 | -0.0147 | 0.0223 | 0.5106 |
|  | rs56116087 | A | G | -0.2078 | 2.69E-06 | 0.04419 | 0.0234 | 0.0316 | 0.4588 |
|  | rs56210513 | T | C | -0.5557 | 6.90E-06 | 0.1234 | 0.103 | 0.0498 | 0.03873 |
|  | rs61802329 | T | C | 0.1669 | 9.48E-10 | 0.02718 | -0.0058 | 0.0188 | 0.758901 |
|  | rs67898294 | T | C | 0.3276 | 7.19E-31 | 0.02803 | 0.0158 | 0.0265 | 0.552199 |
|  | rs71652355 | A | G | 0.3556 | 3.62E-06 | 0.07661 | -0.0296 | 0.1443 | 0.8376 |
|  | rs72662658 | A | G | 0.5005 | 8.80E-08 | 0.09331 | -0.0998 | 0.0716 | 0.1637 |
|  | rs72732223 | G | A | 0.2036 | 8.27E-06 | 0.04558 | -0.0218 | 0.0478 | 0.6479 |
|  | rs72873714 | A | G | 0.2715 | 6.61E-06 | 0.06014 | -0.0984 | 0.0476 | 0.0389 |
|  | rs75841521 | A | G | -0.442 | 4.65E-08 | 0.08067 | 0.021 | 0.1552 | 0.8924 |
|  | rs778233 | T | C | -0.1335 | 1.06E-06 | 0.0273 | -0.0053 | 0.0185 | 0.7734 |
|  | rs7795869 | G | A | 0.1616 | 3.27E-06 | 0.03467 | -0.0473 | 0.0294 | 0.1078 |
|  | rs78520150 | A | C | 0.3947 | 3.57E-08 | 0.07143 | 0.0255 | 0.0556 | 0.6468 |
|  | rs7869020 | C | T | -0.2428 | 1.46E-06 | 0.05031 | -0.0256 | 0.0563 | 0.649101 |
|  | rs79298699 | T | C | -0.279 | 7.22E-09 | 0.04807 | -0.0077 | 0.0303 | 0.7987 |
|  | rs79355481 | T | C | 0.383 | 2.08E-07 | 0.0736 | -0.0956 | 0.0545 | 0.07938 |
|  | rs79968431 | A | G | 0.9865 | 4.45E-07 | 0.1949 | 0.0814 | 0.09 | 0.3658 |
|  | rs80318951 | T | C | -0.1501 | 2.54E-07 | 0.02905 | 0.0016 | 0.0186 | 0.9324 |
|  | rs9562128 | G | A | 0.1343 | 5.80E-06 | 0.02956 | -0.001 | 0.0192 | 0.9603 |
| HLA DR on plasmacytoid DC | rs10812812 | A | T | -0.2691 | 6.55E-06 | 0.0596 | -0.003 | 0.0238 | 0.901 |
|  | rs115770777 | T | C | 0.1972 | 7.66E-06 | 0.04399 | 0.0555 | 0.0484 | 0.2518 |
|  | rs116007826 | G | A | 0.751 | 7.61E-34 | 0.06113 | 0.0102 | 0.317 | 0.9743 |
|  | rs11615028 | G | A | 1.511 | 5.38E-06 | 0.3314 | -0.0624 | 0.0474 | 0.188 |
|  | rs11615578 | T | C | -0.1535 | 9.16E-06 | 0.03454 | 0.0063 | 0.0219 | 0.775001 |
|  | rs11644019 | T | C | -0.291 | 1.10E-13 | 0.03898 | -0.0048 | 0.0225 | 0.8313 |
|  | rs11731221 | G | C | -0.141 | 8.79E-07 | 0.02861 | 0.0108 | 0.0208 | 0.6029 |
|  | rs12607423 | T | C | -0.1621 | 2.71E-06 | 0.03448 | -0.0031 | 0.0211 | 0.8846 |
|  | rs144816332 | A | G | -0.8192 | 4.97E-06 | 0.1791 | 0.072 | 0.0878 | 0.4118 |
|  | rs146863538 | C | A | 1.974 | 1.07E-06 | 0.4037 | -0.1259 | 0.1288 | 0.3285 |
|  | rs1646067 | A | G | 0.1336 | 8.13E-06 | 0.02988 | -0.0204 | 0.0192 | 0.2872 |
|  | rs16890461 | T | C | 0.2128 | 2.06E-06 | 0.04473 | 0.0598 | 0.0281 | 0.0337 |
|  | rs16894020 | A | G | 0.5556 | 8.11E-07 | 0.1124 | -0.0805 | 0.0626 | 0.1981 |
|  | rs200819 | G | A | -0.3651 | 9.57E-06 | 0.08233 | -0.0067 | 0.0733 | 0.9275 |
|  | rs2496370 | A | T | -0.911 | 9.62E-06 | 0.2055 | 0.0661 | 0.1039 | 0.5243 |
|  | rs29221 | G | C | 0.3186 | 4.83E-15 | 0.04046 | -0.0217 | 0.0201 | 0.2805 |
|  | rs34039593 | G | T | 1.217 | 6.25E-146 | 0.0446 | -0.0811 | 0.0256 | 0.001562 |
|  | rs7204799 | G | C | 0.5416 | 4.00E-11 | 0.0817 | 0.0196 | 0.0372 | 0.5971 |
|  | rs7748976 | A | G | 0.2325 | 1.28E-08 | 0.04076 | 0.0023 | 0.0202 | 0.9105 |
|  | rs9267650 | T | A | -0.3903 | 7.31E-26 | 0.03676 | 0.0205 | 0.06 | 0.7323 |
|  | rs9296042 | T | C | -0.294 | 8.65E-22 | 0.03041 | 0.0337 | 0.0208 | 0.1059 |
|  | rs952839 | A | G | 0.538 | 2.44E-06 | 0.1139 | -0.0045 | 0.1045 | 0.9656 |
| HLA DR on DC | rs11251654 | A | G | 0.1804 | 0.0401 | 7.13E-06 | 0.0021 | 0.0266 | 0.9385 |
|  | rs116007826 | G | A | 0.8336 | 0.06047 | 6.52E-42 | 0.0102 | 0.317 | 0.9743 |
|  | rs11644019 | T | C | -0.1978 | 0.04003 | 8.18E-07 | -0.0048 | 0.0225 | 0.8313 |
|  | rs117673964 | T | A | 0.5167 | 0.1143 | 6.42E-06 | 0.0396 | 0.0646 | 0.540201 |
|  | rs12140470 | A | C | -0.1759 | 0.03703 | 2.13E-06 | 0.0083 | 0.021 | 0.693201 |
|  | rs13064098 | T | C | -0.1564 | 0.03257 | 1.66E-06 | -0.0492 | 0.0227 | 0.02992 |
|  | rs140657974 | A | G | 0.7849 | 0.1496 | 1.67E-07 | 0.1365 | 0.151 | 0.3661 |
|  | rs149978880 | T | C | -0.4401 | 0.0994 | 9.90E-06 | 0.1071 | 0.1202 | 0.373 |
|  | rs17794301 | G | C | 1.097 | 0.235 | 3.16E-06 | 0.0638 | 0.075 | 0.3949 |
|  | rs191565346 | A | G | 0.9225 | 0.1971 | 3.01E-06 | -0.2028 | 0.1581 | 0.1996 |
|  | rs34039593 | G | T | 1.075 | 0.04613 | 3.67E-110 | -0.0811 | 0.0256 | 0.001562 |
|  | rs362522 | T | C | 0.3225 | 0.04073 | 3.42E-15 | -0.0211 | 0.0202 | 0.2959 |
|  | rs4085480 | A | G | 0.3553 | 0.07792 | 5.34E-06 | 0.0468 | 0.0357 | 0.1906 |
|  | rs55971447 | T | C | -0.3418 | 0.03192 | 2.91E-26 | -0.0044 | 0.0298 | 0.8822 |
|  | rs66680090 | G | A | -0.1532 | 0.03417 | 7.64E-06 | 0.0076 | 0.0223 | 0.7322 |
|  | rs7204799 | G | C | 0.4473 | 0.08344 | 8.96E-08 | 0.0196 | 0.0372 | 0.5971 |
|  | rs7748976 | A | G | 0.2659 | 0.04133 | 1.44E-10 | 0.0023 | 0.0202 | 0.9105 |
|  | rs9267650 | T | A | -0.3215 | 0.0367 | 3.25E-18 | 0.0205 | 0.06 | 0.7323 |
|  | rs9296042 | T | C | -0.3191 | 0.03045 | 3.02E-25 | 0.0337 | 0.0208 | 0.1059 |
| HLA DR on CD33- HLA DR+ | rs114068468 | T | C | 0.3671 | 2.51E-08 | 0.06556 | -0.0941 | 0.128 | 0.4622 |
|  | rs11576012 | T | C | 0.4345 | 1.32E-07 | 0.08199 | 0.045 | 0.0463 | 0.3315 |
|  | rs140697652 | C | T | 0.5782 | 5.42E-06 | 0.1267 | -0.1972 | 0.2165 | 0.3623 |
|  | rs1474618 | A | C | -0.1723 | 3.19E-06 | 0.03685 | 0.0096 | 0.02 | 0.6323 |
|  | rs2760980 | A | G | 1.049 | 2.44E-60 | 0.06137 | -0.0805 | 0.0256 | 0.001677 |
|  | rs775443 | T | C | -0.509 | 1.64E-06 | 0.1058 | -0.0019 | 0.034 | 0.9551 |
|  | rs78909221 | G | A | -1.314 | 7.37E-06 | 0.2921 | -0.0542 | 0.0561 | 0.334 |
|  | rs9268430 | C | A | -0.4503 | 4.34E-23 | 0.04481 | 0.0154 | 0.0345 | 0.6549 |
|  | rs9279789 | C | T | 0.7114 | 2.87E-10 | 0.1121 | -0.1287 | 0.1327 | 0.332 |
|  | rs928189 | C | T | -0.1864 | 3.29E-06 | 0.03993 | 0.0034 | 0.0194 | 0.8606 |
|  | rs9310124 | C | A | -0.1783 | 1.03E-06 | 0.03636 | 0.0035 | 0.0194 | 0.8577 |
|  | rs9368784 | T | G | 0.3138 | 2.84E-07 | 0.06086 | -0.0013 | 0.0206 | 0.9509 |
|  | rs9545905 | G | A | 0.1708 | 5.72E-06 | 0.03753 | -0.0338 | 0.0183 | 0.06381 |

**Table S2** **Full result of MR estimates for the association between Immune Cells and Her2- Breast Cancer.**

| Immune Cells | MR. Method | No. of SNP | b | se | or | 95% CI | pval | P_FDR_ |
| --- | --- | --- | --- | --- | --- | --- | --- | --- |
| Activated & secreting Treg %CD4+ | MR Egger | 31 | -0.03 | 0.01 | 0.97 | 0.96 - 0.99 | 0.01 |  |
|  | Weighted median | 31 | -0.02 | 0.01 | 0.98 | 0.95 - 1.00 | 0.04 |  |
|  | Inverse variance weighted | 31 | -0.03 | 0.01 | 0.97 | 0.96 - 0.99 | 0.00 | 0.031 |
|  | Simple mode | 31 | -0.02 | 0.02 | 0.98 | 0.95 - 1.02 | 0.35 |  |
|  | Weighted mode | 31 | -0.02 | 0.01 | 0.98 | 0.96 - 1.00 | 0.02 |  |
| HLA DR on plasmacytoid DC | MR Egger | 22 | -0.06 | 0.02 | 0.94 | 0.90 - 0.98 | 0.00 |  |
|  | Weighted median | 22 | -0.07 | 0.02 | 0.94 | 0.90 - 0.97 | 0.00 |  |
|  | Inverse variance weighted | 22 | -0.05 | 0.01 | 0.95 | 0.93 - 0.98 | 0.00 | 0.024 |
|  | Simple mode | 22 | -0.05 | 0.03 | 0.95 | 0.89 - 1.02 | 0.15 |  |
|  | Weighted mode | 22 | -0.06 | 0.02 | 0.94 | 0.91 - 0.98 | 0.00 |  |

**Table S3 The heterogeneity of Immune Cells instrumental variables (IVW).**

| Immune Cells (exposure) | Q | Q_df | Q_pval |
| --- | --- | --- | --- |
| Activated & secreting Treg %CD4+ | 23.74 | 30 | 0.78 |
| HLA DR on plasmacytoid DC | 14.91 | 21 | 0.83 |

**Table S4 Directional horizontal pleiotropy assessed by intercept term in MR Egger regression of the association between immune cells and Her2- breast cancer.**

| IC (exposure) | Egger_intercept | se | pval |
| --- | --- | --- | --- |
| Activated & secreting Treg %CD4+ | -0.000146 | 0.006906 | 0.983244 |
| HLA DR on plasmacytoid DC | 0.01 | 0.009037 | 0.281619 |

**Table S5 MR-PRESSO analysis for the association between immune cells and Her2- breast cancer.**

| Immune Cells (exposure) | Causal Estimate | SD | T | P-value | RSS_obs_ | Global test P-value | Outlier-corrected |
| --- | --- | --- | --- | --- | --- | --- | --- |
| Activated & secreting Treg %CD4+ | -0.026257 | 0.006837 | -3.840521 | 0.000591 | 25.16373 | 0.83 | NA |
| HLA DR on plasmacytoid DC | -0.048269 | 0.011663 | -4.138696 | 0.000466 | 17.05573 | 0.817 | NA |

**TABLE S6 Results based on MAGMA**

| GENE | CHR | START | STOP | NSNPS | NPARAM | N | ZSTAT | P |
| --- | --- | --- | --- | --- | --- | --- | --- | --- |
| 84250 | 5 | 94615858 | 94714918 | 1 | 1 | 123579 | 3.4565 | 0.000274 |
| 79772 | 5 | 94703690 | 95284575 | 1 | 1 | 123579 | 3.4565 | 0.000274 |
| 10588 | 15 | 79843547 | 79897285 | 1 | 1 | 123579 | 3.2389 | 0.0006 |
| 1.01E+08 | 15 | 79843547 | 79923106 | 1 | 1 | 123579 | 3.2389 | 0.0006 |
| 83473 | 18 | 46917602 | 47102243 | 1 | 1 | 123579 | 2.979 | 0.001446 |
| 146760 | 17 | 1934677 | 2025345 | 1 | 1 | 123579 | 2.5891 | 0.004811 |
| 10973 | 6 | 1.01E+08 | 1.01E+08 | 1 | 1 | 123579 | 2.576 | 0.004997 |
| 56940 | 6 | 292057 | 351355 | 1 | 1 | 123579 | 2.5548 | 0.005313 |
| 1.05E+08 | 6 | 9845518 | 10159438 | 1 | 1 | 123579 | 2.5451 | 0.005463 |
| 348180 | 16 | 88706483 | 88715386 | 1 | 1 | 123579 | 2.5255 | 0.005776 |
| 2131 | 8 | 1.18E+08 | 1.18E+08 | 1 | 1 | 123579 | 2.5051 | 0.00612 |
| 114882 | 12 | 76351797 | 76559809 | 1 | 1 | 123579 | 2.4932 | 0.00633 |
| 4126 | 4 | 1.03E+08 | 1.03E+08 | 1 | 1 | 123579 | 2.3977 | 0.008249 |
| 3708 | 3 | 4493348 | 4847840 | 1 | 1 | 123579 | 2.3044 | 0.0106 |
| 2099 | 6 | 1.52E+08 | 1.52E+08 | 1 | 1 | 123579 | 2.2678 | 0.01167 |
| 57544 | 14 | 52430590 | 52552583 | 1 | 1 | 123579 | 2.2218 | 0.01315 |
| 64063 | 16 | 2852727 | 2858630 | 1 | 1 | 123579 | 2.1675 | 0.0151 |
| 51496 | 15 | 44427329 | 44527257 | 1 | 1 | 123579 | 2.1656 | 0.01517 |
| 347731 | 10 | 66926034 | 67101551 | 1 | 1 | 123579 | 2.153 | 0.01566 |
| 60495 | 10 | 98457077 | 99235875 | 1 | 1 | 123579 | 2.1205 | 0.01698 |
| 285423 | 4 | 1.49E+08 | 1.5E+08 | 1 | 1 | 123579 | 2.0486 | 0.02025 |
| 10367 | 10 | 72367326 | 72626191 | 1 | 1 | 123579 | 2.015 | 0.02195 |
| 6401 | 1 | 1.7E+08 | 1.7E+08 | 1 | 1 | 123579 | 2.0024 | 0.02262 |
| 22869 | 9 | 96754553 | 96778119 | 1 | 1 | 123579 | 1.9998 | 0.02276 |
| 5690 | 1 | 35599541 | 35641844 | 1 | 1 | 123579 | 1.9027 | 0.02854 |
| 29 | 17 | 1003518 | 1187322 | 1 | 1 | 123579 | 1.8989 | 0.02879 |
| 85379 | 22 | 24952702 | 25197448 | 2 | 1 | 123579 | 1.8785 | 0.030159 |
| 25814 | 22 | 45671798 | 45845307 | 1 | 1 | 123579 | 1.842 | 0.03274 |
| 4208 | 5 | 88718241 | 88904105 | 1 | 1 | 123579 | 1.8361 | 0.03317 |
| 2153 | 1 | 1.7E+08 | 1.7E+08 | 1 | 1 | 123579 | 1.8101 | 0.03514 |
| 164091 | 1 | 25861484 | 25876707 | 1 | 1 | 123579 | 1.7636 | 0.0389 |
| 8671 | 4 | 71187286 | 71572087 | 1 | 1 | 123579 | 1.7585 | 0.03933 |
| 29119 | 10 | 65912518 | 67763610 | 3 | 2 | 123579 | 1.7411 | 0.040835 |
| 1.01E+08 | 2 | 1.7E+08 | 1.7E+08 | 1 | 1 | 123579 | 1.7143 | 0.04324 |
| 3069 | 2 | 2.41E+08 | 2.41E+08 | 1 | 1 | 123579 | 1.699 | 0.04466 |
| 1.02E+08 | 2 | 2.41E+08 | 2.41E+08 | 1 | 1 | 123579 | 1.699 | 0.04466 |
| 9722 | 1 | 1.62E+08 | 1.62E+08 | 2 | 1 | 123579 | 1.6894 | 0.045572 |
| 79098 | 1 | 2.07E+08 | 2.07E+08 | 1 | 1 | 123579 | 1.6538 | 0.049079 |
| 1387 | 16 | 3725054 | 3880120 | 1 | 1 | 123579 | 1.637 | 0.050819 |
| 4009 | 1 | 1.65E+08 | 1.65E+08 | 1 | 1 | 123579 | 1.6189 | 0.05273 |
| 81 | 19 | 38647616 | 38730531 | 1 | 1 | 123579 | 1.5985 | 0.05496 |
| 2762 | 6 | 1623800 | 2245634 | 1 | 1 | 123579 | 1.5854 | 0.05644 |
| 64131 | 16 | 17102324 | 17470881 | 1 | 1 | 123579 | 1.5808 | 0.05696 |
| 114793 | 2 | 1.52E+08 | 1.53E+08 | 1 | 1 | 123579 | 1.5551 | 0.05996 |
| 58513 | 19 | 16355244 | 16472012 | 1 | 1 | 123579 | 1.5529 | 0.06022 |
| 40 | 17 | 33013087 | 34156806 | 3 | 2 | 123579 | 1.5514 | 0.060402 |
| 1528 | 18 | 74253292 | 74292016 | 1 | 1 | 123579 | 1.5289 | 0.063149 |
| 673 | 7 | 1.41E+08 | 1.41E+08 | 1 | 1 | 123579 | 1.5202 | 0.06423 |
| 54360 | 4 | 5014586 | 5019470 | 1 | 1 | 123579 | 1.4674 | 0.07113 |
| 7726 | 6 | 30184453 | 30213494 | 1 | 1 | 123579 | 1.456 | 0.072699 |
| 1956 | 7 | 55019032 | 55207338 | 1 | 1 | 123579 | 1.4273 | 0.07674 |
| 4745 | 11 | 20669551 | 21575686 | 2 | 1 | 123579 | 1.4106 | 0.079184 |
| 5538 | 1 | 40072710 | 40097470 | 1 | 1 | 123579 | 1.4093 | 0.07938 |
| 64478 | 8 | 2935353 | 4994806 | 2 | 1 | 123579 | 1.4081 | 0.079544 |
| 23112 | 22 | 40044817 | 40335808 | 1 | 1 | 123579 | 1.391 | 0.08212 |
| 5527 | 14 | 1.02E+08 | 1.02E+08 | 1 | 1 | 123579 | 1.3887 | 0.082459 |
| 10656 | 8 | 1.35E+08 | 1.36E+08 | 1 | 1 | 123579 | 1.3705 | 0.085271 |
| 79805 | 1 | 2.13E+08 | 2.13E+08 | 1 | 1 | 123579 | 1.3571 | 0.08738 |
| 3781 | 5 | 1.14E+08 | 1.14E+08 | 1 | 1 | 123579 | 1.3532 | 0.087989 |
| 23173 | 4 | 98995637 | 99062809 | 1 | 1 | 123579 | 1.3416 | 0.089861 |
| 1767 | 5 | 13690328 | 14011759 | 1 | 1 | 123579 | 1.3248 | 0.092619 |
| 9727 | 16 | 425592 | 522485 | 1 | 1 | 123579 | 1.2983 | 0.097089 |
| 253582 | 6 | 1.3E+08 | 1.3E+08 | 1 | 1 | 123579 | 1.2873 | 0.099001 |
| 10718 | 10 | 81875309 | 82987179 | 2 | 1 | 123579 | 1.272 | 0.10168 |
| 11260 | 12 | 64404350 | 64451125 | 1 | 1 | 123579 | 1.2708 | 0.1019 |
| 2567 | 15 | 26971282 | 27533895 | 1 | 1 | 123579 | 1.2669 | 0.1026 |
| 6789 | 20 | 44966479 | 45079975 | 1 | 1 | 123579 | 1.2635 | 0.1032 |
| 10006 | 10 | 26746596 | 26861087 | 1 | 1 | 123579 | 1.2574 | 0.1043 |
| 7273 | 2 | 1.79E+08 | 1.79E+08 | 1 | 1 | 123579 | 1.2558 | 0.1046 |
| 10369 | 22 | 36560869 | 36702645 | 1 | 1 | 123579 | 1.2459 | 0.1064 |
| 56967 | 14 | 96039324 | 96093971 | 1 | 1 | 123579 | 1.2426 | 0.107 |
| 313 | 7 | 36512941 | 36724549 | 1 | 1 | 123579 | 1.2383 | 0.1078 |
| 63976 | 1 | 3068227 | 3438621 | 1 | 1 | 123579 | 1.2356 | 0.1083 |
| 84254 | 17 | 3860315 | 3894891 | 1 | 1 | 123579 | 1.2196 | 0.1113 |
| 22926 | 1 | 1.62E+08 | 1.62E+08 | 1 | 1 | 123579 | 1.2181 | 0.1116 |
| 9958 | 12 | 62260340 | 62409721 | 1 | 1 | 123579 | 1.2092 | 0.1133 |
| 2222 | 8 | 11795573 | 11839309 | 1 | 1 | 123579 | 1.1988 | 0.1153 |
| 919 | 1 | 1.67E+08 | 1.68E+08 | 4 | 1 | 123579 | 1.18 | 0.119 |
| 91133 | 18 | 5954703 | 6414911 | 2 | 1 | 123579 | 1.1707 | 0.12086 |
| 340706 | 10 | 1.14E+08 | 1.14E+08 | 1 | 1 | 123579 | 1.1542 | 0.1242 |
| 149954 | 20 | 33081512 | 33111751 | 1 | 1 | 123579 | 1.125 | 0.1303 |
| 83856 | 9 | 1.05E+08 | 1.06E+08 | 1 | 1 | 123579 | 1.1189 | 0.1316 |
| 11052 | 12 | 69239537 | 69274358 | 1 | 1 | 123579 | 1.1142 | 0.1326 |
| 9745 | 19 | 30225044 | 30713538 | 1 | 1 | 123579 | 1.0957 | 0.1366 |
| 170679 | 6 | 31114831 | 31140092 | 1 | 1 | 123579 | 1.0781 | 0.1405 |
| 1041 | 6 | 31115088 | 31120475 | 1 | 1 | 123579 | 1.0781 | 0.1405 |
| 65217 | 10 | 53802771 | 54801291 | 3 | 2 | 123579 | 1.0506 | 0.14672 |
| 5074 | 12 | 79587791 | 79691010 | 1 | 1 | 123579 | 1.0296 | 0.1516 |
| 57509 | 8 | 17643794 | 17801220 | 1 | 1 | 123579 | 1.0241 | 0.1529 |
| 728734 | 16 | 28624517 | 28658725 | 1 | 1 | 123579 | 1.0203 | 0.1538 |
| 115 | 16 | 3953387 | 4116185 | 1 | 1 | 123579 | 1.0169 | 0.1546 |
| 321 | 15 | 28885994 | 29118315 | 1 | 1 | 123579 | 1.014 | 0.1553 |
| 10320 | 7 | 50303465 | 50405101 | 1 | 1 | 123579 | 1.0064 | 0.1571 |
| 64641 | 8 | 25841730 | 26045124 | 1 | 1 | 123579 | 1.0027 | 0.158 |
| 85440 | 1 | 62454726 | 62688368 | 2 | 1 | 123579 | 0.99684 | 0.15942 |
| 962 | 1 | 1.61E+08 | 1.61E+08 | 1 | 1 | 123579 | 0.99446 | 0.16 |
| 117145 | 1 | 1.52E+08 | 1.52E+08 | 1 | 1 | 123579 | 0.98995 | 0.1611 |
| 126859 | 1 | 1.79E+08 | 1.8E+08 | 1 | 1 | 123579 | 0.98261 | 0.1629 |
| 3305 | 6 | 31809619 | 31821999 | 1 | 1 | 123579 | 0.9822 | 0.163 |
| 7812 | 1 | 1.15E+08 | 1.15E+08 | 1 | 1 | 123579 | 0.9725 | 0.1654 |
| 9353 | 4 | 20251925 | 20620561 | 1 | 1 | 123579 | 0.9629 | 0.1678 |
| 84335 | 19 | 49869033 | 49878356 | 1 | 1 | 123579 | 0.93731 | 0.1743 |
| 5789 | 9 | 8314246 | 10612723 | 2 | 1 | 123579 | 0.93094 | 0.17594 |
| 23456 | 1 | 2.3E+08 | 2.3E+08 | 1 | 1 | 123579 | 0.92724 | 0.1769 |
| 83478 | 4 | 85147742 | 86002670 | 2 | 1 | 123579 | 0.91222 | 0.18083 |
| 10392 | 7 | 30424527 | 30478797 | 1 | 1 | 123579 | 0.90588 | 0.1825 |
| 29117 | 16 | 50319018 | 50368966 | 1 | 1 | 123579 | 0.90135 | 0.1837 |
| 6532 | 17 | 30194319 | 30235968 | 1 | 1 | 123579 | 0.8991 | 0.1843 |
| 10090 | 6 | 1.49E+08 | 1.49E+08 | 1 | 1 | 123579 | 0.8961 | 0.1851 |
| 653583 | 19 | 43474341 | 43504920 | 2 | 1 | 123579 | 0.88601 | 0.18781 |
| 1272 | 12 | 40692442 | 41072412 | 1 | 1 | 123579 | 0.88529 | 0.188 |
| 5451 | 1 | 1.67E+08 | 1.67E+08 | 2 | 1 | 123579 | 0.88466 | 0.18817 |
| 7186 | 9 | 1.37E+08 | 1.37E+08 | 1 | 1 | 123579 | 0.8827 | 0.1887 |
| 3036 | 19 | 51713112 | 51723992 | 1 | 1 | 123579 | 0.8658 | 0.1933 |
| 90957 | 2 | 38797729 | 38875883 | 1 | 1 | 123579 | 0.86507 | 0.1935 |
| 4807 | 1 | 1.6E+08 | 1.6E+08 | 1 | 1 | 123579 | 0.85419 | 0.1965 |
| 1314 | 1 | 1.6E+08 | 1.6E+08 | 1 | 1 | 123579 | 0.85419 | 0.1965 |
| 64288 | 6 | 28312837 | 28354506 | 1 | 1 | 123579 | 0.84843 | 0.1981 |
| 154664 | 7 | 48171448 | 48647497 | 1 | 1 | 123579 | 0.84592 | 0.1988 |
| 1.01E+08 | 1 | 1.2E+08 | 1.21E+08 | 1 | 1 | 123579 | 0.83379 | 0.2022 |
| 11073 | 3 | 1.34E+08 | 1.34E+08 | 1 | 1 | 123579 | 0.82883 | 0.2036 |
| 138881 | 9 | 1.23E+08 | 1.23E+08 | 1 | 1 | 123579 | 0.82812 | 0.2038 |
| 54454 | 2 | 23685229 | 23927114 | 1 | 1 | 123579 | 0.81059 | 0.2088 |
| 5800 | 12 | 15322257 | 15598331 | 1 | 1 | 123579 | 0.78919 | 0.215 |
| 27159 | 1 | 1.11E+08 | 1.11E+08 | 1 | 1 | 123579 | 0.78271 | 0.2169 |
| 9765 | 5 | 80407592 | 80480520 | 1 | 1 | 123579 | 0.75109 | 0.2263 |
| 51426 | 5 | 75511043 | 75599823 | 1 | 1 | 123579 | 0.74347 | 0.2286 |
| 83547 | 17 | 1646150 | 1650098 | 1 | 1 | 123579 | 0.74214 | 0.229 |
| 5101 | 13 | 66302834 | 67230336 | 1 | 1 | 123579 | 0.74181 | 0.2291 |
| 388336 | 17 | 11241417 | 11564063 | 1 | 1 | 123579 | 0.73293 | 0.2318 |
| 4438 | 1 | 75796871 | 75913238 | 1 | 1 | 123579 | 0.72868 | 0.2331 |
| 7049 | 1 | 91680343 | 91906002 | 1 | 1 | 123579 | 0.7063 | 0.24 |
| 5922 | 3 | 1.41E+08 | 1.42E+08 | 1 | 1 | 123579 | 0.7063 | 0.24 |
| 79839 | 18 | 68715254 | 69171081 | 1 | 1 | 123579 | 0.68999 | 0.2451 |
| 84083 | 2 | 1.35E+08 | 1.36E+08 | 1 | 1 | 123579 | 0.67512 | 0.2498 |
| 11142 | 20 | 44531781 | 44619037 | 1 | 1 | 123579 | 0.66477 | 0.2531 |
| 51741 | 16 | 78099413 | 79212667 | 4 | 2 | 123579 | 0.65541 | 0.2561 |
| 1021 | 7 | 92604921 | 92836627 | 1 | 1 | 123579 | 0.65386 | 0.2566 |
| 201134 | 17 | 65635540 | 66192131 | 1 | 1 | 123579 | 0.65107 | 0.2575 |
| 5890 | 14 | 67819779 | 68683210 | 1 | 1 | 123579 | 0.65045 | 0.2577 |
| 2590 | 1 | 2.3E+08 | 2.3E+08 | 1 | 1 | 123579 | 0.61675 | 0.2687 |
| 23446 | 9 | 1.05E+08 | 1.05E+08 | 1 | 1 | 123579 | 0.61342 | 0.2698 |
| 6092 | 3 | 75906675 | 77649964 | 1 | 1 | 123579 | 0.60046 | 0.2741 |
| 1793 | 10 | 1.27E+08 | 1.27E+08 | 1 | 1 | 123579 | 0.59477 | 0.276 |
| 84125 | 12 | 85036321 | 85272867 | 1 | 1 | 123579 | 0.59417 | 0.2762 |
| 1370 | 3 | 1.94E+08 | 1.94E+08 | 1 | 1 | 123579 | 0.58879 | 0.278 |
| 7932 | 6 | 29587378 | 29591012 | 1 | 1 | 123579 | 0.58136 | 0.2805 |
| 1305 | 10 | 69801863 | 69959148 | 1 | 1 | 123579 | 0.57721 | 0.2819 |
| 50863 | 11 | 1.31E+08 | 1.32E+08 | 1 | 1 | 123579 | 0.57602 | 0.2823 |
| 472 | 11 | 1.08E+08 | 1.08E+08 | 1 | 1 | 123579 | 0.56894 | 0.2847 |
| 160140 | 11 | 1.08E+08 | 1.08E+08 | 1 | 1 | 123579 | 0.56894 | 0.2847 |
| 10207 | 1 | 61742477 | 62163920 | 1 | 1 | 123579 | 0.56423 | 0.2863 |
| 23189 | 9 | 470294 | 746106 | 1 | 1 | 123579 | 0.561 | 0.2874 |
| 66005 | 11 | 867859 | 915058 | 1 | 1 | 123579 | 0.54639 | 0.2924 |
| 3123 | 6 | 32578769 | 32589836 | 2 | 1 | 123579 | 0.54209 | 0.29388 |
| 11332 | 1 | 6264272 | 6393766 | 1 | 1 | 123579 | 0.53132 | 0.2976 |
| 55083 | 1 | 2.45E+08 | 2.46E+08 | 1 | 1 | 123579 | 0.52872 | 0.2985 |
| 57661 | 11 | 576220 | 612222 | 1 | 1 | 123579 | 0.52354 | 0.3003 |
| 577 | 6 | 68635282 | 69389511 | 2 | 1 | 123579 | 0.52334 | 0.30037 |
| 1E+08 | 2 | 38919363 | 38975449 | 3 | 1 | 123579 | 0.52215 | 0.30078 |
| 80336 | 20 | 44910058 | 44958801 | 1 | 1 | 123579 | 0.51866 | 0.302 |
| 6091 | 3 | 78597238 | 79767998 | 1 | 1 | 123579 | 0.50466 | 0.3069 |
| 860 | 6 | 45327800 | 45664032 | 1 | 1 | 123579 | 0.49982 | 0.3086 |
| 1794 | 5 | 1.7E+08 | 1.7E+08 | 1 | 1 | 123579 | 0.49812 | 0.3092 |
| 10762 | 22 | 45163845 | 45188011 | 2 | 1 | 123579 | 0.49289 | 0.31104 |
| 59343 | 3 | 1.86E+08 | 1.86E+08 | 1 | 1 | 123579 | 0.45265 | 0.3254 |
| 4800 | 6 | 41072968 | 41102407 | 1 | 1 | 123579 | 0.44794 | 0.3271 |
| 5079 | 9 | 36833275 | 37035319 | 1 | 1 | 123579 | 0.44794 | 0.3271 |
| 54815 | 19 | 19385833 | 19508932 | 1 | 1 | 123579 | 0.44406 | 0.3285 |
| 9218 | 18 | 9913958 | 9960021 | 1 | 1 | 123579 | 0.4424 | 0.3291 |
| 29903 | 19 | 55647568 | 55653161 | 1 | 1 | 123579 | 0.44212 | 0.3292 |
| 51149 | 5 | 1.8E+08 | 1.8E+08 | 1 | 1 | 123579 | 0.44102 | 0.3296 |
| 4154 | 3 | 1.52E+08 | 1.52E+08 | 1 | 1 | 123579 | 0.44047 | 0.3298 |
| 23198 | 2 | 53864067 | 53970840 | 1 | 1 | 123579 | 0.43771 | 0.3308 |
| 285555 | 4 | 97441146 | 98143470 | 1 | 1 | 123579 | 0.43605 | 0.3314 |
| 7407 | 6 | 31777518 | 31795935 | 1 | 1 | 123579 | 0.43577 | 0.3315 |
| 55608 | 13 | 1.11E+08 | 1.11E+08 | 1 | 1 | 123579 | 0.43302 | 0.3325 |
| 55113 | 1 | 27959462 | 27968096 | 1 | 1 | 123579 | 0.43137 | 0.3331 |
| 56997 | 1 | 2.27E+08 | 2.27E+08 | 1 | 1 | 123579 | 0.42944 | 0.3338 |
| 54436 | 4 | 8182072 | 8241103 | 1 | 1 | 123579 | 0.42889 | 0.334 |
| 79591 | 10 | 1.02E+08 | 1.02E+08 | 1 | 1 | 123579 | 0.41957 | 0.3374 |
| 57097 | 12 | 3808861 | 3873448 | 1 | 1 | 123579 | 0.41164 | 0.3403 |
| 285220 | 3 | 96814581 | 97764977 | 1 | 1 | 123579 | 0.41083 | 0.3406 |
| 6935 | 10 | 31318783 | 31529814 | 1 | 1 | 123579 | 0.391 | 0.3479 |
| 54221 | 2 | 950868 | 1367645 | 1 | 1 | 123579 | 0.3864 | 0.3496 |
| 80012 | 3 | 1.7E+08 | 1.7E+08 | 1 | 1 | 123579 | 0.38181 | 0.3513 |
| 4094 | 16 | 79212772 | 79600786 | 1 | 1 | 123579 | 0.37535 | 0.3537 |
| 83637 | 7 | 44748547 | 44769881 | 1 | 1 | 123579 | 0.37481 | 0.3539 |
| 5343 | 2 | 87010464 | 87021846 | 1 | 1 | 123579 | 0.3622 | 0.3586 |
| 4750 | 4 | 1.69E+08 | 1.7E+08 | 1 | 1 | 123579 | 0.36087 | 0.3591 |
| 4921 | 1 | 1.63E+08 | 1.63E+08 | 1 | 1 | 123579 | 0.34939 | 0.3634 |
| 374786 | 17 | 29929856 | 30108452 | 1 | 1 | 123579 | 0.34353 | 0.3656 |
| 8224 | 22 | 32508022 | 33058391 | 1 | 1 | 123579 | 0.33 | 0.3707 |
| 4026 | 3 | 1.88E+08 | 1.89E+08 | 1 | 1 | 123579 | 0.31917 | 0.3748 |
| 10391 | 15 | 68559275 | 68727806 | 1 | 1 | 123579 | 0.31021 | 0.3782 |
| 9753 | 6 | 28378821 | 28399767 | 1 | 1 | 123579 | 0.29814 | 0.3828 |
| 6653 | 11 | 1.21E+08 | 1.22E+08 | 1 | 1 | 123579 | 0.2861 | 0.3874 |
| 10749 | 17 | 4997948 | 5028399 | 1 | 1 | 123579 | 0.28427 | 0.3881 |
| 9605 | 16 | 89707133 | 89721272 | 1 | 1 | 123579 | 0.28036 | 0.3896 |
| 3664 | 1 | 2.1E+08 | 2.1E+08 | 1 | 1 | 123579 | 0.27776 | 0.3906 |
| 79646 | 5 | 1.69E+08 | 1.69E+08 | 1 | 1 | 123579 | 0.26995 | 0.3936 |
| 148641 | 1 | 2.34E+08 | 2.34E+08 | 1 | 1 | 123579 | 0.26943 | 0.3938 |
| 8745 | 2 | 2.06E+08 | 2.07E+08 | 1 | 1 | 123579 | 0.26501 | 0.3955 |
| 775 | 12 | 1970786 | 2697949 | 2 | 1 | 123579 | 0.25535 | 0.39923 |
| 221188 | 16 | 57529333 | 57577210 | 1 | 1 | 123579 | 0.25309 | 0.4001 |
| 54477 | 12 | 19129692 | 19376400 | 1 | 1 | 123579 | 0.24895 | 0.4017 |
| 84467 | 19 | 8065402 | 8149012 | 1 | 1 | 123579 | 0.24792 | 0.4021 |
| 5579 | 16 | 23835979 | 24220611 | 1 | 1 | 123579 | 0.24456 | 0.4034 |
| 89894 | 12 | 1.12E+08 | 1.12E+08 | 2 | 1 | 123579 | 0.24119 | 0.4047 |
| 285386 | 3 | 1.89E+08 | 1.89E+08 | 1 | 1 | 123579 | 0.23707 | 0.4063 |
| 2894 | 10 | 85599555 | 86366493 | 1 | 1 | 123579 | 0.23012 | 0.409 |
| 4439 | 6 | 31739948 | 31762678 | 1 | 1 | 123579 | 0.22446 | 0.4112 |
| 57475 | 14 | 67533199 | 67589538 | 1 | 1 | 123579 | 0.22292 | 0.4118 |
| 169355 | 8 | 39934955 | 40016391 | 1 | 1 | 123579 | 0.22035 | 0.4128 |
| 114884 | 3 | 31660825 | 31981850 | 1 | 1 | 123579 | 0.20394 | 0.4192 |
| 51738 | 3 | 10285750 | 10292947 | 1 | 1 | 123579 | 0.20292 | 0.4196 |
| 51194 | 5 | 62412746 | 62628589 | 1 | 1 | 123579 | 0.20087 | 0.4204 |
| 9407 | 4 | 67820876 | 67883998 | 1 | 1 | 123579 | 0.17154 | 0.4319 |
| 338645 | 11 | 24496488 | 25082640 | 1 | 1 | 123579 | 0.17103 | 0.4321 |
| 121256 | 12 | 1.29E+08 | 1.3E+08 | 2 | 1 | 123579 | 0.16847 | 0.43311 |
| 50650 | 3 | 56727418 | 57079308 | 1 | 1 | 123579 | 0.16798 | 0.4333 |
| 54620 | 16 | 30923055 | 30948783 | 1 | 1 | 123579 | 0.16595 | 0.4341 |
| 6760 | 18 | 26016253 | 26091212 | 1 | 1 | 123579 | 0.16442 | 0.4347 |
| 10849 | 19 | 45406209 | 45410766 | 1 | 1 | 123579 | 0.16315 | 0.4352 |
| 2067 | 19 | 45407333 | 45478820 | 1 | 1 | 123579 | 0.16315 | 0.4352 |
| 8516 | 10 | 15513949 | 15720335 | 1 | 1 | 123579 | 0.16239 | 0.4355 |
| 729920 | 7 | 15916851 | 16530558 | 1 | 1 | 123579 | 0.15046 | 0.4402 |
| 51095 | 3 | 3126916 | 3152503 | 1 | 1 | 123579 | 0.1421 | 0.4435 |
| 9951 | 16 | 25692026 | 26137688 | 1 | 1 | 123579 | 0.14084 | 0.444 |
| 9586 | 7 | 28299321 | 28825894 | 1 | 1 | 123579 | 0.13805 | 0.4451 |
| 51378 | 20 | 871243 | 916375 | 1 | 1 | 123579 | 0.13805 | 0.4451 |
| 7100 | 1 | 2.23E+08 | 2.23E+08 | 1 | 1 | 123579 | 0.13628 | 0.4458 |
| 9698 | 1 | 30931506 | 31065717 | 1 | 1 | 123579 | 0.12213 | 0.4514 |
| 10512 | 7 | 80742538 | 80919351 | 1 | 1 | 123579 | 0.11102 | 0.4558 |
| 55337 | 19 | 10086119 | 10093252 | 1 | 1 | 123579 | 0.10346 | 0.4588 |
| 91522 | 5 | 1.78E+08 | 1.79E+08 | 1 | 1 | 123579 | 0.099174 | 0.4605 |
| 2272 | 3 | 59747587 | 61251459 | 1 | 1 | 123579 | 0.096655 | 0.4615 |
| 8038 | 10 | 1.26E+08 | 1.26E+08 | 1 | 1 | 123579 | 0.08558 | 0.4659 |
| 344387 | 2 | 39174937 | 39246810 | 1 | 1 | 123579 | 0.080298 | 0.468 |
| 5609 | 19 | 7903780 | 7914483 | 1 | 1 | 123579 | 0.080047 | 0.4681 |
| 55176 | 10 | 12129641 | 12169958 | 1 | 1 | 123579 | 0.078035 | 0.4689 |
| 23499 | 1 | 39081417 | 39487138 | 1 | 1 | 123579 | 0.06547 | 0.4739 |
| 4255 | 10 | 1.29E+08 | 1.3E+08 | 1 | 1 | 123579 | 0.057433 | 0.4771 |
| 64221 | 11 | 1.25E+08 | 1.25E+08 | 1 | 1 | 123579 | 0.046141 | 0.4816 |
| 136227 | 7 | 1.01E+08 | 1.02E+08 | 1 | 1 | 123579 | 0.044884 | 0.4821 |
| 1841 | 2 | 2.42E+08 | 2.42E+08 | 1 | 1 | 123579 | 0.042124 | 0.4832 |
| 6588 | 11 | 1.08E+08 | 1.08E+08 | 1 | 1 | 123579 | 0.035852 | 0.4857 |
| 8379 | 7 | 1815792 | 2232948 | 1 | 1 | 123579 | 0.032341 | 0.4871 |
| 2028 | 4 | 1.1E+08 | 1.11E+08 | 1 | 1 | 123579 | 0.027827 | 0.4889 |
| 5799 | 7 | 1.58E+08 | 1.59E+08 | 1 | 1 | 123579 | 0.014288 | 0.4943 |
| 3189 | 10 | 68332011 | 68343196 | 1 | 1 | 123579 | 0.011029 | 0.4956 |
| 83481 | 8 | 1.44E+08 | 1.44E+08 | 1 | 1 | 123579 | 0.008773 | 0.4965 |
| 7399 | 1 | 2.16E+08 | 2.16E+08 | 1 | 1 | 123579 | 0.007019 | 0.4972 |
| 29915 | 12 | 1.04E+08 | 1.04E+08 | 1 | 1 | 123579 | 0.005013 | 0.498 |
| 6041 | 1 | 1.83E+08 | 1.83E+08 | 1 | 1 | 123579 | 0.003259 | 0.4987 |
| 11174 | 5 | 65148736 | 65482027 | 1 | 1 | 123579 | 0.002504 | 0.499 |
| 22941 | 11 | 70467856 | 71252738 | 1 | 1 | 123579 | -0.00276 | 0.5011 |
| 64682 | 2 | 1.12E+08 | 1.12E+08 | 1 | 1 | 123579 | -0.01153 | 0.5046 |
| 79949 | 10 | 1.14E+08 | 1.14E+08 | 1 | 1 | 123579 | -0.01454 | 0.5058 |
| 25831 | 14 | 31100115 | 31208060 | 1 | 1 | 123579 | -0.0188 | 0.5075 |
| 4285 | 13 | 23730188 | 23889448 | 1 | 1 | 123579 | -0.02657 | 0.5106 |
| 6675 | 1 | 1.63E+08 | 1.63E+08 | 1 | 1 | 123579 | -0.03586 | 0.5143 |
| 344148 | 2 | 1.33E+08 | 1.34E+08 | 1 | 1 | 123579 | -0.0361 | 0.5144 |
| 10082 | 13 | 93226825 | 94408020 | 2 | 1 | 123579 | -0.04841 | 0.5193 |
| 1740 | 11 | 83455012 | 85628534 | 2 | 1 | 123579 | -0.04933 | 0.51967 |
| 11317 | 20 | 45306824 | 45319677 | 1 | 1 | 123579 | -0.06396 | 0.5255 |
| 129642 | 2 | 8852690 | 9003747 | 1 | 1 | 123579 | -0.06447 | 0.5257 |
| 400499 | 16 | 11372601 | 11523533 | 1 | 1 | 123579 | -0.06597 | 0.5263 |
| 1633 | 4 | 70993548 | 71030914 | 2 | 1 | 123579 | -0.06814 | 0.52716 |
| 7371 | 1 | 1.66E+08 | 1.66E+08 | 1 | 1 | 123579 | -0.06974 | 0.5278 |
| 285195 | 3 | 1.43E+08 | 1.44E+08 | 1 | 1 | 123579 | -0.07376 | 0.5294 |
| 6546 | 2 | 40112146 | 40512486 | 1 | 1 | 123579 | -0.07728 | 0.5308 |
| 5209 | 10 | 6144880 | 6235545 | 2 | 1 | 123579 | -0.08371 | 0.53335 |
| 22904 | 19 | 1107634 | 1174283 | 1 | 1 | 123579 | -0.08432 | 0.5336 |
| 57002 | 7 | 39566376 | 39612089 | 1 | 1 | 123579 | -0.0881 | 0.5351 |
| 767 | 8 | 60187347 | 60281395 | 1 | 1 | 123579 | -0.10094 | 0.5402 |
| 9369 | 14 | 78021758 | 79868290 | 2 | 1 | 123579 | -0.10138 | 0.54038 |
| 5818 | 11 | 1.2E+08 | 1.2E+08 | 1 | 1 | 123579 | -0.10522 | 0.5419 |
| 1131 | 1 | 2.39E+08 | 2.4E+08 | 2 | 1 | 123579 | -0.1055 | 0.54201 |
| 11063 | 5 | 1.58E+08 | 1.58E+08 | 1 | 1 | 123579 | -0.10825 | 0.5431 |
| 23071 | 9 | 99979179 | 1E+08 | 1 | 1 | 123579 | -0.11405 | 0.5454 |
| 55507 | 12 | 12940554 | 12979517 | 1 | 1 | 123579 | -0.11405 | 0.5454 |
| 23396 | 19 | 3630181 | 3700492 | 1 | 1 | 123579 | -0.11581 | 0.5461 |
| 728215 | 13 | 1.07E+08 | 1.08E+08 | 1 | 1 | 123579 | -0.11708 | 0.5466 |
| 653489 | 2 | 1.06E+08 | 1.06E+08 | 1 | 1 | 123579 | -0.12718 | 0.5506 |
| 3687 | 16 | 31355134 | 31382997 | 1 | 1 | 123579 | -0.13122 | 0.5522 |
| 79875 | 15 | 71115628 | 71783383 | 1 | 1 | 123579 | -0.13198 | 0.5525 |
| 6000 | 1 | 2.41E+08 | 2.41E+08 | 1 | 1 | 123579 | -0.13299 | 0.5529 |
| 8992 | 5 | 1.73E+08 | 1.73E+08 | 1 | 1 | 123579 | -0.13375 | 0.5532 |
| 2212 | 1 | 1.62E+08 | 1.62E+08 | 2 | 1 | 123579 | -0.14301 | 0.55686 |
| 631 | 20 | 17493905 | 17569220 | 1 | 1 | 123579 | -0.14362 | 0.5571 |
| 165918 | 3 | 1.96E+08 | 1.97E+08 | 1 | 1 | 123579 | -0.14463 | 0.5575 |
| 4815 | 12 | 564296 | 663741 | 1 | 1 | 123579 | -0.14615 | 0.5581 |
| 55615 | 22 | 44668547 | 44737681 | 1 | 1 | 123579 | -0.14742 | 0.5586 |
| 83700 | 11 | 1.34E+08 | 1.34E+08 | 1 | 1 | 123579 | -0.15249 | 0.5606 |
| 3117 | 6 | 32637396 | 32654774 | 1 | 1 | 123579 | -0.15604 | 0.562 |
| 5329 | 19 | 43646095 | 43670346 | 1 | 1 | 123579 | -0.16213 | 0.5644 |
| 8939 | 9 | 1.31E+08 | 1.31E+08 | 1 | 1 | 123579 | -0.17255 | 0.5685 |
| 23074 | 12 | 1E+08 | 1E+08 | 1 | 1 | 123579 | -0.17459 | 0.5693 |
| 57655 | 19 | 34994784 | 35026471 | 1 | 1 | 123579 | -0.18478 | 0.5733 |
| 27133 | 14 | 62706573 | 63045256 | 1 | 1 | 123579 | -0.18682 | 0.5741 |
| 57584 | 10 | 24583609 | 24723668 | 1 | 1 | 123579 | -0.18937 | 0.5751 |
| 5933 | 20 | 36996349 | 37096000 | 1 | 1 | 123579 | -0.19422 | 0.577 |
| 54585 | 3 | 45823316 | 45915724 | 1 | 1 | 123579 | -0.19525 | 0.5774 |
| 9750 | 6 | 24804281 | 25042288 | 1 | 1 | 123579 | -0.19857 | 0.5787 |
| 3799 | 10 | 32009010 | 32056443 | 1 | 1 | 123579 | -0.20599 | 0.5816 |
| 196513 | 12 | 1946048 | 2004511 | 1 | 1 | 123579 | -0.21419 | 0.5848 |
| 8289 | 1 | 26696031 | 26782110 | 1 | 1 | 123579 | -0.2165 | 0.5857 |
| 23026 | 13 | 1.08E+08 | 1.09E+08 | 1 | 1 | 123579 | -0.22163 | 0.5877 |
| 3786 | 8 | 1.32E+08 | 1.32E+08 | 1 | 1 | 123579 | -0.22523 | 0.5891 |
| 4763 | 17 | 31007873 | 31377677 | 1 | 1 | 123579 | -0.23321 | 0.5922 |
| 57677 | 19 | 36334453 | 36379203 | 1 | 1 | 123579 | -0.23759 | 0.5939 |
| 11187 | 11 | 392599 | 404908 | 1 | 1 | 123579 | -0.24611 | 0.5972 |
| 130271 | 2 | 43637300 | 43767987 | 1 | 1 | 123579 | -0.26008 | 0.6026 |
| 78989 | 2 | 3594832 | 3644644 | 1 | 1 | 123579 | -0.26371 | 0.604 |
| 23386 | 7 | 44382366 | 44490878 | 1 | 1 | 123579 | -0.26683 | 0.6052 |
| 55530 | 12 | 1.09E+08 | 1.09E+08 | 1 | 1 | 123579 | -0.26683 | 0.6052 |
| 3728 | 17 | 41754607 | 41786712 | 1 | 1 | 123579 | -0.26683 | 0.6052 |
| 9844 | 7 | 36852906 | 37449409 | 1 | 1 | 123579 | -0.26865 | 0.6059 |
| 27156 | 22 | 23059408 | 23145230 | 1 | 1 | 123579 | -0.27151 | 0.607 |
| 2781 | 22 | 23070483 | 23125034 | 1 | 1 | 123579 | -0.27151 | 0.607 |
| 5925 | 13 | 48303747 | 48481890 | 1 | 1 | 123579 | -0.27463 | 0.6082 |
| 10161 | 13 | 48411045 | 48444704 | 1 | 1 | 123579 | -0.27463 | 0.6082 |
| 1E+08 | 3 | 46753043 | 46812420 | 1 | 1 | 123579 | -0.28479 | 0.6121 |
| 81035 | 18 | 319355 | 500729 | 1 | 1 | 123579 | -0.28846 | 0.6135 |
| 821 | 5 | 1.8E+08 | 1.8E+08 | 1 | 1 | 123579 | -0.30364 | 0.6193 |
| 10559 | 6 | 87472925 | 87512339 | 1 | 1 | 123579 | -0.30469 | 0.6197 |
| 57038 | 6 | 87499347 | 87590017 | 1 | 1 | 123579 | -0.30469 | 0.6197 |
| 883 | 9 | 1.29E+08 | 1.29E+08 | 1 | 1 | 123579 | -0.31232 | 0.6226 |
| 55247 | 4 | 1.77E+08 | 1.77E+08 | 1 | 1 | 123579 | -0.31838 | 0.6249 |
| 79912 | 12 | 21437604 | 21471250 | 1 | 1 | 123579 | -0.32154 | 0.6261 |
| 92235 | 1 | 1.67E+08 | 1.67E+08 | 3 | 2 | 123579 | -0.32831 | 0.62866 |
| 1806 | 1 | 97077743 | 97921059 | 1 | 1 | 123579 | -0.33212 | 0.6301 |
| 933 | 19 | 35329166 | 35347361 | 1 | 1 | 123579 | -0.3353 | 0.6313 |
| 152189 | 3 | 32238679 | 32370321 | 1 | 1 | 123579 | -0.33822 | 0.6324 |
| 6801 | 2 | 36837698 | 36966557 | 1 | 1 | 123579 | -0.34353 | 0.6344 |
| 3535 | 22 | 22026076 | 22922913 | 1 | 1 | 123579 | -0.34592 | 0.6353 |
| 56980 | 11 | 1.3E+08 | 1.3E+08 | 1 | 1 | 123579 | -0.34646 | 0.6355 |
| 9228 | 8 | 1019561 | 1708476 | 1 | 1 | 123579 | -0.35873 | 0.6401 |
| 5081 | 1 | 18631006 | 18748866 | 1 | 1 | 123579 | -0.3598 | 0.6405 |
| 116987 | 2 | 2.35E+08 | 2.36E+08 | 1 | 1 | 123579 | -0.36113 | 0.641 |
| 8522 | 17 | 9910609 | 10198551 | 1 | 1 | 123579 | -0.36569 | 0.6427 |
| 255104 | 1 | 19682213 | 19800353 | 1 | 1 | 123579 | -0.3767 | 0.6468 |
| 7525 | 18 | 721588 | 812326 | 1 | 1 | 123579 | -0.37858 | 0.6475 |
| 4651 | 5 | 16661907 | 16936276 | 1 | 1 | 123579 | -0.37966 | 0.6479 |
| 774 | 9 | 1.38E+08 | 1.38E+08 | 1 | 1 | 123579 | -0.38289 | 0.6491 |
| 51334 | 5 | 1.2E+08 | 1.21E+08 | 1 | 1 | 123579 | -0.38451 | 0.6497 |
| 26053 | 7 | 69598475 | 70793068 | 2 | 1 | 123579 | -0.39608 | 0.65398 |
| 6957 | 7 | 1.42E+08 | 1.43E+08 | 1 | 1 | 123579 | -0.40076 | 0.6557 |
| 9411 | 1 | 94168907 | 94275068 | 1 | 1 | 123579 | -0.40565 | 0.6575 |
| 5797 | 18 | 7567316 | 8406861 | 1 | 1 | 123579 | -0.4111 | 0.6595 |
| 79718 | 3 | 1.77E+08 | 1.77E+08 | 1 | 1 | 123579 | -0.41219 | 0.6599 |
| 84303 | 3 | 1.27E+08 | 1.27E+08 | 1 | 1 | 123579 | -0.4193 | 0.6625 |
| 5866 | 11 | 61897240 | 61946311 | 1 | 1 | 123579 | -0.43219 | 0.6672 |
| 2013 | 16 | 10528422 | 10580682 | 1 | 1 | 123579 | -0.43577 | 0.6685 |
| 6195 | 1 | 26529758 | 26575030 | 1 | 1 | 123579 | -0.43633 | 0.6687 |
| 152002 | 3 | 1.95E+08 | 1.95E+08 | 1 | 1 | 123579 | -0.43936 | 0.6698 |
| 26038 | 1 | 6101787 | 6180134 | 1 | 1 | 123579 | -0.44323 | 0.6712 |
| 134526 | 5 | 81329991 | 81394169 | 1 | 1 | 123579 | -0.44489 | 0.6718 |
| 441459 | 9 | 33524353 | 33575969 | 1 | 1 | 123579 | -0.45988 | 0.6772 |
| 64759 | 7 | 47275144 | 47582553 | 1 | 1 | 123579 | -0.46127 | 0.6777 |
| 26037 | 14 | 71321396 | 71741229 | 1 | 1 | 123579 | -0.46407 | 0.6787 |
| 147872 | 19 | 49388218 | 49417999 | 1 | 1 | 123579 | -0.47498 | 0.6826 |
| 1112 | 14 | 89156172 | 89619150 | 1 | 1 | 123579 | -0.48116 | 0.6848 |
| 56647 | 10 | 1.26E+08 | 1.26E+08 | 1 | 1 | 123579 | -0.49132 | 0.6884 |
| 55760 | 10 | 1.26E+08 | 1.26E+08 | 1 | 1 | 123579 | -0.49132 | 0.6884 |
| 29994 | 2 | 1.59E+08 | 1.6E+08 | 1 | 1 | 123579 | -0.49358 | 0.6892 |
| 11122 | 20 | 42072752 | 43189917 | 1 | 1 | 123579 | -0.49472 | 0.6896 |
| 81839 | 1 | 1.16E+08 | 1.16E+08 | 1 | 1 | 123579 | -0.50494 | 0.6932 |
| 4645 | 18 | 49822786 | 50195081 | 1 | 1 | 123579 | -0.50836 | 0.6944 |
| 441061 | 5 | 16067365 | 16179788 | 1 | 1 | 123579 | -0.51379 | 0.6963 |
| 6660 | 12 | 23529495 | 24562669 | 1 | 1 | 123579 | -0.51464 | 0.6966 |
| 117583 | 2 | 2.05E+08 | 2.06E+08 | 2 | 1 | 123579 | -0.52669 | 0.70079 |
| 80790 | 16 | 81445170 | 81711762 | 2 | 1 | 123579 | -0.52897 | 0.70159 |
| 1496 | 2 | 79512934 | 80648868 | 1 | 1 | 123579 | -0.53392 | 0.7033 |
| 96459 | 5 | 1.32E+08 | 1.32E+08 | 1 | 1 | 123579 | -0.54116 | 0.7058 |
| 1607 | 7 | 14145049 | 14974777 | 1 | 1 | 123579 | -0.54552 | 0.7073 |
| 55790 | 8 | 19404161 | 19757271 | 1 | 1 | 123579 | -0.55338 | 0.71 |
| 79977 | 8 | 1.01E+08 | 1.02E+08 | 1 | 1 | 123579 | -0.55631 | 0.711 |
| 55793 | 1 | 1.51E+08 | 1.51E+08 | 1 | 1 | 123579 | -0.56953 | 0.7155 |
| 10371 | 7 | 83956805 | 84492724 | 1 | 1 | 123579 | -0.57336 | 0.7168 |
| 223 | 1 | 1.66E+08 | 1.66E+08 | 1 | 1 | 123579 | -0.57484 | 0.7173 |
| 55351 | 4 | 5051211 | 5500998 | 1 | 1 | 123579 | -0.58047 | 0.7192 |
| 157807 | 8 | 60966438 | 61501645 | 1 | 1 | 123579 | -0.58106 | 0.7194 |
| 7143 | 1 | 1.75E+08 | 1.76E+08 | 2 | 1 | 123579 | -0.58399 | 0.72039 |
| 9508 | 4 | 72280969 | 72568799 | 1 | 1 | 123579 | -0.58939 | 0.7222 |
| 1.05E+08 | 1 | 1.82E+08 | 1.82E+08 | 1 | 1 | 123579 | -0.60497 | 0.7274 |
| 5021 | 3 | 8750408 | 8854549 | 1 | 1 | 123579 | -0.60647 | 0.7279 |
| 9229 | 18 | 3496032 | 4455310 | 1 | 1 | 123579 | -0.611 | 0.7294 |
| 55690 | 11 | 66070353 | 66244747 | 1 | 1 | 123579 | -0.6274 | 0.7348 |
| 5536 | 19 | 46346994 | 46390975 | 1 | 1 | 123579 | -0.63014 | 0.7357 |
| 113 | 16 | 50244749 | 50318135 | 1 | 1 | 123579 | -0.63137 | 0.7361 |
| 23348 | 13 | 98793487 | 99088618 | 1 | 1 | 123579 | -0.64458 | 0.7404 |
| 288 | 10 | 60026298 | 60733526 | 1 | 1 | 123579 | -0.65169 | 0.7427 |
| 6654 | 2 | 38981549 | 39124744 | 1 | 1 | 123579 | -0.652 | 0.7428 |
| 27127 | 22 | 45344063 | 45413629 | 2 | 1 | 123579 | -0.67634 | 0.75059 |
| 5745 | 3 | 46877717 | 46903799 | 2 | 1 | 123579 | -0.67661 | 0.75067 |
| 27044 | 7 | 1.28E+08 | 1.28E+08 | 1 | 1 | 123579 | -0.68016 | 0.7518 |
| 3763 | 21 | 37617482 | 37919731 | 2 | 1 | 123579 | -0.68236 | 0.75249 |
| 114800 | 2 | 56184123 | 56386174 | 1 | 1 | 123579 | -0.68238 | 0.7525 |
| 25937 | 3 | 1.5E+08 | 1.5E+08 | 1 | 1 | 123579 | -0.68396 | 0.753 |
| 286343 | 9 | 12774990 | 12823060 | 1 | 1 | 123579 | -0.69286 | 0.7558 |
| 79632 | 6 | 1.19E+08 | 1.19E+08 | 1 | 1 | 123579 | -0.6954 | 0.7566 |
| 10477 | 2 | 1.81E+08 | 1.81E+08 | 1 | 1 | 123579 | -0.697 | 0.7571 |
| 9103 | 1 | 1.62E+08 | 1.62E+08 | 1 | 1 | 123579 | -0.70277 | 0.7589 |
| 387700 | 10 | 89430294 | 89535556 | 1 | 1 | 123579 | -0.70341 | 0.7591 |
| 25817 | 22 | 48489460 | 48751935 | 1 | 1 | 123579 | -0.70405 | 0.7593 |
| 54715 | 16 | 5239782 | 7713340 | 3 | 1 | 123579 | -0.71072 | 0.76137 |
| 5788 | 1 | 1.99E+08 | 1.99E+08 | 1 | 1 | 123579 | -0.71437 | 0.7625 |
| 124989 | 17 | 47323961 | 47441312 | 1 | 1 | 123579 | -0.71437 | 0.7625 |
| 53353 | 2 | 1.4E+08 | 1.42E+08 | 1 | 1 | 123579 | -0.74248 | 0.7711 |
| 57107 | 6 | 1.07E+08 | 1.07E+08 | 1 | 1 | 123579 | -0.74611 | 0.7722 |
| 266722 | 13 | 96090536 | 96839562 | 1 | 1 | 123579 | -0.75275 | 0.7742 |
| 144404 | 12 | 1.22E+08 | 1.22E+08 | 1 | 1 | 123579 | -0.75542 | 0.775 |
| 23108 | 17 | 2755698 | 3037741 | 1 | 1 | 123579 | -0.76311 | 0.7773 |
| 221154 | 13 | 21492689 | 21604216 | 1 | 1 | 123579 | -0.77118 | 0.7797 |
| 221264 | 6 | 1.09E+08 | 1.1E+08 | 1 | 1 | 123579 | -0.79468 | 0.7866 |
| 3776 | 1 | 2.15E+08 | 2.15E+08 | 1 | 1 | 123579 | -0.80123 | 0.7885 |
| 23140 | 17 | 4004445 | 4143020 | 1 | 1 | 123579 | -0.8092 | 0.7908 |
| 8170 | 18 | 45212957 | 45683107 | 1 | 1 | 123579 | -0.82495 | 0.7953 |
| 64097 | 5 | 1.12E+08 | 1.12E+08 | 1 | 1 | 123579 | -0.82671 | 0.7958 |
| 9901 | 3 | 8980591 | 9362929 | 1 | 1 | 123579 | -0.8345 | 0.798 |
| 3681 | 16 | 31393241 | 31426513 | 1 | 1 | 123579 | -0.83699 | 0.7987 |
| 54780 | 10 | 1.22E+08 | 1.22E+08 | 1 | 1 | 123579 | -0.84627 | 0.8013 |
| 6049 | 13 | 26132115 | 26222559 | 1 | 1 | 123579 | -0.85275 | 0.8031 |
| 150684 | 2 | 61888728 | 62136070 | 1 | 1 | 123579 | -0.86909 | 0.8076 |
| 152110 | 3 | 27107336 | 27369460 | 1 | 1 | 123579 | -0.87863 | 0.8102 |
| 84240 | 5 | 81301583 | 81313344 | 1 | 1 | 123579 | -0.91842 | 0.8208 |
| 4045 | 3 | 1.16E+08 | 1.16E+08 | 1 | 1 | 123579 | -0.92763 | 0.8232 |
| 63917 | 7 | 1.52E+08 | 1.52E+08 | 1 | 1 | 123579 | -0.9514 | 0.8293 |
| 8470 | 4 | 1.86E+08 | 1.86E+08 | 1 | 1 | 123579 | -0.95377 | 0.8299 |
| 375775 | 9 | 1.37E+08 | 1.38E+08 | 1 | 1 | 123579 | -0.96011 | 0.8315 |
| 5067 | 3 | 74262568 | 74614445 | 1 | 1 | 123579 | -0.98709 | 0.8382 |
| 55531 | 11 | 1.08E+08 | 1.08E+08 | 1 | 1 | 123579 | -0.99118 | 0.8392 |
| 283420 | 12 | 10030677 | 10066030 | 1 | 1 | 123579 | -0.99446 | 0.84 |
| 55599 | 1 | 1.04E+08 | 1.04E+08 | 1 | 1 | 123579 | -1.0002 | 0.8414 |
| 144448 | 12 | 85014311 | 85036278 | 1 | 1 | 123579 | -1.0322 | 0.849 |
| 56975 | 7 | 192777 | 260774 | 1 | 1 | 123579 | -1.0394 | 0.8507 |
| 149297 | 1 | 1.66E+08 | 1.66E+08 | 1 | 1 | 123579 | -1.0481 | 0.8527 |
| 23274 | 16 | 10944488 | 11193278 | 4 | 1 | 123579 | -1.0532 | 0.85387 |
| 346007 | 6 | 63719980 | 65707225 | 1 | 1 | 123579 | -1.0537 | 0.854 |
| 6095 | 15 | 60488284 | 61229303 | 1 | 1 | 123579 | -1.0634 | 0.8562 |
| 197322 | 16 | 89093809 | 89155846 | 1 | 1 | 123579 | -1.0767 | 0.8592 |
| 10396 | 4 | 42408373 | 42657105 | 1 | 1 | 123579 | -1.0839 | 0.8608 |
| 91526 | 2 | 1.97E+08 | 1.97E+08 | 1 | 1 | 123579 | -1.1044 | 0.8653 |
| 84937 | 16 | 74999017 | 75110994 | 1 | 1 | 123579 | -1.1133 | 0.8672 |
| 84991 | 10 | 6088986 | 6117459 | 1 | 1 | 123579 | -1.1151 | 0.8676 |
| 5139 | 12 | 20368510 | 20687641 | 1 | 1 | 123579 | -1.117 | 0.868 |
| 152877 | 4 | 1574062 | 1684313 | 1 | 1 | 123579 | -1.1217 | 0.869 |
| 441631 | 12 | 30926449 | 31016490 | 1 | 1 | 123579 | -1.1455 | 0.874 |
| 64409 | 7 | 71132414 | 71713601 | 1 | 1 | 123579 | -1.1489 | 0.8747 |
| 56999 | 3 | 64515654 | 64688000 | 1 | 1 | 123579 | -1.1494 | 0.8748 |
| 26033 | 10 | 1.15E+08 | 1.16E+08 | 1 | 1 | 123579 | -1.169 | 0.8788 |
| 376497 | 19 | 17468774 | 17506168 | 1 | 1 | 123579 | -1.17 | 0.879 |
| 55775 | 14 | 89954935 | 90044764 | 1 | 1 | 123579 | -1.179 | 0.8808 |
| 3241 | 2 | 10302889 | 10427617 | 1 | 1 | 123579 | -1.182 | 0.8814 |
| 1.02E+08 | 17 | 2724411 | 2749562 | 1 | 1 | 123579 | -1.1856 | 0.8821 |
| 10223 | 1 | 1.67E+08 | 1.67E+08 | 1 | 1 | 123579 | -1.1861 | 0.8822 |
| 54778 | 15 | 58987666 | 59097419 | 1 | 1 | 123579 | -1.1896 | 0.8829 |
| 3516 | 4 | 26163494 | 26435131 | 1 | 1 | 123579 | -1.1978 | 0.8845 |
| 5991 | 9 | 3218297 | 3526001 | 1 | 1 | 123579 | -1.1978 | 0.8845 |
| 1.02E+08 | 6 | 32255717 | 32407822 | 2 | 1 | 123579 | -1.2207 | 0.8889 |
| 10665 | 6 | 32288541 | 32372114 | 2 | 1 | 123579 | -1.2207 | 0.8889 |
| 23114 | 1 | 2.05E+08 | 2.05E+08 | 1 | 1 | 123579 | -1.2297 | 0.8906 |
| 55714 | 4 | 1.82E+08 | 1.83E+08 | 1 | 1 | 123579 | -1.2432 | 0.8931 |
| 3033 | 4 | 1.08E+08 | 1.08E+08 | 1 | 1 | 123579 | -1.2804 | 0.8998 |
| 1.05E+08 | 1 | 1.62E+08 | 1.62E+08 | 1 | 1 | 123579 | -1.2867 | 0.9009 |
| 158038 | 9 | 27937617 | 29213981 | 1 | 1 | 123579 | -1.2873 | 0.901 |
| 7424 | 4 | 1.77E+08 | 1.77E+08 | 1 | 1 | 123579 | -1.2965 | 0.9026 |
| 157869 | 8 | 73064543 | 73093272 | 1 | 1 | 123579 | -1.3047 | 0.904 |
| 1501 | 5 | 10971840 | 11904043 | 2 | 1 | 123579 | -1.3082 | 0.90459 |
| 23360 | 11 | 47716517 | 47767460 | 1 | 1 | 123579 | -1.3141 | 0.9056 |
| 23309 | 19 | 16829387 | 16880355 | 1 | 1 | 123579 | -1.3334 | 0.9088 |
| 7090 | 15 | 70047790 | 70097935 | 1 | 1 | 123579 | -1.3383 | 0.9096 |
| 60526 | 2 | 20684014 | 20823137 | 1 | 1 | 123579 | -1.3426 | 0.9103 |
| 7922 | 6 | 33200826 | 33204437 | 1 | 1 | 123579 | -1.3445 | 0.9106 |
| 3710 | 6 | 33621379 | 33696574 | 1 | 1 | 123579 | -1.3519 | 0.9118 |
| 10818 | 12 | 69470349 | 69579793 | 1 | 1 | 123579 | -1.3633 | 0.9136 |
| 84749 | 12 | 1.09E+08 | 1.09E+08 | 1 | 1 | 123579 | -1.3658 | 0.914 |
| 26011 | 11 | 78653283 | 79440989 | 1 | 1 | 123579 | -1.3696 | 0.9146 |
| 400954 | 2 | 54723670 | 54972908 | 1 | 1 | 123579 | -1.3904 | 0.9178 |
| 121274 | 12 | 48335491 | 48351414 | 1 | 1 | 123579 | -1.3917 | 0.918 |
| 204219 | 15 | 1E+08 | 1.01E+08 | 1 | 1 | 123579 | -1.4031 | 0.9197 |
| 7029 | 3 | 1.42E+08 | 1.42E+08 | 1 | 1 | 123579 | -1.4091 | 0.9206 |
| 9441 | 19 | 16574907 | 16628204 | 1 | 1 | 123579 | -1.4105 | 0.9208 |
| 56242 | 19 | 19865830 | 19894449 | 1 | 1 | 123579 | -1.4242 | 0.9228 |
| 7018 | 3 | 1.34E+08 | 1.34E+08 | 1 | 1 | 123579 | -1.4339 | 0.9242 |
| 4916 | 15 | 87874999 | 88256796 | 1 | 1 | 123579 | -1.4481 | 0.9262 |
| 23504 | 12 | 1.3E+08 | 1.31E+08 | 1 | 1 | 123579 | -1.4713 | 0.9294 |
| 7148 | 6 | 32041155 | 32109374 | 1 | 1 | 123579 | -1.4743 | 0.9298 |
| 115825 | 13 | 51584194 | 51766799 | 1 | 1 | 123579 | -1.5309 | 0.9371 |
| 5214 | 10 | 3067474 | 3136805 | 1 | 1 | 123579 | -1.5423 | 0.9385 |
| 83696 | 8 | 1.4E+08 | 1.4E+08 | 1 | 1 | 123579 | -1.5565 | 0.9402 |
| 5073 | 16 | 14435700 | 14630275 | 1 | 1 | 123579 | -1.6 | 0.9452 |
| 51059 | 8 | 1.38E+08 | 1.38E+08 | 2 | 1 | 123579 | -1.6007 | 0.94528 |
| 2898 | 6 | 1.01E+08 | 1.02E+08 | 1 | 1 | 123579 | -1.6164 | 0.947 |
| 79925 | 5 | 35617779 | 35814611 | 1 | 1 | 123579 | -1.622 | 0.9476 |
| 154141 | 6 | 20099684 | 20212464 | 1 | 1 | 123579 | -1.6286 | 0.9483 |
| 388650 | 1 | 92832729 | 92961522 | 1 | 1 | 123579 | -1.6497 | 0.9505 |
| 27244 | 6 | 1.09E+08 | 1.09E+08 | 1 | 1 | 123579 | -1.6686 | 0.9524 |
| 26003 | 2 | 1.71E+08 | 1.71E+08 | 1 | 1 | 123579 | -1.6954 | 0.955 |
| 65059 | 2 | 2.03E+08 | 2.04E+08 | 1 | 1 | 123579 | -1.7093 | 0.9563 |
| 4301 | 6 | 1.68E+08 | 1.68E+08 | 1 | 1 | 123579 | -1.7147 | 0.9568 |
| 51101 | 8 | 78666047 | 78719765 | 1 | 1 | 123579 | -1.7449 | 0.9595 |
| 3574 | 8 | 78675872 | 78805523 | 1 | 1 | 123579 | -1.7449 | 0.9595 |
| 440279 | 15 | 53978203 | 54628608 | 1 | 1 | 123579 | -1.7732 | 0.9619 |
| 3029 | 16 | 1809103 | 1827194 | 1 | 1 | 123579 | -1.8224 | 0.9658 |
| 9043 | 17 | 50962174 | 51120865 | 1 | 1 | 123579 | -1.8592 | 0.9685 |
| 9612 | 12 | 1.24E+08 | 1.25E+08 | 1 | 1 | 123579 | -1.8649 | 0.9689 |
| 7991 | 8 | 15540087 | 15766649 | 1 | 1 | 123579 | -1.911 | 0.972 |
| 84969 | 20 | 43914852 | 44069616 | 1 | 1 | 123579 | -1.9142 | 0.9722 |
| 90693 | 7 | 23597379 | 23644708 | 1 | 1 | 123579 | -2.0065 | 0.9776 |
| 1.01E+08 | 6 | 31399784 | 31415315 | 2 | 1 | 123579 | -2.0736 | 0.98094 |
| 9743 | 11 | 1.29E+08 | 1.29E+08 | 1 | 1 | 123579 | -2.1107 | 0.9826 |
| 753 | 18 | 13218730 | 13652754 | 1 | 1 | 123579 | -2.1545 | 0.9844 |
| 51112 | 2 | 3379672 | 3479571 | 1 | 1 | 123579 | -2.2768 | 0.9886 |
| 26027 | 1 | 54548134 | 54634744 | 1 | 1 | 123579 | -2.2801 | 0.9887 |
| 9837 | 20 | 25407444 | 25448555 | 1 | 1 | 123579 | -2.2835 | 0.9888 |
| 9751 | 20 | 1266297 | 1309327 | 1 | 1 | 123579 | -2.3495 | 0.9906 |
| 8792 | 18 | 62325287 | 62387710 | 1 | 1 | 123579 | -2.4677 | 0.9932 |
| 4004 | 11 | 8224304 | 8268635 | 1 | 1 | 123579 | -2.4893 | 0.9936 |
| 6753 | 22 | 37204237 | 37220447 | 1 | 1 | 123579 | -2.5828 | 0.9951 |
| 1805 | 1 | 1.69E+08 | 1.69E+08 | 1 | 1 | 123579 | -2.6437 | 0.9959 |
| 636 | 12 | 32106736 | 32383650 | 1 | 1 | 123579 | -2.7822 | 0.9973 |

**Table S7 Gene-based analysis Based on MAGMA**

| GENE | CHR | ZSTAT | P |
| --- | --- | --- | --- |
| MCTP1 | 5 | 3.4565 | 0.000274 |
| ANKRD32 | 5 | 3.4565 | 0.000274 |
| MTHFS | 15 | 3.2389 | 0.0006 |
| ST20-MTHFS | 15 | 3.2389 | 0.0006 |
| KATNAL2 | 18 | 2.979 | 0.001446 |
| RTN4RL1 | 17 | 2.5891 | 0.004811 |
| ASCC3 | 6 | 2.576 | 0.004997 |
| DUSP22 | 6 | 2.5548 | 0.005313 |
| LOC105369145 | 6 | 2.5451 | 0.005463 |
| CTU2 | 16 | 2.5255 | 0.005776 |
| EXT1 | 8 | 2.5051 | 0.00612 |
| OSBPL8 | 12 | 2.4932 | 0.00633 |
| MANBA | 4 | 2.3977 | 0.008249 |
| ITPR1 | 3 | 2.3044 | 0.0106 |
| ESR1 | 6 | 2.2678 | 0.01167 |
| TXNDC16 | 14 | 2.2218 | 0.01315 |
| PRSS22 | 16 | 2.1675 | 0.0151 |
| CTDSPL2 | 15 | 2.1656 | 0.01517 |
| LRRTM3 | 10 | 2.153 | 0.01566 |
| HPSE2 | 10 | 2.1205 | 0.01698 |
| LOC285423 | 4 | 2.0486 | 0.02025 |
| MICU1 | 10 | 2.015 | 0.02195 |
| SELE | 1 | 2.0024 | 0.02262 |
| ZNF510 | 9 | 1.9998 | 0.02276 |
| PSMB2 | 1 | 1.9027 | 0.02854 |
| ABR | 17 | 1.8989 | 0.02879 |
| KIAA1671 | 22 | 1.8785 | 0.030159 |
| ATXN10 | 22 | 1.842 | 0.03274 |
| MEF2C | 5 | 1.8361 | 0.03317 |
| F5 | 1 | 1.8101 | 0.03514 |
| PAQR7 | 1 | 1.7636 | 0.0389 |
| SLC4A4 | 4 | 1.7585 | 0.03933 |
| CTNNA3 | 10 | 1.7411 | 0.040835 |
| PHOSPHO2-KLHL23 | 2 | 1.7143 | 0.04324 |
| HDLBP | 2 | 1.699 | 0.04466 |
| LOC101927509 | 2 | 1.699 | 0.04466 |
| NOS1AP | 1 | 1.6894 | 0.045572 |
| C1orf116 | 1 | 1.6538 | 0.049079 |
